# A novel necroptosis-related lncRNA signature for predicting prognosis and immune response of colon cancer

**DOI:** 10.3389/fgene.2022.984696

**Published:** 2022-08-25

**Authors:** Jian Luo, Jiayu Peng, Wanying Xiao, Shujing Huang, Yanqing Cao, Ting Wang, Xicheng Wang

**Affiliations:** ^1^ Department of Oncology, The First Affiliated Hospital of Guangdong Pharmaceutical University, Guangdong Pharmaceutical University, Guangzhou, China; ^2^ Department of Radiation, Sun Yat-sen University Cancer Center, Guangzhou, China

**Keywords:** necroptosis, lncRNA, immune, colon cancer, nomogram

## Abstract

**Background:** Numerous lncRNAs have been shown to affect colon cancer (CC) progression, and tumor necroptosis is regulated by several of them. However, the prognostic value of necroptosis-related lncRNA in CC has rarely been reported. In this study, a necroptosis-related lncRNA prognostic model was constructed, which can provide a reference for clinical diagnosis and treatment.

**Methods:** The Cancer Genome Atlas (TCGA) database provided gene expression and lncRNA sequencing data for CC patients, and GSEA provided necroptosis gene data. Differentially expressed necroptosis-related lncRNAs related to prognosis were identified by differential expression analysis, Pearson correlation analysis, and least absolute shrinkage and selection operator (LASSO) regression. Based on the results of the multivariate COX regression analysis, a risk scoring model was constructed, A Kaplan-Meier analysis was performed to compare overall survival (OS) between low-risk and high-risk groups. A nomogram was then developed and validated based on the clinical data and risk scores of CC patients. In addition, Gene Set Enrichment Analysis (GSEA) and immune correlation analysis were conducted to explore the possible pathways and immune regulatory effects of these necroptosis-related lncRNAs.

**Results:** In total, we identified 326 differentially expressed necroptosis-related lncRNAs in the TCGA database. Survival analysis showed that the OS of patients in the low-risk group was significantly better than that in the high-risk group (*p* < 0.05). Finally, 10 prognostic necroptosis-related lncRNAs were used to construct the nomogram. The composite nomogram prediction model evaluated and validated with good prediction performance (3-year AUC = 0.85, 5-years AUC = 0.82, C-index = 0.78). The GSEA and immune correlation analyses indicated that these lncRNAs may participate in multiple pathways involved in CC pathogenesis and progression.

**Conclusion:** We established a novel necroptosis-related lncRNA CC prognosis prediction model, which can provide a reference for clinicians to formulate personalized treatment and review plans for CC patients. In addition, we also found that these necroptosis-related lncRNAs may affect the pathogenesis and progression of colon cancer through multiple pathways, including altering the activity of various immune cells.

## Introduction

Colon cancer (CC) is one of the most common gastrointestinal malignancies (Siegel et al., 2021). According to statistics, the number of new colorectal cancer cases in the world in 2020 ranked third among all cancers, and the mortality rate ranked second (Sung et al., 2021). The treatment of CC mainly consists of surgery, chemotherapy and radiotherapy (Benson et al., 2021). Since most patients are initially diagnosed as advanced CC and often with distant metastases, long-term survival of most CC patients is unsatisfactory. Accurately assessing the prognosis of CC patients will be of great help to clinicians in formulating individualized treatment and review plans for CC patients. At present, clinicians mainly evaluate the prognosis based on the TNM staging and pathological typing of CC. With the rapid development of immunotherapy and targeted therapy, great breakthroughs have been made in the treatment of CC. The prognostic model constructed based on CC genetic data and immune response information will have a good auxiliary role in the prognosis evaluation of CC.

Necroptosis, a type of programmed necrotic cell apoptosis, is the gatekeeper of the host defense against pathogen invasion. Previous studies have reported that necroptosis plays an important role in the occurrence and progression of various autoimmune diseases, inflammatory diseases and tumors (Negroni et al., 2020). The effect of necroptosis on tumors is dual: on the one hand, the pathway of necroptosis can promote tumor metastasis and progression by acting alone or in combination (McCormick et al., 2016; Strilic et al., 2016). On the one hand, necroptosis-related pathways can promote tumor proliferation and migration by acting alone or in combination (5, 6); on the other hand, necroptosis is also a “fail-safe” mechanism when apoptosis is impaired, thereby inhibiting tumor progression (Höckendorf et al., 2016). In previous studies on necroptosis and CC, Liu et al. (2019) found that necroptosis receptor-interacting protein 3 (PIP3) has the potential to promote colitis-related CC progression by promoting tumor cell proliferation and CXCL1-induced immunosuppression.

LncRNAs are a class of non-coding RNAs between 200–100,000 nt in length that do not encode proteins but are involved in the regulation of various intracellular processes (Guttman et al., 2009), They are involved in the regulation of tumors including: cell proliferation and migration, differentiation and development, apoptosis and necrosis (Bhan and Mandal 2014). Similarly, lncRNAs also play an important regulatory role in the pathogenesis and progression of CC (Bhan et al., 2017). Cheng et al. (2020) found that lncRNA LINC00662 regulates CLDN8/IL22 co-expression and activates ERK signaling pathway to promote the growth and metastasis of CC. Huang et al. (2017) found that the lncRNA-encoded peptide HOXB-AS3 inhibits the growth of CC tumor cells.

Various lncRNAs are thought to be responsible for necrotic apoptosis, which significantly influences CC pathogenesis, we speculate that necroptosis-related lncRNAs have the potential to serve as prognostic predictors of CC. Several prognostic models of Necroptosis-related lncRNAs have been developed and applied in gastric cancer, ovarian cancer, breast cancer and other tumors, and achieved good results (Zhao et al., 2021; Zhang et al., 2022; Zhu et al., 2022). However, there are few reports on the relationship between Necroptosis-related lncRNAs and CC. Our study established a CC prognostic model of Necroptosis-related lncRNAs, which can assist clinicians in evaluating the prognosis of CC patients. We also preliminarily analyzed the impact of these lncRNAs on the immune response of CC and the pathways they may be involved in, which can provide new ideas for the next research direction.

## Materials and methods

### Data sources and processing

**FIGURE 1:**
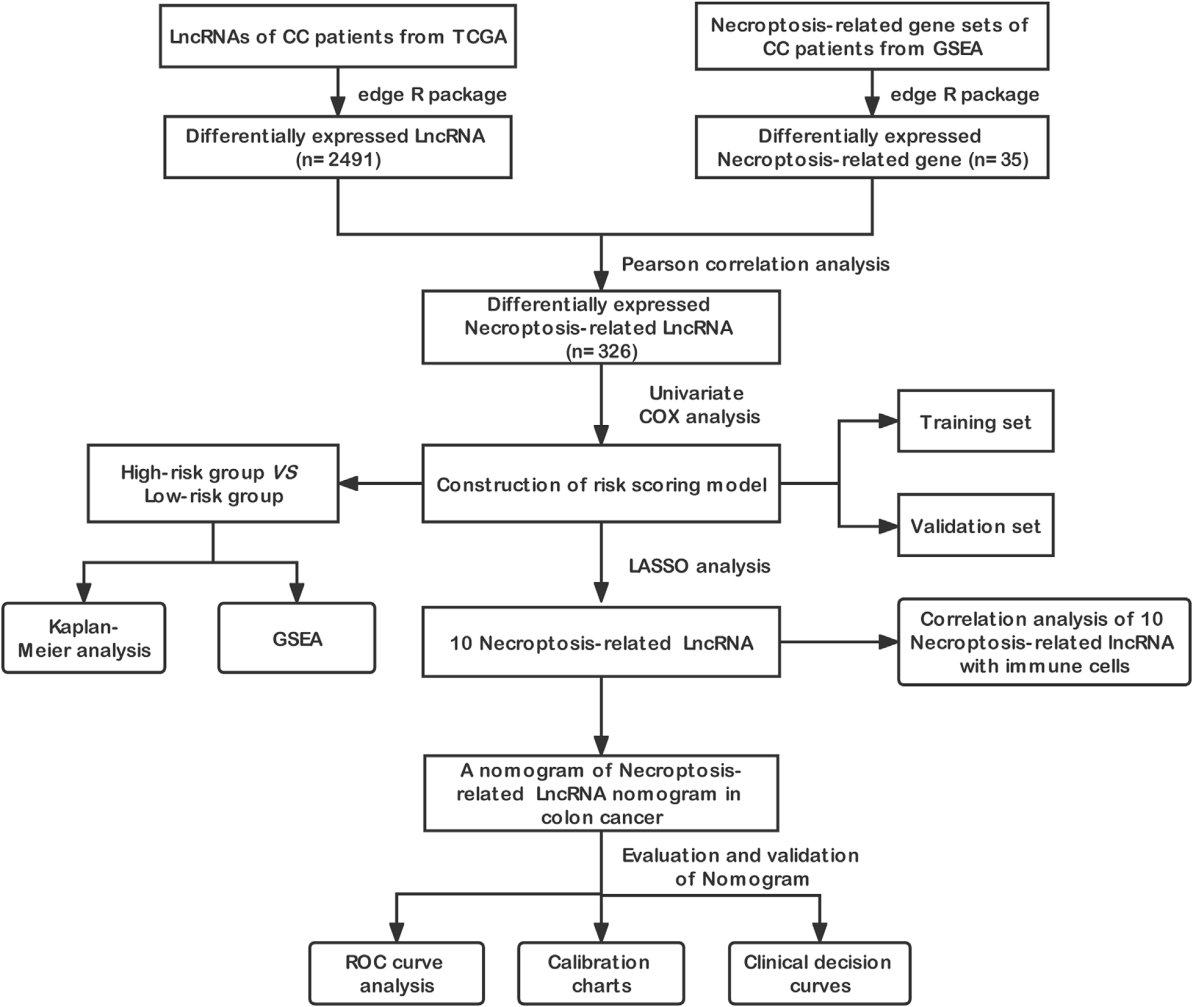
Flowchart showing the main steps of this study.

First, we drew a flow chart roughly showing the main steps of this study (Figure 1). The RNA sequencing data and clinical characteristics of CC patients were obtained from The Cancer Genome Atlas (TCGA) database (https://www.cancer.gov/), and the necroptosis-related gene set data were downloaded from the GSEA official website (www.gsea-msigdb.org/). get. The data were normalized using the “edgeR” package of R, and the differentially expressed necroptosis-related genes and differentially expressed lncRNAs were obtained through differential expression analysis in the tumor tissue and adjacent tissue of CC patients, and a volcano plot of the differentially expressed genes was constructed. (|log2FC| > 1, FDR <0.05. The differentially expressed necroptosis-related genes and lncRNAs were selected for pearson correlation analysisto obtain the differentially expressed necroptosis-related lncRNAs (|R^2^| > 0.3 and *p* < 0.05).

### Construction and validation of risk scoring model

Necroptosis-related lncRNAs associated with prognosis were identified by univariate Cox regression (*p* < 0.05). To reduce overfitting, we performed least absolute shrinkage and selection operator (LASSO) regression. Finally, 10 differentially expressed necroptosis-related lncRNAs related to prognosis were obtained to construct a nomogram. Multivariate Cox regression analysis was used to calculate the correlation coefficient between necroptosis and lncRNA, and a risk scoring model composed of correlation coefficient and lncRNA expression level was constructed as follows (***β***_***i***_: correlation coefficient, ***Exp***_***i***_: lncRNA expression level):

$$\mathrm{Risk score=}\overset{10}{\underset{i=1}{\sum }}\left(\beta_{i}*\mathrm{Ex}p_{i}\right)$$

All CC patients with complete clinical baseline data for more than 30 days of follow-up were randomly divided into training set and validation set according to the ratio of 2:1. The risk score model was used to score each CC patient, and then the risk scores of all patients were ranked from high to low, and the median was taken as the cutoff value, and they were divided into two groups: high risk and low risk. Survival analysis was then performed to compare the difference in OS between the two groups of CC patients. Risk curves were used to show the distribution of risk scores for CC patients in the high-risk and low-risk groups, and scatter plots showed survival status for each CC patient, and the expression levels of selected lncRNAs are displayed using heat maps.

### Nomogram construction and OS prediction

Patients’ age, gender, race, TNM stage and other clinical data and risk scores were included for univariate and multivariate Cox analysis to assess the value of risk scores and clinical characteristics as independent prognostic factors for CC patients. Based on the above results, we established a CC prognostic nomogram of necroptosis-related lncRNAs. The 3-years and 5-years overall survival rates in the nomogram were used to estimate the prognostic information of CC patients. The area under the curve (AUC) value in the receiver operating characteristic curve (ROC) I was used to evaluate the accuracy of the nomogram. Subsequently, the performance of the prognostic model was validated by decision curve analysis (DCA) and calibration curves. We avalidated the above results using the validation set data.

### Gene set enrichment analysis

Using GSEA_4.2.3, the gene set function and pathway enrichment analysis was performed on the differentially expressed genes in the high-risk group and the low-risk group, and the differences in biological functions or signaling pathways of these differentially expressed genes were explored.

### Correlation analysis between 10 necroptosis-related lncRNA and immune cells

22 types immune cells associated with CC prognosis were identified by CIBERSORT analysis. The expression levels of 22 types of immune cells such as T cells CD4 and CD8, B cells naïve, NK cells and Macrophages in CC patients. Then, Pearson correlation analysis was conducted to clarify the correlation between these prognostic necroptosis-related lncRNAs and immune cell response, and to explore the association between necroptosis-related lncRNAs and immune response of CC and the mechanism of tumor progression regulation.

### Statistical analysis methods

All statistical analyses in this study were performed using R (4.1.3) and the R package. Pearson correlation analysis was used to determine the association of two different variables. Student’s t test was used for parametric variables, chi-square test and Mann-Whitney U test were used for nonparametric variables. Univariate Cox regression analysis and multivariate Cox regression analysis were used to find risk factors affecting OS, and LASSO regression analysis was used to reduce overfitting. Kaplan-Meier analysis was used to compare OS, and ROC curves were used to evaluate the sensitivity and specificity of the model. p value less than 0.05 was considered statistically significant.

## Results

### Differentially expressed necroptosis-related genes and lncRNAs in colon cancer

**FIGURE 2:**
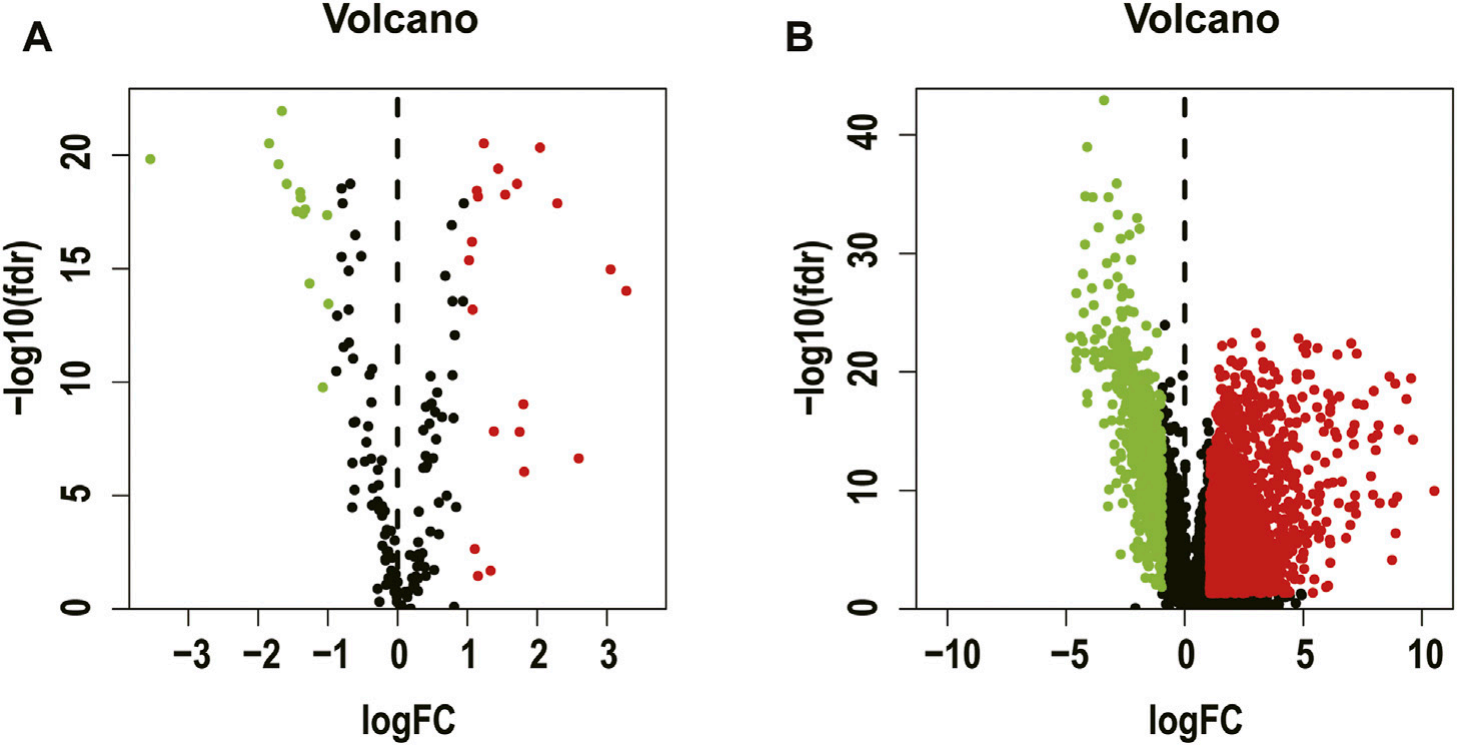
Volcano plot showing genes differentially expressed in colon cancer versus para-carcinoma tissue. Necroptosis-related genes **(A)**, lncRNA **(B)**.

A total of 410 CC patients’ cancer tissues and 41 paracancerous tissues' clinical data and gene set data were obtained from the TCGA database, and necroptosis-related gene sets were provided in the GSEA. After differential expression analysis, we identified a total of 35 differentially expressed necroptosis-related genes (Supplementary Table S1) and 2491 differentially expressed lncRNAs (Supplementary Table S2), which were presented in a volcano plot (Figure 2).

### Identification of differentially expressed necroptosis-related lncRNAs

**FIGURE 3:**
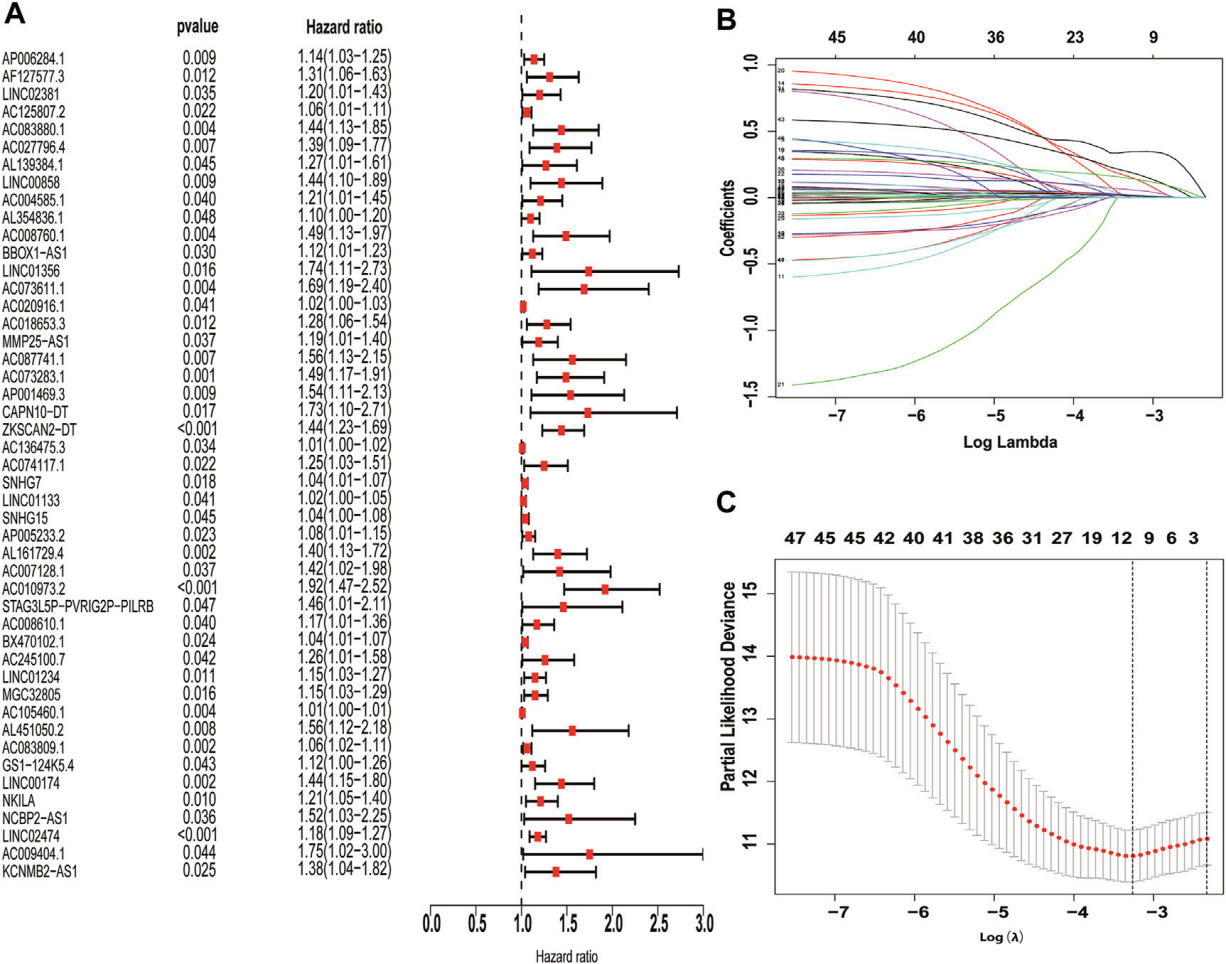
Screening of necroptosis-related lncRNAs differentially expressed in colon cancer. Necroptosis-related lncRNAs with prognostic significance were screened by univariate COX regression **(A)**, LASSO regression was used to reduce overfitting **(B,C)**.

The correlation coefficient was set to |R2 | > 0.3 and *p* < 0.05, and 326 necroptosis-related lncRNAs were further screened by Pearson correlation analysis (Supplementary Table S3). Finally, 10 necroptosis-related LncRNAs with prognostic significance (AC083880.1, AC073611.1, SNHG7, LINC01133, AP005233.2, AC010973.2, LINC01234, AC083809.1, NKILA, LINC02474) were obtained through univariate cox regression and LASSO analysis. All these necroptosis-related lncRNAs are pathogenic factors of CC (Figure 3).

### Construction and validation of necroptosis-related lncRNA risk model

**FIGURE 4:**
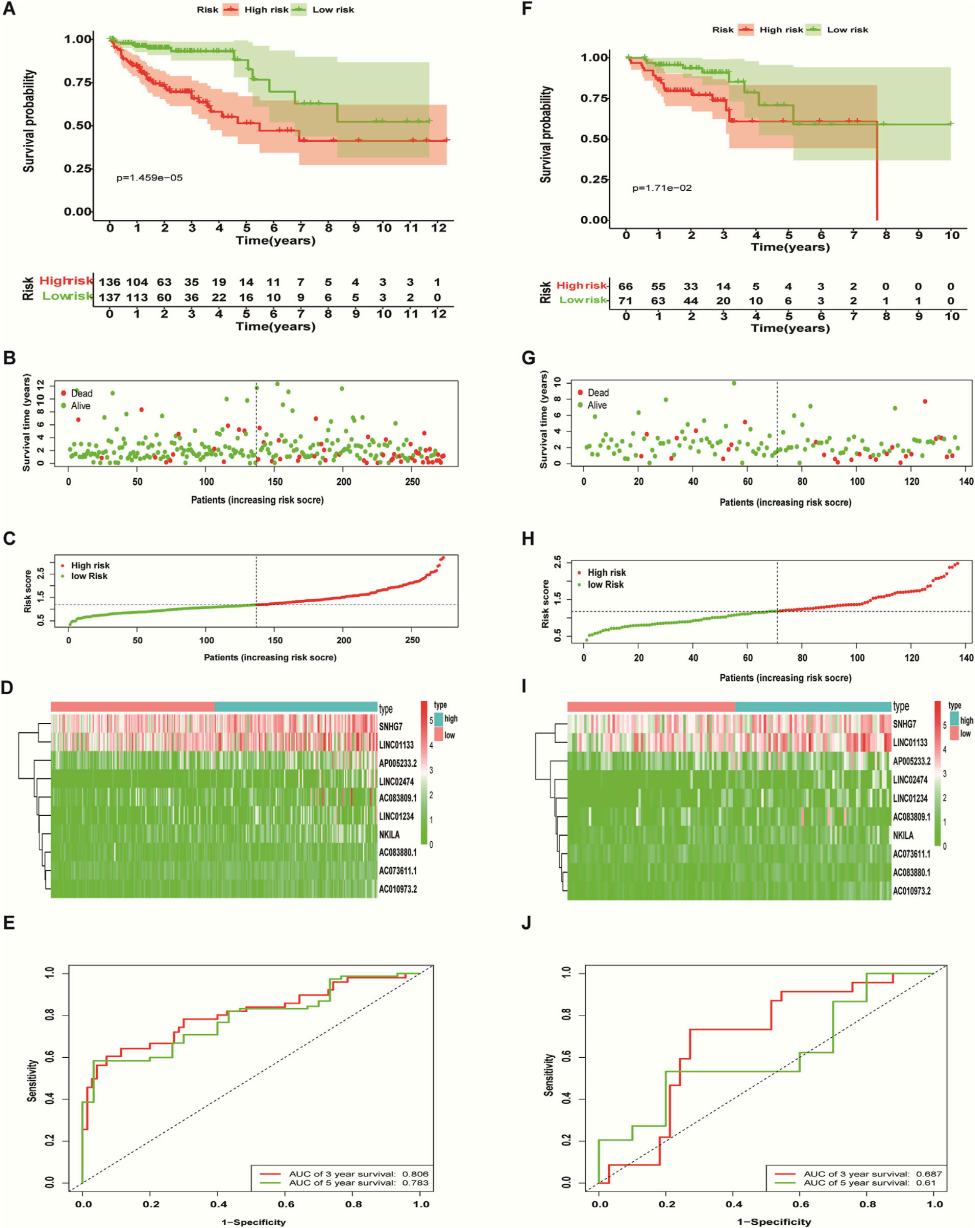
Predictive performance of a necroptosis-related lncRNA risk model. The Kaplan-Meier curve results **(A,F)**, scatter plots **(B,G)** and survival status curves **(C,H)** all showed that the OS of colon cancer patients in the high-risk group is significantly inferior to that in the low-risk group. The heat map showed that the abundance of differentially expressed necroptosis-related lncRNAs correlated with prognosis **(D,I)**, and the ROC curves showed the three- and 5-years AUC values **(E,J)**. Training set **(A–E)**, validation set **(F-J)**.

**FIGURE 5:**
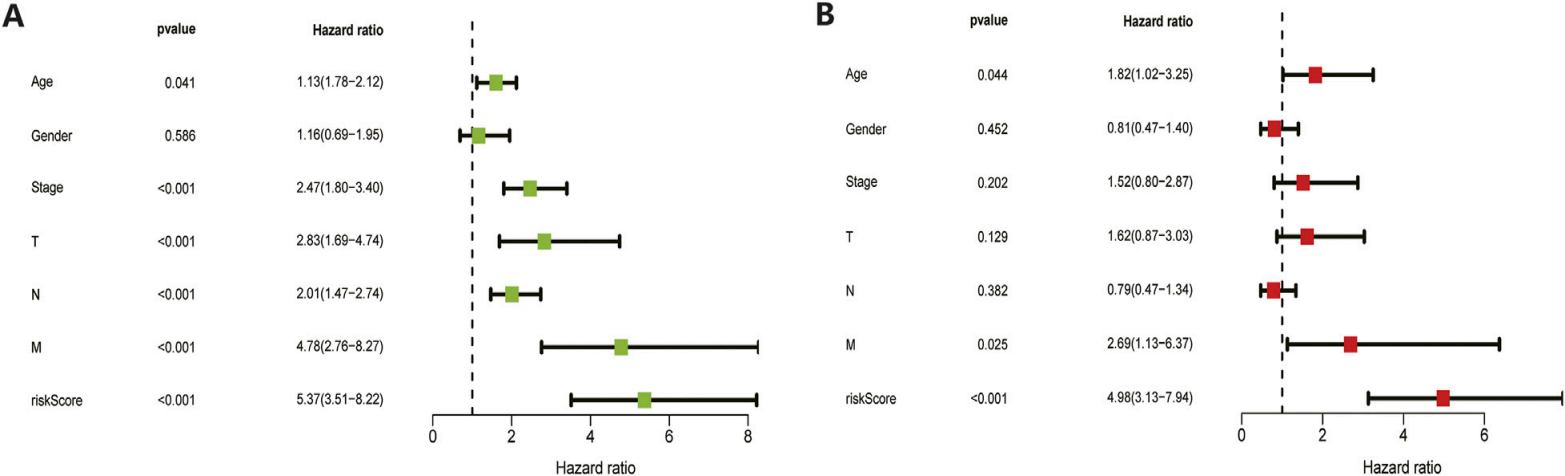
Univariate and multivariate COX regression analysis results. Both training set **(A)** and validation set **(B)** results showed that the model’s risk scores is an independent prognostic factor for colon cancer.

The 410 CC patients with complete clinical baseline data and gene set data were divided into training set and validation set for model construction and validation, and the ratio of training set and validation set was 2:1. Kaplan-meier survival analysis showed that the OS of patients in the high-risk group was significantly shorter than that in the low-risk group (*p* < 0.01, Figures 4A,F. The ROC curve shows that the AUC value of our model is 0.81 in 3 years and 0.78 in 5 years, indicating good prediction effect (Figures 4 E,J). Univariate and multivariate Cox regression analysis showed the effect of risk score and clinical parameters on patient prognosis, and the results suggested that risk score was an independent influencing factor after CC (Figures 5A,B. All the above results were validated by the validation set data.

### Establishment and evaluation of necroptosis-related lncRNA nomogram

**FIGURE 6:**
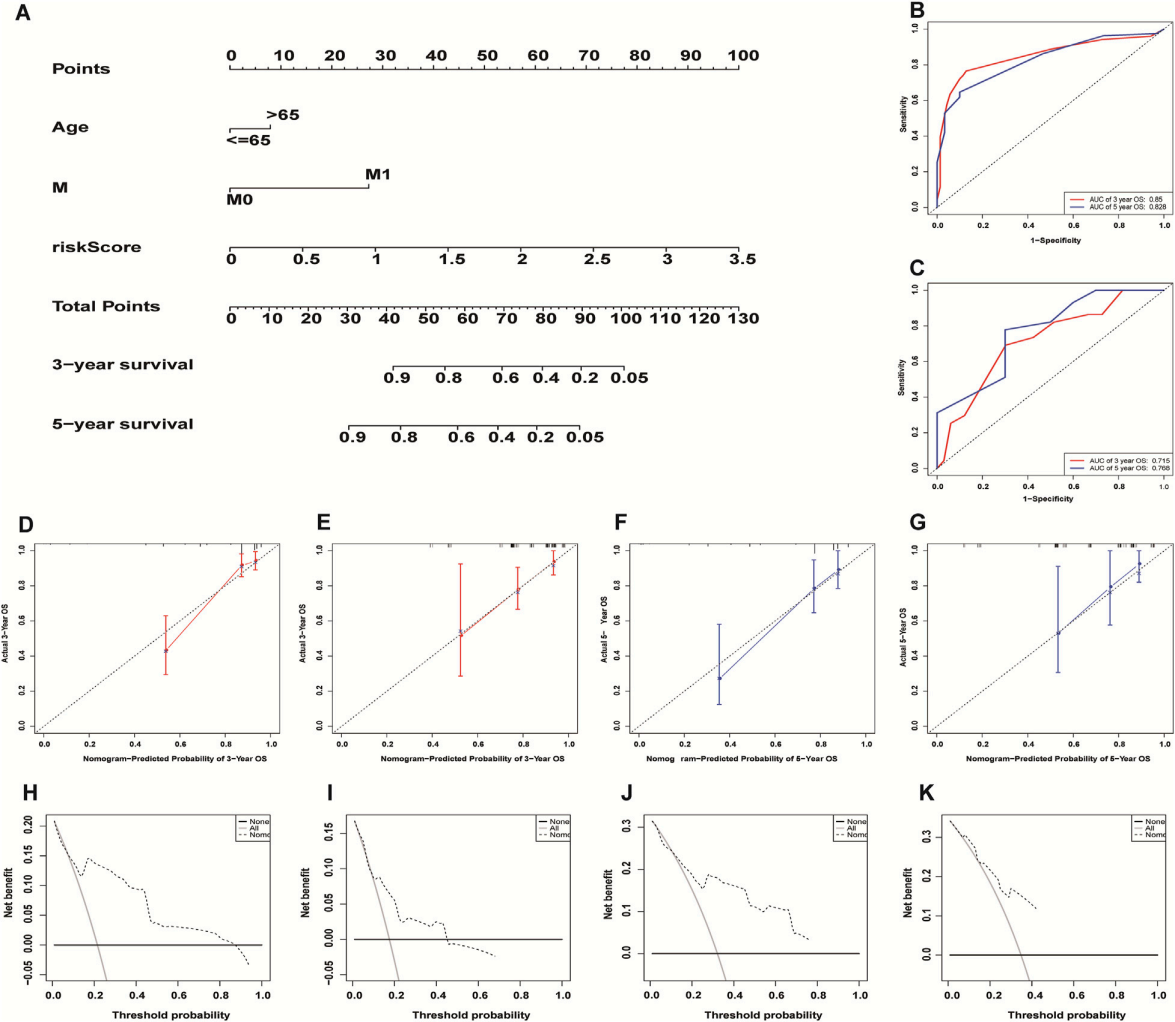
Construction and evaluation of necroptosis-related lncRNA nomogram. A composite nomogram model for predicting OS in patients with colon cancer **(A)**. The ROC curve shows that the nomogram has good predictive performance in the training set **(B)** and validation set **(C)**. Calibration Curve **(D,E,F,G)** and Decision Curve Analyses **(H,I,J,K)** validated the predictive performance of the nomogram on the training set **(D,F,H,J)** and validation set **(E,G,I,K)**.

**FIGURE 7:**
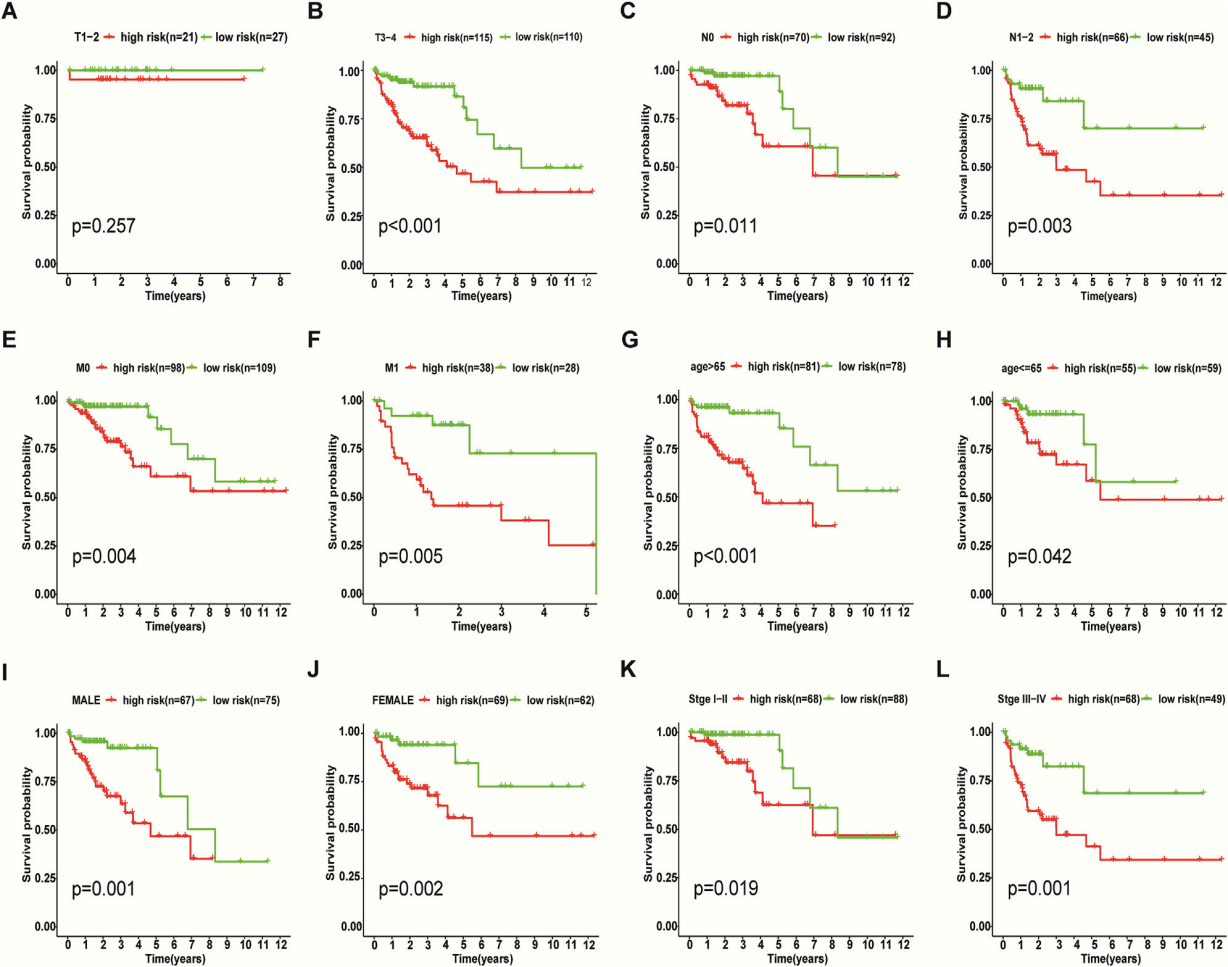
Results of Subgroup Analysis. Among T1 and T2 stage of colon cancer patients, subgroup analysis indicated that there was no significant difference in survival between the low-risk and high-risk groups (*p* > 0.05) **(A)**, while the other subgroups showed that the OS of the high-risk group was worse than that of the low-risk group (*p* < 0.05) **(B–L)**.

We incorporated risk scores and clinical characteristics such as age, gender, and TMN stage into the final prognostic model. 10 prognostic necroptosis-related lncRNAs were used to construct a nomogram. Incorporating the clinical data and risk scores of CC patients into this nomogram yields their 3- and 5-years overall survival rates (Figure 6A. Additionally, we drawn ROC curves and obtain AUC values (3-years AUC = 0.85, 5-years AUC = 0.82) to assess the accuracy of the nomogram (Figures 6B,C). C-index: 0.78 for training set, 0.73 for validation set. DCA and calibration curves verified the good predictive performance of our predictive model (Figures 6 D–K). Finally, all the above results are verified by the validation set data. A subgroup analysis showed no significant difference in OS between high-risk and low-risk groups at T1 and T2 stages (*p* > 0.05), we speculate that the possible reason is that the number of patients with T1 and T2 stages is small (21 cases in the high-risk group and 27 cases in the low-risk group) (Figure 7A. The results of other subgroup analyses indicated that the OS of CC patients in the high-risk group was significantly inferior to that in the low-risk group (p < 0.05) (Figures 7B–L).

### Gene-set function and pathway enrichment analysis

**FIGURE 8:**
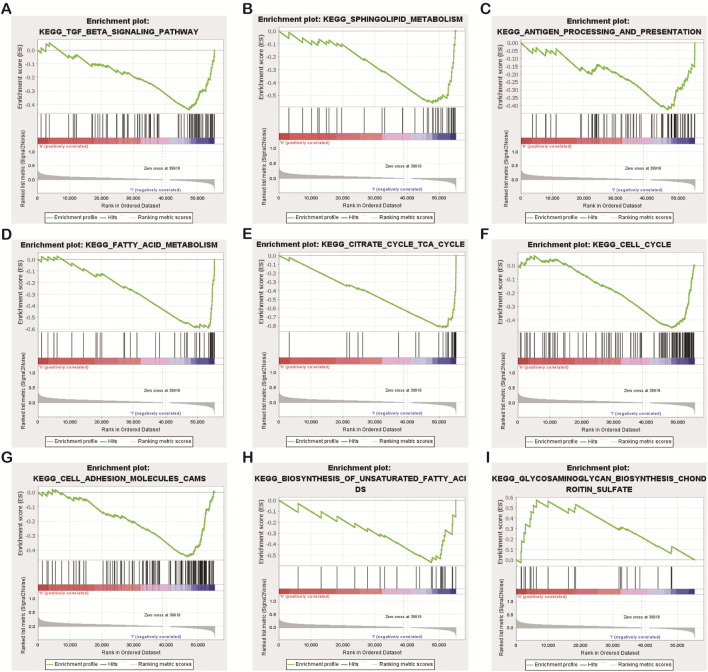
Results of functional and pathway enrichment analysis of gene sets. Necroptosis-related lncRNAs were significantly enriched in the high-risk group **(A–H)**, While significantly enriched in the low-risk group **(I)**.

Some interesting findings were also made by biological function and pathway enrichment analysis of differentially expressed genes in low-risk and high-risk groups: these differentially expressed necrotic apoptosis-related lncrnas may be involved in gene recombination of CC cells, proliferation of tumor cells, antigen processing and presentation, and nutrient metabolism (Figures 8A−I). These findings point the way for our next research.

### Correlation analysis of necroptosis-related lncRNAs and tumor immune cells response

**FIGURE 9:**
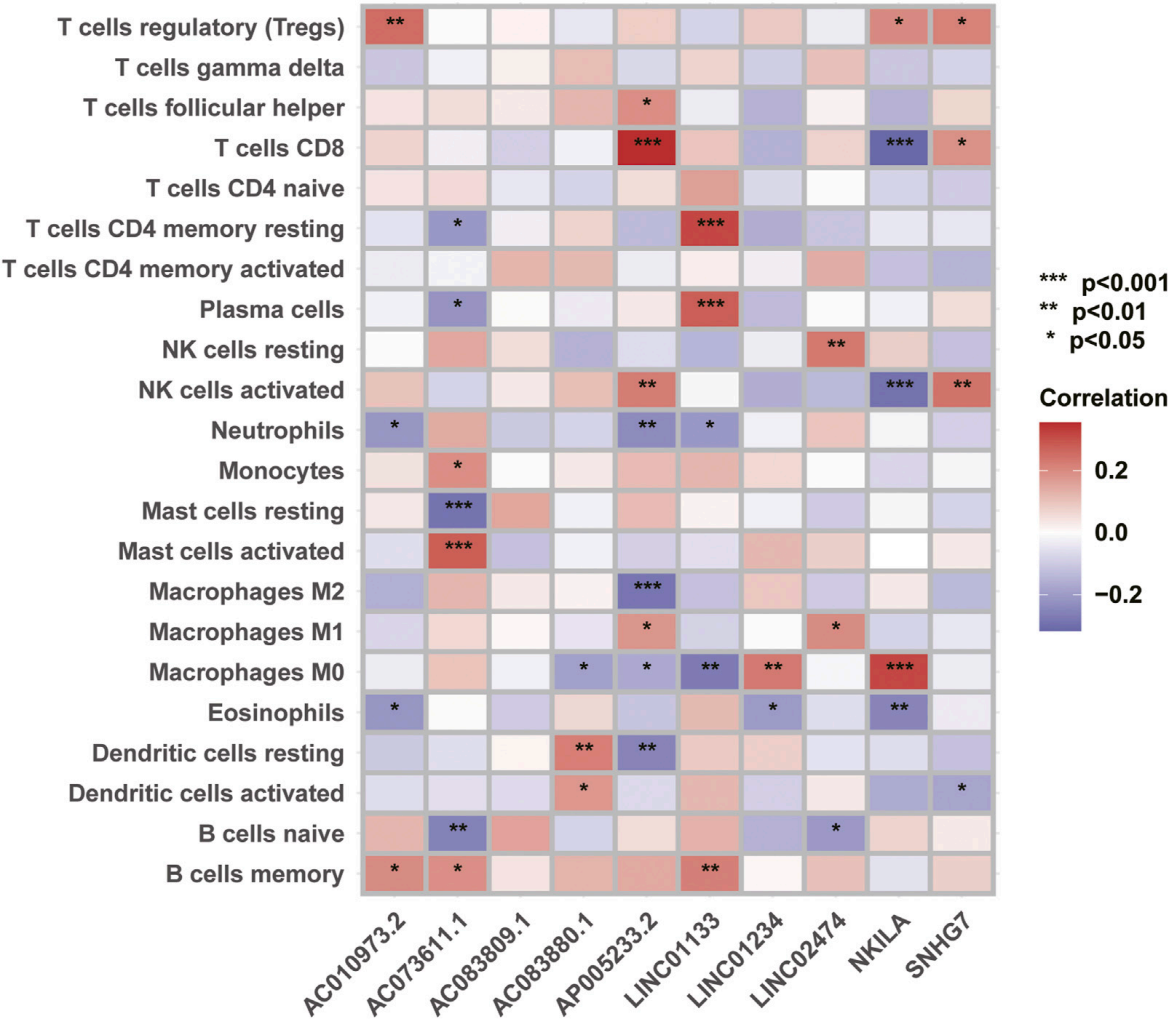
Correlation analysis between necroptosis-related lncRNAs and tumor immune function.

We used the immune correlation analysis tool CIBERSORT to analyze the immune response of 22 types of immune cells in CC. The results indicated that the abundance of 10 necroptosis-related lncRNAs used to build the model was significantly correlated with the activity of various immune cells. For example: lncRNA NKILA was significantly negatively correlated with the activity of T cells CD8, and lncRNA NKILA could promote the polarization of Macrophages M0; the abundance of lncRNA LINC01133 was positively correlated with the activity of T cells CD4 memory resting and Plasma cells, and the polarization of Macrophages M0 Negative correlation; the abundance of lncRNA AC073611.1 was positively correlated with Mast cells activation, but negatively correlated with Mast cells resting (Figure 9). These results suggest that necroptosis-related lncRNAs may affect the tumor immune infiltration microenvironment to regulate tumor growth and progression by promoting or inhibiting the activity of CD4 and CD8 cells and the activation of mast cells.

## Discussion

CC has the characteristics of high morbidity, poor prognosis and expensive treatment, and the OS of CC patients is often shortened due to recurrence and metastasis. Tumor TNM staging, pathological diagnosis and genetic testing results are the main basis for surgery, radiotherapy and chemotherapy in patients with CC, but there is a lack of quantitative risk score prediction methods. Therefore, evaluating the prognostic information of patients in advance is of great value for the attending physician to choose the treatment plan and follow-up plan suitable for the CC patient. Some studies have confirmed that a variety of lncRNAs can affect the occurrence and development of CC by regulating cell proliferation, apoptosis and death, cell cycle, cell migration and invasion ability, epithelial-mesenchymal transition (EMT), and chemotherapy resistance (Chen and Shen 2020; Ni et al., 2020). In addition, some necroptosis-related genes regulated by multiple lncRNAs have also been shown to contribute in CC progression and immune response (Seo et al., 2021).

This study identified 10 necroptosis-related LncRNAs (AC083880.1, AC073611.1, SNHG7, LINC01133, AP005233.2, AC010973.2, LINC01234, AC083809.1, NKILA, LINC02474) that are associated with CC prognosis, all these necrosis-related lncRNAs are unfavorable prognostic factors for CC prognosis. We have drawn a nomogram based on necroptosis-related lncRNAs, which can well assess the prognosis of patients and provide a new tool for the prognosis assessment of CC patients. Compared with previous lncRNA-related CC prediction models, our prediction model was validated to be more accurate (Our model 3-years AUC = 0.85 VS. Liu L’s 0.72) (Liu et al., 2022).

By analyzing the correlation between necrotizing apoptosis-related lncrnas and immune responses, we found some lncrnas previously reported in previous studies, as well as some new findings. Our study found that lncRNA NKILA was negatively correlated with T cells CD8 and NK cells activated, while Macrophages M0 was positively correlated with polarization and secretion. Chen et al. (2022) s study also found that lncRNA NKILA et al. was related to the immune invasion of CC, but the specific immune regulatory pathway is still unclear. Furthermore, we found that LncRNA 01234 was highly expressed in CC patients and positively correlated with the polarization of Macrophages M0. Lin C et al. also found that LncRNA 01234 was highly expressed in CC patients, and the OS of those with high LncRNA 01234 expression was significantly shortened (Lin et al., 2019). Our study also found that lncRNA SNHG7 is highly expressed in CC patients, which is consistent with previous research conclusions. Other studies have also found that some lncRNAs are associated with distant metastasis and cisplatin resistance in CC (Yu et al., 2017; Shan et al., 2018). In this study, we also discovered some new lncRNAs: AC083880.1, AC073611.1, LINC01133, AP005233.2, AC010973.2, AC083809.1, LINC02474. Although there are no relevant basic research reports on these lncRNAs, their correlation analysis results with immune response suggest that these lncRNAs may affect the progression of CC by changing the activities of macrophages, mast cells, T cells CD8, T cells CD4, NK cells, and other immune cells. These results suggest that, these lncRNAs may be a new direction in the study of lncRNA and CC.

In terms of gene functions and pathways involved in necroptosis-related lncRNAs, previous studies have found that genetic variation in the citric acid cycle and fatty acid metabolism are associated with the onset and progression of CC (Choi et al., 2019; Cho et al., 2020). Chondroitin sulfate has certain effects on the proliferation and apoptosis of CC (Wu et al., 2021). The GSEA of this study found that the differentially expressed necroptosis-related lncRNAs in the high-risk group and the low-risk group were involved in the process of gene recombination, tumor cell proliferation, antigen processing and presentation, and nutrient metabolism in CC, thereby affecting the occurrence and progression of CC. These findings can provide new research directions for us to study the etiology and treatment of CC.

It is also true that there are some limitations to this study. The first is that the data of this study are from the TCGA database, and the patient population is mainly Americans. People from different countries and races have different dietary habits, CC genotypes may also be different, and CC patients may have different prognosis. In the next step of the study, we will collect multi-center Chinese population data for analysis to see if there are consistent conclusions. Secondly, this study is a retrospective analysis. Although the predictive model we constructed has good predictive performance through internal validation, multi-center prospective data are still needed to further confirm its predictive performance.

## Conclusion

This study clarified the relationship between necroptosis-related lncRNAs and CC prognosis, and constructed a CC prognosis model based on 10 necroptosis-related lncRNAs. The model has been verified to have good predictive performance, which can provide a reference for clinicians when choosing appropriate treatment and follow-up plans for CC patients. In addition, we also explored the gene functions of necroptosis-related lncRNAs, the signaling pathways that may be involved, and the correlation of tumor immune regulation, providing new directions for future research on the etiology and treatment of CC.

## Data Availability

Publicly available datasets were analyzed in this study. The names of the repository/repositories and accession number(s) can be found in the article/Supplementary Material.
