# Supplementary material for: A novel necroptosis-related lncRNA signature for predicting prognosis and immune response of colon cancer
Source: Front Genet. 2022 Aug 25;13:984696. doi: 10.3389/fgene.2022.984696 (PMC9453677; doi:10.3389/fgene.2022.984696)
Supplement: Supplementary file 1 [file Table1.DOCX]

| **Table S 1. Differentially expressed Necroptosis-Related genes** | | | | | |
| --- | --- | --- | --- | --- | --- |
| **Gene** | **Con Mean** | **Treat Mean** | **Log FC** | ***P* Value** | **fdr** |
| MYCN | 0.301557944 | 1.800678445 | 2.57803343 | 1.10E-07 | 2.35E-07 |
| FAS | 9.642523537 | 4.791014102 | -1.009079703 | 8.98E-15 | 3.66E-14 |
| MAPK10 | 0.593953427 | 0.179841618 | -1.723622893 | 1.03E-21 | 2.60E-20 |
| PLA2G4E | 0.037641913 | 0.129689737 | 1.784652442 | 3.45E-10 | 9.65E-10 |
| HSP90AB1 | 232.6771805 | 541.2067242 | 1.217850037 | 6.22E-23 | 3.13E-21 |
| CAMK2A | 0.190651367 | 0.078544536 | -1.279354058 | 1.01E-15 | 4.61E-15 |
| SMPD1 | 33.13662878 | 10.36944116 | -1.67608869 | 7.78E-25 | 1.17E-22 |
| PLK1 | 5.520468427 | 15.88501734 | 1.524804071 | 5.28E-20 | 5.69E-19 |
| PLA2G4D | 0.076290164 | 0.189826206 | 1.315110206 | 0.016866691 | 0.021048515 |
| DNMT1 | 5.750318024 | 11.89434629 | 1.048562331 | 1.15E-17 | 6.70E-17 |
| BACH2 | 0.414281046 | 0.194767164 | -1.088859341 | 5.82E-11 | 1.76E-10 |
| TNFSF10 | 51.54920098 | 20.34345669 | -1.341385228 | 3.35E-19 | 2.53E-18 |
| MYC | 25.65659254 | 104.3415262 | 2.023911958 | 1.26E-22 | 4.76E-21 |
| PLA2G4B | 0.046687821 | 0.120026735 | 1.362237622 | 6.51E-09 | 1.51E-08 |
| TLR3 | 5.552192122 | 1.531025947 | -1.858558757 | 5.00E-23 | 3.13E-21 |
| LEF1 | 0.653312402 | 3.154785378 | 2.271696928 | 1.72E-19 | 1.36E-18 |
| CAPN2 | 57.00540902 | 27.99029344 | -1.026172206 | 6.83E-19 | 4.48E-18 |
| TNFRSF10B | 7.268095488 | 19.50680486 | 1.424328211 | 1.89E-21 | 4.07E-20 |
| IL1A | 0.164439759 | 1.575973957 | 3.260612626 | 2.22E-15 | 9.86E-15 |
| FLT3 | 0.380681672 | 0.138017187 | -1.46373718 | 4.23E-19 | 3.04E-18 |
| TNFRSF10A | 3.930626234 | 8.181618961 | 1.057627165 | 1.70E-14 | 6.59E-14 |
| IFNG | 0.177614516 | 0.377865536 | 1.089123448 | 0.00162813 | 0.002319318 |
| IL33 | 6.76286522 | 14.84422861 | 1.134195617 | 0.029404356 | 0.035806917 |
| RBCK1 | 22.43514268 | 45.08140183 | 1.006772018 | 8.42E-17 | 4.39E-16 |
| TERT | 0.186701483 | 0.619781641 | 1.731026636 | 7.00E-09 | 1.60E-08 |
| PYGM | 2.739114554 | 0.232051431 | -3.561193106 | 5.11E-22 | 1.54E-20 |
| PGAM5 | 12.23280905 | 26.85005247 | 1.134169176 | 6.80E-20 | 6.84E-19 |
| CDKN2A | 0.290097522 | 2.377338483 | 3.03473745 | 2.21E-16 | 1.11E-15 |
| TRAF5 | 2.236977888 | 7.242145993 | 1.694866265 | 1.01E-20 | 1.88E-19 |
| KLF9 | 14.81042749 | 4.863828216 | -1.606449102 | 1.26E-20 | 1.90E-19 |
| IL1B | 2.997719668 | 10.4113528 | 1.796220157 | 4.84E-07 | 9.14E-07 |
| BCL2 | 3.531334976 | 1.326759366 | -1.412306946 | 3.85E-20 | 4.47E-19 |
| BID | 6.468850732 | 14.05085132 | 1.119076216 | 3.01E-20 | 3.79E-19 |
| ALK | 0.112167954 | 0.043298566 | -1.373269401 | 5.69E-19 | 3.91E-18 |
| HDAC9 | 0.83029281 | 0.313601238 | -1.404688952 | 8.24E-20 | 7.78E-19 |
