# Supplementary material for: A novel necroptosis-related lncRNA signature for predicting prognosis and immune response of colon cancer
Source: Front Genet. 2022 Aug 25;13:984696. doi: 10.3389/fgene.2022.984696 (PMC9453677; doi:10.3389/fgene.2022.984696)
Supplement: Supplementary file 2 [file Table2.DOCX]

| **Table S 2. Differentially expressed lncRNAs** | | | | | |
| --- | --- | --- | --- | --- | --- |
| **Gene** | **Con Mean** | **Treat Mean** | **Log FC** | ***P* Value** | **fdr** |
| AC005534.1 | 0.047118197 | 0.285672096 | 2.600003876 | 9.81E-14 | 7.45E-13 |
| AC091729.2 | 0.036188092 | 0.129880969 | 1.843603115 | 0.00493214 | 0.00742544 |
| LINC01819 | 0.246543804 | 3.540144365 | 3.84389229 | 0.00048036 | 0.00085601 |
| PRRT3-AS1 | 1.516317647 | 4.027782587 | 1.409413802 | 8.01E-13 | 5.42E-12 |
| AL138963.1 | 0.093276625 | 0.995169661 | 3.415355007 | 0.0002238 | 0.00041779 |
| AC005264.1 | 0.004021773 | 0.057296797 | 3.832550791 | 2.02E-14 | 1.69E-13 |
| PRMT5-AS1 | 0.046523586 | 0.162573081 | 1.805054193 | 5.97E-15 | 5.46E-14 |
| AC007272.1 | 0.015191399 | 0.048856583 | 1.685298239 | 0.00319012 | 0.0049617 |
| AC015921.1 | 0.011540365 | 0.052188482 | 2.177042634 | 0.016102 | 0.02213924 |
| AC006333.2 | 5.475964448 | 2.58045844 | -1.085485685 | 1.73E-19 | 3.65E-18 |
| LINC01152 | 0.031866599 | 0.09352054 | 1.553238224 | 0.00482727 | 0.00727761 |
| AC092198.1 | 0.012469087 | 0.09179922 | 2.880126063 | 2.80E-08 | 9.39E-08 |
| AC097381.3 | 0.044475575 | 0.151179885 | 1.765181033 | 0.00099428 | 0.00168223 |
| AC013476.1 | 0.043887208 | 0.255146321 | 2.539452427 | 3.22E-05 | 6.84E-05 |
| AC010320.3 | 0.079850428 | 0.298432474 | 1.902032479 | 0.00057164 | 0.00100587 |
| AL359878.2 | 0.025361936 | 0.107124907 | 2.078557145 | 0.00023155 | 0.00043111 |
| AC020907.4 | 0.280832519 | 1.222442186 | 2.12198433 | 2.34E-14 | 1.94E-13 |
| AC068790.4 | 0.108881827 | 0.438865137 | 2.011014489 | 0.01075778 | 0.01524089 |
| AC024941.1 | 0.124334955 | 0.048993775 | -1.343561591 | 1.45E-09 | 5.86E-09 |
| AC022387.1 | 0.01052961 | 0.20893822 | 4.310552468 | 8.26E-13 | 5.55E-12 |
| AC120036.4 | 0.106150667 | 0.043987562 | -1.270945875 | 1.52E-13 | 1.12E-12 |
| AC007182.1 | 1.933909654 | 0.094999641 | -4.347454527 | 2.44E-25 | 2.61E-23 |
| OGFR-AS1 | 0.104924054 | 0.241782914 | 1.204366837 | 3.23E-05 | 6.86E-05 |
| AC005498.1 | 0.042061628 | 0.016630607 | -1.338663866 | 1.49E-08 | 5.25E-08 |
| LINC01991 | 0.054682404 | 0.010519512 | -2.378008944 | 3.09E-11 | 1.62E-10 |
| AL356270.1 | 0.701888199 | 0.160152829 | -2.131791963 | 8.52E-15 | 7.64E-14 |
| AF064858.3 | 0.422575559 | 5.280374224 | 3.643358946 | 1.04E-06 | 2.80E-06 |
| AC092376.1 | 0.0624901 | 0.015705083 | -1.99239607 | 8.20E-16 | 8.75E-15 |
| AL662860.1 | 0.473077455 | 0.058018388 | -3.027494294 | 1.19E-25 | 1.44E-23 |
| AC008115.3 | 0.732665753 | 1.978918829 | 1.433485352 | 3.27E-10 | 1.47E-09 |
| AC137723.1 | 0.038610541 | 0.14999853 | 1.957881677 | 5.30E-05 | 0.00010911 |
| AP003548.1 | 0.119314961 | 0.033602953 | -1.828115036 | 1.54E-16 | 1.85E-15 |
| Z68871.1 | 0.223311366 | 0.648389503 | 1.537804052 | 1.43E-08 | 5.06E-08 |
| LINC01511 | 0.003979961 | 0.163565687 | 5.360971876 | 3.82E-11 | 1.99E-10 |
| AC079612.1 | 0.000603927 | 0.014159053 | 4.551207865 | 1.40E-09 | 5.69E-09 |
| FAM157C | 0.0241194 | 0.06796633 | 1.494626184 | 3.41E-08 | 1.13E-07 |
| AL162413.1 | 0.009114234 | 3.890880931 | 8.737759686 | 2.40E-10 | 1.11E-09 |
| LINC00622 | 0.116281064 | 0.289054649 | 1.313726096 | 0.00135711 | 0.00224456 |
| AC010524.1 | 0.040682784 | 0.220210358 | 2.436392023 | 0.03216557 | 0.04194138 |
| AL591895.1 | 9.17092528 | 18.61659428 | 1.021449966 | 2.69E-07 | 7.86E-07 |
| AC138819.1 | 0.107910868 | 0.025983839 | -2.054153684 | 1.18E-23 | 7.26E-22 |
| AL139260.2 | 0.030364043 | 0.012863545 | -1.239075619 | 1.37E-08 | 4.83E-08 |
| IGFBP7-AS1 | 0.033579377 | 0.269801257 | 3.006249693 | 0.00040869 | 0.00073575 |
| AL109811.1 | 0.038161605 | 0.090337789 | 1.24320777 | 5.11E-08 | 1.66E-07 |
| THUMPD3-AS1 | 1.211711917 | 2.593217838 | 1.097696662 | 6.45E-11 | 3.23E-10 |
| AC006449.3 | 0.047386172 | 0.149486954 | 1.657481559 | 2.65E-05 | 5.72E-05 |
| MINCR | 1.479262197 | 3.98144097 | 1.428412877 | 1.14E-16 | 1.41E-15 |
| LYPLAL1-DT | 0.141010405 | 0.051047677 | -1.465884401 | 9.77E-07 | 2.63E-06 |
| LINC00628 | 0.130803018 | 0.337025518 | 1.365462002 | 7.30E-11 | 3.64E-10 |
| LMCD1-AS1 | 0.021084585 | 0.077642478 | 1.880657512 | 1.74E-14 | 1.48E-13 |
| AL110115.2 | 0.081877226 | 0.176867365 | 1.111133731 | 0.00561286 | 0.00837628 |
| AC016708.1 | 0.032134865 | 0.104580611 | 1.702404068 | 0.00201184 | 0.00323615 |
| AC073195.1 | 0.234288209 | 0.478750895 | 1.030990835 | 1.02E-08 | 3.66E-08 |
| SKAP1-AS1 | 0.020743542 | 0.131259885 | 2.661691884 | 0.00040439 | 0.00072874 |
| PRR7-AS1 | 0.094701227 | 0.619933341 | 2.710658077 | 1.11E-21 | 3.99E-20 |
| AC058791.1 | 0.172828096 | 1.299617 | 2.910676843 | 8.58E-05 | 0.00017072 |
| C9orf41-AS1 | 0.092401758 | 0.375230503 | 2.021784907 | 0.00668527 | 0.00982344 |
| AC020571.1 | 0.486373997 | 0.226906097 | -1.099970724 | 2.49E-10 | 1.14E-09 |
| AP000842.3 | 0.010717728 | 0.053059001 | 2.307598432 | 0.03011334 | 0.03947309 |
| AC004156.1 | 0.019210761 | 0.040990481 | 1.093374233 | 0.00462395 | 0.007004 |
| LINC00671 | 0.038357038 | 0.018211685 | -1.074626904 | 8.80E-09 | 3.19E-08 |
| AC022784.1 | 0.016516884 | 0.388252925 | 4.55498334 | 1.54E-15 | 1.57E-14 |
| AC138230.1 | 0.155236397 | 0.364481202 | 1.23137756 | 8.19E-10 | 3.47E-09 |
| AL121929.1 | 0.1384367 | 0.033816669 | -2.033419992 | 3.00E-17 | 4.07E-16 |
| AC018553.1 | 0.01682507 | 0.218481183 | 3.698824655 | 0.00037938 | 0.00068683 |
| ZFAS1 | 17.45694412 | 65.61206089 | 1.910160003 | 2.19E-23 | 1.27E-21 |
| AC079907.1 | 0.233733942 | 0.586342812 | 1.326877147 | 5.86E-10 | 2.55E-09 |
| MIR3142HG | 0.492464789 | 1.740727844 | 1.821598179 | 9.75E-09 | 3.51E-08 |
| UBOX5-AS1 | 0.077858293 | 0.216030209 | 1.472310458 | 4.17E-06 | 1.03E-05 |
| AC087277.2 | 1.810730526 | 0.782437464 | -1.210524505 | 1.11E-13 | 8.36E-13 |
| AL954642.1 | 0.184775803 | 0.035682841 | -2.372473455 | 1.90E-15 | 1.90E-14 |
| BVES-AS1 | 0.198445308 | 0.026994956 | -2.877979678 | 9.94E-21 | 2.82E-19 |
| AC074050.4 | 0.049392981 | 0.209687706 | 2.085864339 | 2.33E-06 | 5.96E-06 |
| AC012640.3 | 0.445671598 | 0.161611675 | -1.463449603 | 1.63E-12 | 1.04E-11 |
| AC003101.2 | 0.027476474 | 0.219122175 | 2.995466689 | 3.58E-10 | 1.60E-09 |
| AL161668.4 | 0.411601852 | 0.169490393 | -1.280045978 | 1.36E-12 | 8.82E-12 |
| LINC00880 | 0.031275204 | 0.090218764 | 1.528408231 | 2.75E-11 | 1.45E-10 |
| AC025580.1 | 20.00197533 | 6.760314609 | -1.56498019 | 1.10E-16 | 1.36E-15 |
| LINC01703 | 0.278420177 | 0.955117944 | 1.778415134 | 1.01E-14 | 8.94E-14 |
| LINC01797 | 0.114761985 | 0.037360216 | -1.619070113 | 3.08E-16 | 3.52E-15 |
| AL590729.1 | 0.069084144 | 0.186660364 | 1.43398909 | 0.01180882 | 0.01664345 |
| IGFL2-AS1 | 0.008380455 | 0.700589084 | 6.38539604 | 8.55E-15 | 7.66E-14 |
| AC245884.8 | 0.373432346 | 1.187237425 | 1.668689675 | 6.34E-12 | 3.69E-11 |
| AC090206.1 | 0.010331981 | 0.004156488 | -1.31367986 | 8.72E-12 | 4.96E-11 |
| AP000704.1 | 0.171613035 | 0.37047635 | 1.110222308 | 1.23E-07 | 3.78E-07 |
| AC009097.4 | 0.265968997 | 0.07006958 | -1.924397944 | 8.67E-13 | 5.82E-12 |
| EIF1AX-AS1 | 0.030596916 | 0.560459525 | 4.19515206 | 0.00640103 | 0.00945674 |
| LINC01975 | 0.480929022 | 0.087021221 | -2.466384832 | 1.71E-21 | 5.83E-20 |
| LINC01215 | 0.363420541 | 0.151859719 | -1.258900722 | 1.88E-08 | 6.47E-08 |
| MLIP-AS1 | 0.011856996 | 0.003054795 | -1.956591062 | 1.54E-35 | 8.40E-33 |
| AC068658.1 | 0.074041931 | 0.26095913 | 1.817409447 | 0.01332715 | 0.01862454 |
| AC099518.4 | 0.141945972 | 0.296714316 | 1.063732633 | 4.54E-06 | 1.11E-05 |
| LIFR-AS1 | 0.099590196 | 0.025607954 | -1.959411749 | 9.71E-18 | 1.44E-16 |
| LINC01711 | 0.014508854 | 0.182912179 | 3.656145703 | 3.34E-14 | 2.71E-13 |
| AC243571.2 | 0.021960987 | 0.056382085 | 1.360293952 | 0.01739856 | 0.02379592 |
| AC092111.1 | 0.103293008 | 0.049461503 | -1.062364598 | 2.09E-10 | 9.73E-10 |
| AL445363.1 | 0.072501179 | 0.012759872 | -2.506390558 | 1.45E-22 | 6.47E-21 |
| AP000692.1 | 0.208111953 | 0.553746328 | 1.411865395 | 1.07E-05 | 2.46E-05 |
| LINC01977 | 0.028203166 | 0.418608822 | 3.891673688 | 1.19E-21 | 4.17E-20 |
| AC025162.2 | 0.068361375 | 0.143768739 | 1.072496685 | 0.00880369 | 0.01265661 |
| LINC02000 | 0.749409897 | 0.063319463 | -3.565034106 | 4.60E-26 | 6.12E-24 |
| AL137060.1 | 0.043155194 | 0.17614355 | 2.029145519 | 1.71E-17 | 2.40E-16 |
| LINC01943 | 0.16312563 | 0.430380628 | 1.399629668 | 1.13E-09 | 4.67E-09 |
| AC124283.3 | 0.084257955 | 0.399389293 | 2.244910845 | 0.00491108 | 0.00739987 |
| AC137932.3 | 0.186499506 | 0.480904123 | 1.366577487 | 2.11E-11 | 1.13E-10 |
| AF186192.1 | 0.328739393 | 0.105725895 | -1.63661557 | 2.55E-17 | 3.50E-16 |
| HM13-IT1 | 0.531299624 | 1.574333967 | 1.567144019 | 1.57E-11 | 8.65E-11 |
| AC009065.2 | 7.809713732 | 16.98904158 | 1.121262894 | 3.49E-14 | 2.82E-13 |
| AL031595.3 | 0.015790284 | 0.077377002 | 2.292867688 | 8.02E-05 | 0.00015988 |
| AC078852.2 | 0.027534789 | 0.122804149 | 2.157031833 | 0.00049344 | 0.00087674 |
| RXYLT1-AS1 | 0.095757258 | 0.044244485 | -1.113884206 | 0.00013982 | 0.00026866 |
| AC078852.1 | 0.028038519 | 0.197572001 | 2.816896458 | 0.00057452 | 0.00100929 |
| AP006284.1 | 0.25507 | 0.812365305 | 1.671235398 | 1.62E-09 | 6.52E-09 |
| SNHG22 | 0.175241636 | 0.824491645 | 2.234159284 | 6.89E-07 | 1.91E-06 |
| AC068724.1 | 0.045556853 | 0.139961009 | 1.619284957 | 6.11E-05 | 0.00012461 |
| NEAT1 | 10.80814554 | 37.89709552 | 1.809968276 | 0.00595579 | 0.00885412 |
| AP000763.3 | 0.056603771 | 0.17024559 | 1.588647361 | 0.01602886 | 0.02205535 |
| AF127577.3 | 0.01902211 | 0.543655436 | 4.836943421 | 1.88E-07 | 5.65E-07 |
| AC099518.2 | 0.305339508 | 0.650807364 | 1.091816301 | 7.54E-06 | 1.78E-05 |
| AC233728.1 | 0.258078943 | 0.689399479 | 1.417527773 | 1.45E-08 | 5.12E-08 |
| AC139491.2 | 0.209318179 | 0.098270795 | -1.09086299 | 1.03E-12 | 6.82E-12 |
| CRNDE | 0.070197062 | 2.168626214 | 4.949226947 | 1.12E-24 | 9.68E-23 |
| AC133785.1 | 0.001827857 | 0.047969396 | 4.713889368 | 2.05E-10 | 9.55E-10 |
| AL512625.2 | 0.055273963 | 0.135328676 | 1.291795605 | 0.03354395 | 0.04362393 |
| AL136038.5 | 0.127203142 | 0.366132468 | 1.525231409 | 6.10E-10 | 2.64E-09 |
| LINC01615 | 0.0260025 | 0.30575511 | 3.555654362 | 4.93E-17 | 6.44E-16 |
| LINC01559 | 20.36011954 | 9.587545227 | -1.086512648 | 7.98E-09 | 2.92E-08 |
| AC103591.3 | 0.349667057 | 1.64368039 | 2.232876008 | 5.80E-07 | 1.62E-06 |
| LINC01929 | 0.009123221 | 0.108835854 | 3.576466865 | 7.93E-12 | 4.54E-11 |
| AC079145.1 | 0.050714391 | 0.299778754 | 2.563431039 | 8.73E-11 | 4.30E-10 |
| AC009121.1 | 0.080436879 | 0.292392458 | 1.861977096 | 2.97E-12 | 1.82E-11 |
| AL008723.3 | 0.025064412 | 0.235882245 | 3.234354546 | 6.01E-06 | 1.44E-05 |
| AC092718.6 | 0.023684331 | 0.192890318 | 3.025775874 | 0.00015019 | 0.00028756 |
| AC009120.5 | 0.023346062 | 0.080966134 | 1.794139369 | 2.27E-05 | 4.99E-05 |
| LINC02206 | 0.041123179 | 0.011234287 | -1.872043272 | 1.46E-12 | 9.43E-12 |
| AP001207.3 | 0.033860257 | 0.155420992 | 2.198516544 | 0.00012239 | 0.00023708 |
| Z82217.1 | 0.005927526 | 0.132583705 | 4.483329694 | 5.41E-06 | 1.31E-05 |
| GK-AS1 | 0.247653693 | 1.103527809 | 2.155726949 | 0.00018223 | 0.00034397 |
| LINC00240 | 0.022287089 | 0.052101476 | 1.225116029 | 3.78E-05 | 7.96E-05 |
| AC008758.2 | 0.053540288 | 0.169409065 | 1.661814285 | 0.00051579 | 0.00091288 |
| MELTF-AS1 | 0.553658012 | 2.315288552 | 2.064124985 | 7.83E-22 | 2.90E-20 |
| AC037487.1 | 0.017745955 | 0.113501288 | 2.677146571 | 0.00025514 | 0.00047242 |
| LINC02381 | 1.984890359 | 0.777902694 | -1.351397709 | 2.71E-18 | 4.41E-17 |
| ARHGEF38-IT1 | 0.094761579 | 0.737797871 | 2.960851485 | 2.71E-09 | 1.06E-08 |
| AP000695.2 | 0.029577662 | 0.217505876 | 2.878474454 | 2.84E-16 | 3.26E-15 |
| AC106886.2 | 0.16515545 | 0.420013763 | 1.346612022 | 1.48E-08 | 5.20E-08 |
| AC006007.1 | 0.118464536 | 0.012258403 | -3.272612265 | 1.51E-30 | 4.13E-28 |
| AC022893.1 | 0.139233051 | 0.291105791 | 1.06404182 | 0.00736094 | 0.01073815 |
| DIRC3 | 0.133061416 | 0.040761502 | -1.706813168 | 1.65E-17 | 2.33E-16 |
| AC131159.1 | 0.157580063 | 0.355865056 | 1.175245258 | 0.03697336 | 0.04773328 |
| AC015908.2 | 1.080344382 | 0.272699318 | -1.986108278 | 1.89E-20 | 5.02E-19 |
| AC007405.1 | 0.024403089 | 0.108326709 | 2.150253291 | 1.85E-12 | 1.17E-11 |
| AC011290.1 | 0.034098774 | 0.13250254 | 1.958228242 | 4.29E-05 | 8.97E-05 |
| AL022311.1 | 0.070769433 | 0.174230831 | 1.299801677 | 3.67E-15 | 3.49E-14 |
| AC008494.3 | 0.806019487 | 0.283568984 | -1.507114974 | 1.21E-21 | 4.20E-20 |
| AC087392.4 | 0.10253152 | 0.213597035 | 1.05882413 | 0.02378094 | 0.03176034 |
| U47924.3 | 0.099302864 | 0.245028604 | 1.303042943 | 1.62E-05 | 3.64E-05 |
| AC137056.1 | 0.024488172 | 0.002550946 | -3.262980751 | 2.02E-38 | 1.85E-35 |
| AC073487.1 | 0.216864903 | 0.645973246 | 1.574677825 | 1.90E-05 | 4.21E-05 |
| AC132872.3 | 0.886231786 | 1.843655568 | 1.056813179 | 2.36E-07 | 6.98E-07 |
| AC125807.2 | 0.670938787 | 1.645850488 | 1.29458023 | 4.10E-16 | 4.61E-15 |
| PLAC4 | 0.010983289 | 0.743974847 | 6.081871809 | 6.19E-19 | 1.14E-17 |
| LINC01630 | 0.001150128 | 0.034881857 | 4.922609925 | 2.65E-05 | 5.72E-05 |
| SCARNA9 | 0.469788005 | 7.090874825 | 3.915881847 | 2.97E-12 | 1.82E-11 |
| AL928654.2 | 1.154732157 | 2.586885379 | 1.163657878 | 2.48E-07 | 7.27E-07 |
| AP001269.2 | 0.069943346 | 0.02842118 | -1.299220362 | 1.95E-08 | 6.71E-08 |
| AL357054.4 | 1.283548518 | 0.248747882 | -2.367381686 | 1.04E-22 | 4.84E-21 |
| AP002336.2 | 0.165196915 | 0.570147153 | 1.787147576 | 1.18E-10 | 5.69E-10 |
| AC009509.1 | 1.900722191 | 0.58684874 | -1.695487081 | 2.26E-16 | 2.65E-15 |
| AC005306.1 | 0.113989535 | 0.231285372 | 1.020772642 | 1.74E-05 | 3.88E-05 |
| LINC01489 | 0.075401254 | 0.026181851 | -1.52602141 | 2.03E-05 | 4.48E-05 |
| AC089983.1 | 0.015288485 | 0.064549306 | 2.077956136 | 0.00103374 | 0.00174304 |
| AP006621.1 | 0.146348807 | 0.474890795 | 1.698184806 | 0.00040712 | 0.00073316 |
| DARS-AS1 | 0.072020485 | 0.170523369 | 1.243490249 | 4.75E-09 | 1.79E-08 |
| AC004241.4 | 0.031227534 | 0.137426289 | 2.137767452 | 2.54E-06 | 6.47E-06 |
| CD44-AS1 | 0.170170976 | 1.195230691 | 2.812232201 | 1.37E-13 | 1.01E-12 |
| AC005540.1 | 0.104288051 | 0.322561434 | 1.629000088 | 0.00025317 | 0.00046893 |
| AC083880.1 | 0.299679225 | 0.812551683 | 1.439040506 | 2.34E-13 | 1.68E-12 |
| AC092535.4 | 0.763944344 | 2.277368375 | 1.575828232 | 7.23E-12 | 4.17E-11 |
| AL022724.1 | 0.076596654 | 0.026766368 | -1.51685998 | 1.47E-09 | 5.96E-09 |
| AC016722.2 | 0.086537701 | 0.211853245 | 1.291664533 | 1.28E-05 | 2.90E-05 |
| AC005391.1 | 0.05471254 | 0.208972746 | 1.93337136 | 0.0020947 | 0.00336151 |
| AC027796.4 | 0.41092594 | 1.018038631 | 1.308841998 | 1.39E-09 | 5.69E-09 |
| FOCAD-AS1 | 0.083750917 | 0.300630343 | 1.843813734 | 3.03E-07 | 8.80E-07 |
| AC108063.1 | 0.029265208 | 0.199714724 | 2.770682275 | 0.0229771 | 0.03076212 |
| ZNF346-IT1 | 0.138474954 | 0.384595525 | 1.473716915 | 5.55E-05 | 0.00011368 |
| AL139384.1 | 0.221771519 | 0.650941518 | 1.553453834 | 4.39E-09 | 1.66E-08 |
| AC092118.2 | 0.046790537 | 0.13539874 | 1.53292561 | 1.98E-05 | 4.37E-05 |
| AC010280.1 | 0.16387813 | 0.847262462 | 2.370185616 | 0.00095418 | 0.00161739 |
| CYP1B1-AS1 | 0.148614829 | 0.056544675 | -1.394114996 | 2.88E-19 | 5.74E-18 |
| AC021678.2 | 0.05708861 | 0.011739519 | -2.281829595 | 1.50E-14 | 1.29E-13 |
| AC023983.2 | 0.036530242 | 0.095109431 | 1.380497077 | 0.03032201 | 0.03971797 |
| LINC01572 | 0.049810991 | 0.213040669 | 2.096592847 | 2.03E-17 | 2.84E-16 |
| AC006504.1 | 0.082237608 | 0.17817618 | 1.115434267 | 0.0011014 | 0.00184912 |
| AC055717.2 | 0.002987512 | 1.504354545 | 8.975984632 | 5.92E-17 | 7.71E-16 |
| AC104024.1 | 0.13826914 | 0.028487788 | -2.279063712 | 8.70E-19 | 1.52E-17 |
| MAFG-DT | 0.374724752 | 3.314834525 | 3.145033672 | 7.86E-25 | 6.92E-23 |
| AL033397.1 | 0.010034613 | 0.143218014 | 3.835156024 | 0.00527717 | 0.00790343 |
| AC008764.6 | 0.237296799 | 0.62683382 | 1.401390381 | 9.13E-16 | 9.69E-15 |
| LINC01844 | 0.006520679 | 0.036904554 | 2.500704638 | 0.00016077 | 0.00030621 |
| AL513327.2 | 0.057560264 | 0.175171741 | 1.605624936 | 0.01118708 | 0.01582031 |
| LINC02538 | 0.066401053 | 0.16467154 | 1.31031322 | 0.00029748 | 0.00054618 |
| VPS13A-AS1 | 0.043566816 | 0.019655656 | -1.148285127 | 5.60E-07 | 1.57E-06 |
| AC009093.1 | 0.03843721 | 0.350857438 | 3.190309417 | 5.11E-13 | 3.56E-12 |
| AC025176.1 | 0.121859743 | 0.486416401 | 1.996970268 | 1.76E-09 | 7.06E-09 |
| AL133297.1 | 0.084310634 | 0.212120381 | 1.331096741 | 7.97E-05 | 0.00015907 |
| AP000873.2 | 0.269880459 | 0.570139667 | 1.078994855 | 3.34E-06 | 8.33E-06 |
| AL023803.3 | 0.025499823 | 0.17510805 | 2.779686243 | 3.80E-09 | 1.46E-08 |
| AC130456.3 | 0.453091477 | 1.006056629 | 1.150837257 | 1.89E-05 | 4.19E-05 |
| AC083806.2 | 0.013431246 | 0.119295137 | 3.15087019 | 1.49E-05 | 3.36E-05 |
| AL138995.1 | 0.664024323 | 0.169363549 | -1.971112684 | 1.80E-21 | 6.08E-20 |
| AC129507.2 | 1.680512939 | 0.761998924 | -1.141040785 | 1.37E-12 | 8.91E-12 |
| AP001160.4 | 0.179206838 | 0.453347777 | 1.33899252 | 0.00260275 | 0.00411385 |
| LINC00858 | 0.007932275 | 0.619104818 | 6.286305137 | 9.99E-17 | 1.24E-15 |
| LINC01303 | 0.005448753 | 0.044997479 | 3.045846082 | 2.94E-10 | 1.34E-09 |
| GLYCTK-AS1 | 0.054839997 | 0.155480162 | 1.503430103 | 0.02860512 | 0.03764995 |
| AC012558.1 | 0.063480162 | 0.029508006 | -1.105199397 | 4.00E-13 | 2.81E-12 |
| AC012615.4 | 0.119552873 | 0.342258876 | 1.517439152 | 8.26E-05 | 0.0001647 |
| AC004585.1 | 0.295266299 | 0.779820518 | 1.401125414 | 2.06E-12 | 1.29E-11 |
| NALT1 | 1.341697598 | 0.640664092 | -1.066419505 | 0.00413732 | 0.00631248 |
| AC119428.2 | 0.087603204 | 0.036641182 | -1.257517595 | 1.47E-09 | 5.95E-09 |
| LINC01811 | 0.005606981 | 0.828703432 | 7.207487818 | 2.40E-19 | 4.82E-18 |
| LINC02373 | 0.026274627 | 0.068430982 | 1.380979402 | 0.0250424 | 0.03324963 |
| ZDHHC20-IT1 | 0.117845596 | 0.575148554 | 2.287036785 | 2.33E-10 | 1.08E-09 |
| EXTL3-AS1 | 0.021159946 | 0.113170448 | 2.419089426 | 2.63E-14 | 2.17E-13 |
| AC126365.1 | 0.006378382 | 0.026762162 | 2.068932205 | 2.84E-05 | 6.08E-05 |
| AP001610.2 | 2.199641058 | 0.876898517 | -1.326786325 | 5.63E-11 | 2.85E-10 |
| LINC01754 | 0.108246384 | 0.021993064 | -2.299198328 | 1.87E-18 | 3.13E-17 |
| LINC00484 | 0.260052274 | 0.075464536 | -1.784930933 | 6.12E-21 | 1.85E-19 |
| AL391807.1 | 0.084788869 | 0.016566813 | -2.355578826 | 2.45E-22 | 1.06E-20 |
| C17orf82 | 0.065284165 | 0.221715544 | 1.763904914 | 1.07E-07 | 3.32E-07 |
| AP003555.2 | 0.145427852 | 0.475353041 | 1.708695792 | 0.00024853 | 0.00046082 |
| AC245060.5 | 0.20891019 | 0.461583253 | 1.143708017 | 6.02E-05 | 0.00012285 |
| LINC01968 | 0.003507274 | 0.014937663 | 2.090532332 | 3.35E-06 | 8.35E-06 |
| LINC01352 | 0.21119611 | 0.0601068 | -1.812983137 | 7.67E-18 | 1.16E-16 |
| LINC00885 | 0.008656291 | 0.052580737 | 2.602713428 | 0.00017656 | 0.00033407 |
| AC010538.1 | 0.150389647 | 0.302084204 | 1.006245498 | 3.69E-06 | 9.15E-06 |
| AC002128.2 | 0.283037808 | 0.632220075 | 1.159432063 | 0.00273538 | 0.00430602 |
| LINC01050 | 0.001263795 | 0.323822744 | 8.001297934 | 4.45E-15 | 4.16E-14 |
| AC007993.2 | 0.082974835 | 0.013308883 | -2.640284359 | 7.84E-26 | 1.02E-23 |
| EIPR1-IT1 | 0.015609326 | 0.219385759 | 3.812989723 | 8.15E-10 | 3.45E-09 |
| AL135924.2 | 0.768605017 | 0.207768864 | -1.88726292 | 5.93E-11 | 2.99E-10 |
| AC004584.1 | 0.049569845 | 0.138098124 | 1.47815908 | 7.91E-05 | 0.00015811 |
| EPHA1-AS1 | 0.033281218 | 0.113036437 | 1.764007744 | 0.00648615 | 0.00957212 |
| AC025470.2 | 0.150565441 | 0.009885819 | -3.928886321 | 2.04E-38 | 1.85E-35 |
| AC104472.1 | 0.010465041 | 0.080609432 | 2.94537073 | 1.05E-09 | 4.36E-09 |
| AC078778.2 | 0.09811979 | 0.307729428 | 1.649046364 | 0.00411921 | 0.00629012 |
| LEMD1-AS1 | 0.003747868 | 0.064980659 | 4.115868356 | 7.22E-10 | 3.09E-09 |
| AC116025.1 | 0.024839045 | 0.128834409 | 2.37483632 | 0.00160145 | 0.0026248 |
| AC125616.1 | 0.017165298 | 0.193221953 | 3.492692208 | 3.41E-11 | 1.79E-10 |
| AC112721.1 | 0.011362434 | 0.132473575 | 3.543360818 | 1.92E-07 | 5.74E-07 |
| AL391987.4 | 0.212022047 | 0.058442594 | -1.859122157 | 1.49E-12 | 9.60E-12 |
| AL354836.1 | 0.917089185 | 2.844912498 | 1.633250334 | 4.37E-16 | 4.87E-15 |
| LINC01876 | 0.154416311 | 0.649919948 | 2.073436877 | 0.00022769 | 0.00042447 |
| LINC02427 | 0.098532398 | 0.035674515 | -1.465704374 | 1.33E-13 | 9.87E-13 |
| AC124798.1 | 1.806273306 | 4.772690105 | 1.401786462 | 1.40E-15 | 1.44E-14 |
| LINC02490 | 1.915118431 | 0.078766971 | -4.60369901 | 2.56E-24 | 1.99E-22 |
| AC011603.2 | 0.047074801 | 0.266723248 | 2.502316673 | 7.04E-13 | 4.80E-12 |
| AC004147.4 | 0.34034962 | 0.026580044 | -3.678602104 | 1.11E-35 | 6.75E-33 |
| AC244100.2 | 0.109886592 | 0.04251466 | -1.369983057 | 2.67E-16 | 3.10E-15 |
| AC092691.1 | 0.185632858 | 0.044598122 | -2.057397231 | 1.56E-36 | 1.06E-33 |
| LINC00461 | 0.053105002 | 0.013282775 | -1.999291134 | 2.06E-16 | 2.43E-15 |
| DLEU7-AS1 | 0.027908359 | 0.246616067 | 3.143497575 | 1.17E-14 | 1.01E-13 |
| DBET | 0.009183585 | 0.094784019 | 3.367514511 | 5.94E-05 | 0.00012123 |
| AC068594.1 | 0.267895867 | 0.118417333 | -1.177792063 | 1.43E-12 | 9.23E-12 |
| AL513217.1 | 0.406010017 | 0.050227506 | -3.01496578 | 1.36E-15 | 1.40E-14 |
| AL591806.1 | 0.007018455 | 0.073184665 | 3.382315956 | 9.91E-06 | 2.29E-05 |
| AC138150.2 | 0.07539443 | 0.338677005 | 2.167380181 | 7.89E-17 | 1.01E-15 |
| CLDN10-AS1 | 0.004925336 | 0.469605687 | 6.57508381 | 2.96E-12 | 1.82E-11 |
| AC087588.2 | 0.482507409 | 1.043755584 | 1.113160915 | 9.83E-05 | 0.0001936 |
| AP003071.3 | 0.23573343 | 0.060935894 | -1.951792166 | 8.54E-08 | 2.68E-07 |
| AC005220.1 | 0.050609894 | 0.013137525 | -1.945725961 | 1.11E-14 | 9.70E-14 |
| LINC02430 | 0.114869104 | 0.031587452 | -1.862567356 | 4.70E-20 | 1.14E-18 |
| AC010136.1 | 0.104443508 | 0.047068393 | -1.149892302 | 2.00E-12 | 1.25E-11 |
| AC006115.1 | 0.032742172 | 0.013644456 | -1.26283519 | 4.76E-08 | 1.55E-07 |
| AC104590.1 | 0.020803665 | 0.063654453 | 1.613423724 | 6.53E-05 | 0.00013248 |
| U73166.1 | 0.080544518 | 0.276878845 | 1.781396521 | 5.23E-12 | 3.09E-11 |
| LINC01730 | 0.054902322 | 0.535357572 | 3.285563748 | 2.92E-17 | 3.98E-16 |
| AC027808.2 | 0.028813689 | 0.280936197 | 3.285416247 | 4.90E-05 | 0.00010137 |
| AC104699.1 | 1.953052809 | 0.503826872 | -1.954730983 | 1.97E-15 | 1.97E-14 |
| IGBP1-AS2 | 0.027400889 | 0.166041738 | 2.599251352 | 3.66E-05 | 7.72E-05 |
| AC015849.3 | 0.962138806 | 2.216627774 | 1.204049578 | 4.33E-06 | 1.06E-05 |
| LINC01013 | 0.112251415 | 0.028633224 | -1.970971592 | 8.66E-21 | 2.54E-19 |
| HLX-AS1 | 0.110034166 | 0.028286532 | -1.959764319 | 2.45E-16 | 2.85E-15 |
| LINC01355 | 0.398741944 | 1.038255491 | 1.380634226 | 1.24E-07 | 3.80E-07 |
| AL357874.2 | 0.033939088 | 0.116727992 | 1.782130856 | 0.0140487 | 0.01955772 |
| AC009831.3 | 0.216695448 | 0.084991873 | -1.350272055 | 2.89E-14 | 2.36E-13 |
| LINC01232 | 0.332747542 | 1.173929208 | 1.818845499 | 1.17E-19 | 2.54E-18 |
| TM4SF1-AS1 | 0.107253333 | 0.407825394 | 1.926929136 | 2.66E-11 | 1.41E-10 |
| AL391427.1 | 0.042685088 | 0.156866182 | 1.877730307 | 0.00096512 | 0.00163391 |
| RB1-DT | 0.045729977 | 0.1023451 | 1.162229932 | 0.00023041 | 0.00042925 |
| AC008760.1 | 0.640216089 | 1.296732656 | 1.018250235 | 7.28E-09 | 2.68E-08 |
| AC112721.2 | 0.015907086 | 0.235017351 | 3.885025804 | 6.78E-09 | 2.51E-08 |
| AC005920.3 | 0.025783208 | 0.064088067 | 1.313623995 | 0.00037172 | 0.00067386 |
| AC006329.1 | 1.026904825 | 8.437372108 | 3.038491252 | 2.15E-18 | 3.55E-17 |
| AL353804.2 | 0.061110325 | 0.578397497 | 3.242573246 | 7.18E-06 | 1.70E-05 |
| FLJ21408 | 0.052040239 | 0.124828303 | 1.262245586 | 6.89E-08 | 2.19E-07 |
| AC026740.1 | 0.578595975 | 1.238539759 | 1.098011989 | 0.00019727 | 0.00037083 |
| AC099791.2 | 0.038014067 | 0.186124659 | 2.291663928 | 2.26E-08 | 7.72E-08 |
| AC006111.2 | 0.131169198 | 0.490557034 | 1.902991898 | 3.08E-15 | 2.97E-14 |
| AC005730.2 | 0.045831014 | 0.012710006 | -1.850359542 | 4.14E-14 | 3.31E-13 |
| AL354993.2 | 0.184346558 | 0.640689732 | 1.797205393 | 0.00140499 | 0.00231953 |
| AC022613.1 | 1.605569628 | 0.7345302 | -1.128191519 | 2.06E-11 | 1.11E-10 |
| AC034102.6 | 0.075992667 | 0.1821104 | 1.260881208 | 4.55E-06 | 1.11E-05 |
| AL451164.1 | 0.124402273 | 0.03831674 | -1.69896612 | 8.93E-21 | 2.60E-19 |
| AC021546.1 | 0.042374861 | 0.01168598 | -1.858429878 | 3.92E-21 | 1.24E-19 |
| AC078860.1 | 0.081216808 | 0.737533978 | 3.182859282 | 3.35E-12 | 2.04E-11 |
| AC243960.1 | 0.934610302 | 0.363239523 | -1.363443751 | 3.29E-14 | 2.68E-13 |
| AC110619.1 | 0.283650707 | 0.116261532 | -1.286741638 | 8.55E-11 | 4.22E-10 |
| FGF13-AS1 | 0.093876515 | 0.036329956 | -1.369604677 | 8.41E-12 | 4.80E-11 |
| AC132192.2 | 0.417676268 | 1.032964457 | 1.306333536 | 6.80E-12 | 3.94E-11 |
| AC116025.2 | 0.018528911 | 0.433118506 | 4.546911829 | 3.41E-19 | 6.69E-18 |
| AC092119.2 | 0.467779933 | 1.064957377 | 1.186893812 | 8.89E-08 | 2.78E-07 |
| ST7-OT4 | 0.010850935 | 0.219238321 | 4.336608755 | 1.00E-09 | 4.21E-09 |
| AL157931.1 | 0.281787484 | 0.041441833 | -2.765447809 | 5.41E-23 | 2.73E-21 |
| AC007497.1 | 0.046843691 | 0.241517937 | 2.366203685 | 3.17E-10 | 1.43E-09 |
| LINC01505 | 0.049379731 | 0.010599084 | -2.219979328 | 3.19E-21 | 1.03E-19 |
| AC021491.4 | 0.329437905 | 0.069039057 | -2.254521906 | 5.40E-20 | 1.28E-18 |
| MMP2-AS1 | 0.049431589 | 0.195805566 | 1.985916576 | 0.00028715 | 0.00052811 |
| PSPC1-AS2 | 0.392760566 | 0.956839235 | 1.284626462 | 1.74E-05 | 3.88E-05 |
| AC138904.1 | 0.075917784 | 0.388268408 | 2.354544543 | 4.13E-10 | 1.83E-09 |
| AL162582.1 | 0.023661703 | 1.951704936 | 6.366037262 | 2.83E-16 | 3.26E-15 |
| AL357033.1 | 0.167630176 | 0.475402139 | 1.503866514 | 7.38E-05 | 0.00014821 |
| BBOX1-AS1 | 0.071518552 | 2.35227102 | 5.039592953 | 4.90E-18 | 7.63E-17 |
| AC011611.2 | 0.017702086 | 0.288947099 | 4.028814104 | 1.32E-07 | 4.03E-07 |
| AL109614.1 | 0.294118271 | 1.384125422 | 2.234506361 | 4.25E-08 | 1.39E-07 |
| AC104984.2 | 0.027791253 | 0.211284652 | 2.926485178 | 0.00090268 | 0.00153679 |
| SLC16A1-AS1 | 0.115947558 | 0.470040004 | 2.019311112 | 1.23E-17 | 1.81E-16 |
| AL354813.1 | 0.016659256 | 0.104854962 | 2.653999297 | 3.88E-07 | 1.11E-06 |
| AL161891.1 | 0.13941869 | 0.56867748 | 2.028186695 | 4.35E-17 | 5.76E-16 |
| Z92544.2 | 0.27591008 | 0.621089147 | 1.170602193 | 4.49E-10 | 1.98E-09 |
| AC244093.5 | 0.061406617 | 0.248897536 | 2.019085913 | 0.00026025 | 0.00048107 |
| LINC00894 | 0.122194643 | 0.531668078 | 2.121344804 | 1.66E-11 | 9.07E-11 |
| AC013731.1 | 0.120609643 | 0.323076526 | 1.421530671 | 3.89E-09 | 1.48E-08 |
| AL078587.1 | 0.106765312 | 0.73105265 | 2.775532325 | 5.55E-11 | 2.81E-10 |
| AC012360.1 | 0.030284832 | 0.125281736 | 2.048508786 | 1.73E-06 | 4.50E-06 |
| AL391845.2 | 0.005033621 | 0.189069857 | 5.231179088 | 4.26E-22 | 1.72E-20 |
| MAP3K20-AS1 | 0.058355428 | 1.186294236 | 4.345451219 | 1.32E-17 | 1.92E-16 |
| AC004832.5 | 0.098146014 | 0.454368705 | 2.210861884 | 1.58E-05 | 3.55E-05 |
| TRPM2-AS | 0.214500788 | 2.163410933 | 3.334252875 | 1.30E-14 | 1.12E-13 |
| AC020765.2 | 0.423678094 | 0.885194815 | 1.063026461 | 4.76E-07 | 1.35E-06 |
| UCKL1-AS1 | 0.143348809 | 0.482359402 | 1.750578568 | 5.46E-10 | 2.39E-09 |
| HHATL-AS1 | 0.050645017 | 0.009501837 | -2.414141928 | 6.07E-20 | 1.42E-18 |
| AC092127.1 | 0.005464063 | 0.131147199 | 4.58506918 | 0.00195818 | 0.00315729 |
| LINC02057 | 1.047571593 | 0.206359061 | -2.343820154 | 3.70E-20 | 9.18E-19 |
| AC008763.1 | 0.089691184 | 0.366473905 | 2.030672381 | 1.57E-10 | 7.44E-10 |
| AP001453.3 | 0.737340923 | 1.635368454 | 1.149211981 | 4.44E-15 | 4.16E-14 |
| AC067751.1 | 0.025386333 | 0.058342029 | 1.200483521 | 0.03371427 | 0.04381408 |
| AL592301.1 | 0.0611878 | 0.307188024 | 2.327806041 | 1.14E-07 | 3.53E-07 |
| LINC01356 | 0.087539085 | 0.569020663 | 2.700481832 | 8.40E-19 | 1.49E-17 |
| AC009227.1 | 0.018137003 | 0.007556987 | -1.263052975 | 9.79E-09 | 3.52E-08 |
| AC005089.1 | 0.018482306 | 0.468962756 | 4.665256706 | 3.47E-22 | 1.44E-20 |
| AL356966.1 | 0.024687573 | 0.089483143 | 1.857830895 | 2.39E-07 | 7.04E-07 |
| AC099524.1 | 0.162992976 | 0.061043803 | -1.41689304 | 4.27E-12 | 2.57E-11 |
| LINC02331 | 0.003666837 | 0.184656188 | 5.654161636 | 4.38E-11 | 2.25E-10 |
| LINC02594 | 0.038353299 | 0.119973897 | 1.645297962 | 0.00108079 | 0.0018162 |
| AC246787.2 | 0.346417728 | 0.096535249 | -1.843385033 | 1.06E-14 | 9.28E-14 |
| ITGB1-DT | 0.082569566 | 0.213963709 | 1.373684095 | 1.26E-08 | 4.47E-08 |
| AC011363.1 | 0.0237131 | 0.089980294 | 1.923924802 | 0.01377388 | 0.01919964 |
| AC005884.1 | 0.109102605 | 0.046680021 | -1.224808454 | 3.72E-26 | 5.20E-24 |
| AC020763.1 | 0.016714187 | 0.116406389 | 2.800025197 | 1.61E-06 | 4.21E-06 |
| AC073611.1 | 0.402420009 | 1.153091816 | 1.518733447 | 9.13E-21 | 2.65E-19 |
| AC007285.2 | 0.056958928 | 0.342730951 | 2.589082593 | 5.24E-09 | 1.97E-08 |
| AC110285.3 | 0.019897283 | 0.329822084 | 4.051044633 | 3.37E-07 | 9.74E-07 |
| RNF144A-AS1 | 0.045955962 | 0.126762795 | 1.463807429 | 4.87E-07 | 1.38E-06 |
| AC048382.5 | 0.292130302 | 0.126758294 | -1.204531865 | 3.53E-11 | 1.84E-10 |
| LINC00173 | 0.102701819 | 0.265229902 | 1.368781706 | 0.00192852 | 0.00311407 |
| AC087289.1 | 0.059249003 | 0.133143336 | 1.168117441 | 0.00093632 | 0.0015886 |
| AC092115.3 | 0.070796762 | 0.282993342 | 1.999012827 | 8.62E-09 | 3.14E-08 |
| AC073365.1 | 0.000496141 | 0.705478919 | 10.47363644 | 2.08E-11 | 1.12E-10 |
| AL355001.1 | 0.075816808 | 0.193746057 | 1.353577317 | 0.01540199 | 0.02125717 |
| LINC01354 | 0.137106252 | 0.032927457 | -2.057931341 | 6.91E-18 | 1.05E-16 |
| AC010761.1 | 0.92049821 | 2.478144797 | 1.428773665 | 2.36E-16 | 2.75E-15 |
| PLUT | 0.008339645 | 0.354421128 | 5.409334758 | 1.21E-20 | 3.37E-19 |
| FENDRR | 5.782781396 | 1.690043037 | -1.774703581 | 2.38E-20 | 6.21E-19 |
| AL512656.1 | 0.064922731 | 0.280999489 | 2.113771907 | 2.83E-07 | 8.26E-07 |
| AC092171.3 | 0.390785773 | 1.031613022 | 1.400452038 | 2.13E-16 | 2.51E-15 |
| AL358075.2 | 0.046690748 | 0.173851879 | 1.896650041 | 4.40E-05 | 9.19E-05 |
| AC021683.2 | 0.307776379 | 0.117858308 | -1.384829056 | 7.33E-11 | 3.65E-10 |
| FALEC | 0.1089827 | 0.299305844 | 1.457521313 | 1.77E-06 | 4.59E-06 |
| AC063944.3 | 0.058965903 | 0.023623997 | -1.319627873 | 1.15E-09 | 4.74E-09 |
| FGF10-AS1 | 0.037365779 | 0.01823315 | -1.035153784 | 7.98E-11 | 3.95E-10 |
| AP003355.2 | 0.041748659 | 0.019825122 | -1.074400114 | 2.68E-19 | 5.37E-18 |
| AC007207.2 | 0.030603892 | 0.229844251 | 2.908869522 | 0.00139268 | 0.00230059 |
| AC034199.1 | 0.018021071 | 0.082088604 | 2.187497171 | 1.27E-05 | 2.89E-05 |
| AL354726.1 | 0.062689769 | 0.420919994 | 2.747244119 | 0.01283352 | 0.01798078 |
| AC116407.2 | 0.306298376 | 0.786720691 | 1.360913809 | 1.98E-08 | 6.82E-08 |
| AC104985.1 | 0.034379566 | 0.014636623 | -1.23196855 | 1.47E-09 | 5.95E-09 |
| AL356481.3 | 0.250748129 | 0.509346387 | 1.022408174 | 6.66E-06 | 1.58E-05 |
| AC136475.7 | 0.046186283 | 0.327002831 | 2.823766764 | 9.43E-10 | 3.96E-09 |
| WASIR2 | 0.023333571 | 0.1857327 | 2.992748829 | 3.03E-09 | 1.18E-08 |
| FRGCA | 0.047315619 | 0.448118321 | 3.243491312 | 1.71E-09 | 6.88E-09 |
| Z98257.1 | 0.010504955 | 0.045889716 | 2.127100927 | 0.01212016 | 0.0170514 |
| AC023830.1 | 0.026302093 | 0.089157596 | 1.761180111 | 0.00982456 | 0.01400244 |
| LINC02106 | 0.066168594 | 0.014608788 | -2.179310132 | 3.65E-17 | 4.90E-16 |
| AC015987.1 | 0.069687444 | 0.246267503 | 1.82125561 | 0.00023614 | 0.00043903 |
| Z94721.2 | 0.019090403 | 0.07828908 | 2.035963537 | 0.00217057 | 0.00346998 |
| AC017076.1 | 0.03502884 | 0.10409116 | 1.571232417 | 0.00918865 | 0.01316181 |
| LINC01556 | 0.175130645 | 0.457038585 | 1.383884418 | 0.00607259 | 0.00900811 |
| AC009955.3 | 0.027583022 | 0.118921827 | 2.108161086 | 0.00027419 | 0.00050546 |
| AC006017.1 | 0.085190027 | 0.292969183 | 1.781992461 | 2.88E-05 | 6.17E-05 |
| AC117386.2 | 0.002790788 | 0.377752919 | 7.080626307 | 1.35E-15 | 1.39E-14 |
| AC023825.2 | 0.037048341 | 0.319858343 | 3.109952277 | 3.39E-08 | 1.13E-07 |
| LINC01135 | 0.377176976 | 0.118572034 | -1.669477832 | 5.08E-20 | 1.22E-18 |
| AC093627.6 | 0.91602042 | 0.138482771 | -2.725673265 | 1.78E-14 | 1.51E-13 |
| LINC02554 | 0.039401775 | 0.011362396 | -1.793993509 | 4.55E-21 | 1.41E-19 |
| AC084033.3 | 1.253914934 | 0.612378205 | -1.033944637 | 1.86E-12 | 1.17E-11 |
| AC097532.2 | 0.015168494 | 0.040220298 | 1.406845945 | 0.00250434 | 0.00396981 |
| AC008781.1 | 0.07367414 | 0.28249166 | 1.938978047 | 0.00177979 | 0.00289189 |
| ARHGEF26-AS1 | 0.084498388 | 0.025003979 | -1.756766108 | 1.83E-10 | 8.63E-10 |
| LINC01563 | 0.047024762 | 0.018043844 | -1.38191389 | 2.66E-06 | 6.76E-06 |
| AC011476.2 | 0.035961562 | 0.10473186 | 1.542172788 | 0.0007371 | 0.00127519 |
| RBAKDN | 0.010333846 | 0.125276771 | 3.59966978 | 4.03E-06 | 9.93E-06 |
| FLJ45513 | 0.163960273 | 0.375651519 | 1.19604864 | 5.77E-06 | 1.39E-05 |
| UVRAG-DT | 0.0241409 | 0.095613932 | 1.985741406 | 1.26E-11 | 7.05E-11 |
| AC005785.1 | 0.166398356 | 0.448873712 | 1.431668432 | 1.01E-12 | 6.72E-12 |
| CYTOR | 0.96217326 | 2.713381492 | 1.495723288 | 7.69E-19 | 1.37E-17 |
| AC009148.1 | 0.068113719 | 0.191063352 | 1.488033781 | 1.57E-07 | 4.76E-07 |
| AC012363.1 | 0.016759107 | 0.379876557 | 4.502513506 | 2.39E-12 | 1.48E-11 |
| LINC02253 | 0.047895682 | 2.69741375 | 5.815537421 | 8.86E-17 | 1.11E-15 |
| AL357079.1 | 0.477407304 | 0.962593485 | 1.011706021 | 6.40E-07 | 1.78E-06 |
| AL359878.1 | 0.04261422 | 0.089644916 | 1.072886848 | 0.00158842 | 0.00260657 |
| CYP4A22-AS1 | 0.066669682 | 0.344050731 | 2.367518556 | 3.77E-17 | 5.05E-16 |
| AC005261.1 | 3.001028503 | 6.402482197 | 1.093174315 | 2.75E-14 | 2.26E-13 |
| LINC01267 | 0.005745214 | 0.056927399 | 3.308690748 | 2.35E-07 | 6.96E-07 |
| AL109910.2 | 0.059817962 | 0.013376342 | -2.160895086 | 1.37E-19 | 2.92E-18 |
| AC100803.3 | 0.033970429 | 0.163926755 | 2.27069998 | 3.13E-10 | 1.42E-09 |
| AC020916.1 | 6.092854165 | 15.39163669 | 1.336956536 | 1.06E-11 | 5.98E-11 |
| AC010148.1 | 0.025937732 | 0.101856735 | 1.973417121 | 9.09E-11 | 4.46E-10 |
| AC009032.1 | 0.069943461 | 1.448730662 | 4.372456415 | 0.03123166 | 0.04082125 |
| AC005387.1 | 0.181225357 | 0.402138419 | 1.149907343 | 1.58E-06 | 4.14E-06 |
| AF106564.1 | 0.017817108 | 0.006336483 | -1.491508886 | 5.28E-15 | 4.87E-14 |
| AL450992.1 | 0.009537551 | 0.03587583 | 1.911321469 | 4.31E-05 | 9.01E-05 |
| AC004039.1 | 0.017423069 | 0.087940965 | 2.335536579 | 4.18E-06 | 1.03E-05 |
| AC009831.1 | 0.702008505 | 0.343394127 | -1.031623142 | 7.01E-20 | 1.62E-18 |
| AL391425.1 | 0.008034261 | 0.033727603 | 2.069692585 | 0.00476332 | 0.00720023 |
| AC018653.3 | 0.43854101 | 1.164931082 | 1.40946094 | 3.25E-10 | 1.46E-09 |
| AC020612.3 | 0.057124752 | 0.248880022 | 2.123262523 | 0.00119536 | 0.00199276 |
| LINC01537 | 0.176256878 | 0.044240946 | -1.994225425 | 3.70E-19 | 7.18E-18 |
| AC010913.1 | 0.021972042 | 0.113980061 | 2.375040583 | 6.15E-16 | 6.70E-15 |
| AL035461.2 | 0.907000534 | 2.279155205 | 1.329323867 | 1.95E-11 | 1.06E-10 |
| LINC00624 | 0.040940005 | 0.19863949 | 2.278569281 | 1.98E-06 | 5.11E-06 |
| LINC01549 | 0.018691677 | 0.244501939 | 3.709378016 | 4.96E-06 | 1.21E-05 |
| LINC02195 | 0.054761624 | 0.573376973 | 3.388246831 | 9.97E-12 | 5.65E-11 |
| AC099792.1 | 0.024265211 | 0.365324549 | 3.912217411 | 8.38E-14 | 6.46E-13 |
| AC105429.1 | 0.037482552 | 0.284337034 | 2.923310926 | 1.43E-17 | 2.06E-16 |
| AC012640.1 | 0.019084636 | 0.111607228 | 2.547946874 | 8.60E-10 | 3.63E-09 |
| AC099521.1 | 0.009056494 | 0.032509668 | 1.84384426 | 0.01297114 | 0.01815494 |
| AL626787.1 | 0.361606793 | 0.12510567 | -1.531274601 | 3.71E-12 | 2.25E-11 |
| ZFHX2-AS1 | 0.078171281 | 0.162347462 | 1.054374255 | 1.98E-06 | 5.11E-06 |
| AL450332.1 | 0.03379489 | 0.016844521 | -1.004525699 | 3.31E-05 | 7.02E-05 |
| AC027307.1 | 0.091582646 | 0.354703615 | 1.953467883 | 1.04E-11 | 5.89E-11 |
| AC087241.3 | 0.150824102 | 0.045313853 | -1.734842925 | 1.65E-05 | 3.70E-05 |
| AC007785.3 | 0.027748793 | 0.122539091 | 2.142745112 | 9.56E-07 | 2.58E-06 |
| AC025280.2 | 0.056275815 | 0.020977603 | -1.423665213 | 1.05E-12 | 6.93E-12 |
| AC007285.1 | 0.075162448 | 0.18843867 | 1.326011092 | 0.00318921 | 0.0049617 |
| TSPOAP1-AS1 | 0.330672674 | 1.009245475 | 1.609801385 | 2.88E-10 | 1.31E-09 |
| AC131971.1 | 0.053365264 | 0.757544619 | 3.827357983 | 2.68E-05 | 5.77E-05 |
| AL353801.1 | 0.689522188 | 0.120120665 | -2.521112611 | 1.19E-22 | 5.43E-21 |
| AC024941.2 | 0.998715827 | 0.457011134 | -1.127844921 | 7.27E-13 | 4.94E-12 |
| AC026336.3 | 0.001230483 | 0.938071039 | 9.574328729 | 4.71E-16 | 5.24E-15 |
| AC078778.1 | 0.183829004 | 0.861172525 | 2.227937884 | 2.19E-14 | 1.82E-13 |
| AL031055.1 | 0.102904451 | 0.20729271 | 1.010363991 | 0.00022326 | 0.00041706 |
| AL035071.1 | 1.866222205 | 3.86914545 | 1.051894191 | 1.38E-08 | 4.88E-08 |
| C8orf37-AS1 | 0.029433512 | 0.080021011 | 1.44291917 | 0.00013462 | 0.0002594 |
| AC106772.2 | 0.819118505 | 0.264218361 | -1.632341461 | 6.95E-05 | 0.00014014 |
| LINC02603 | 0.036482362 | 0.075417305 | 1.047696459 | 0.00168663 | 0.00275531 |
| SMG7-AS1 | 0.094115808 | 0.207424015 | 1.140073965 | 6.39E-12 | 3.71E-11 |
| AC009102.2 | 0.243469094 | 0.028589446 | -3.090184094 | 2.81E-19 | 5.62E-18 |
| C6orf99 | 0.350392804 | 0.716016918 | 1.031020527 | 1.04E-09 | 4.33E-09 |
| RASGRF2-AS1 | 0.028149267 | 0.061069696 | 1.117359278 | 0.00608315 | 0.00901887 |
| AC140847.2 | 0.034932009 | 0.015009368 | -1.218686419 | 2.33E-13 | 1.68E-12 |
| AC096642.1 | 0.068391905 | 0.161310387 | 1.237941857 | 0.00079862 | 0.0013743 |
| AP002478.1 | 0.011877314 | 0.193257584 | 4.024244483 | 2.70E-11 | 1.43E-10 |
| AC007277.1 | 0.010977443 | 0.488040267 | 5.474386275 | 1.37E-14 | 1.18E-13 |
| FAM83A-AS1 | 0.015346893 | 0.068786969 | 2.164188672 | 0.00110187 | 0.00184934 |
| AC110769.2 | 0.063349259 | 0.181075211 | 1.51518941 | 0.0002253 | 0.00042046 |
| A2M-AS1 | 0.554164143 | 0.2264022 | -1.291425391 | 5.67E-13 | 3.91E-12 |
| AC005954.1 | 0.074385851 | 0.156219901 | 1.070478117 | 0.01881107 | 0.02556111 |
| AC009095.1 | 0.124536336 | 0.252140045 | 1.017658531 | 0.01510954 | 0.02090115 |
| AC126773.2 | 0.042347391 | 0.23103775 | 2.447783611 | 8.48E-20 | 1.89E-18 |
| LINC02254 | 0.006212214 | 0.191388436 | 4.945252308 | 2.32E-08 | 7.91E-08 |
| AC000123.1 | 0.921666073 | 1.949252651 | 1.080605046 | 9.02E-07 | 2.44E-06 |
| AC007128.2 | 0.007648411 | 0.352826213 | 5.527653906 | 5.16E-19 | 9.64E-18 |
| AC004816.1 | 1.065580248 | 2.153564142 | 1.015087049 | 3.23E-10 | 1.46E-09 |
| AC007684.1 | 0.063665225 | 0.479311401 | 2.912385797 | 0.01503077 | 0.0208133 |
| AP002360.2 | 0.040925401 | 0.137224102 | 1.74546543 | 1.28E-05 | 2.90E-05 |
| AC025265.3 | 0.721887622 | 0.291779866 | -1.306893932 | 3.43E-14 | 2.78E-13 |
| AL596247.1 | 0.053409144 | 0.437076586 | 3.032727425 | 0.00283192 | 0.00444515 |
| AC020915.2 | 0.243234207 | 0.721251698 | 1.568156675 | 4.68E-05 | 9.72E-05 |
| HAGLR | 9.062518224 | 2.35311151 | -1.945342302 | 2.05E-21 | 6.82E-20 |
| AL139020.1 | 0.100491137 | 0.016931272 | -2.56930603 | 1.58E-12 | 1.01E-11 |
| LINC01475 | 0.544701317 | 0.124134453 | -2.133561765 | 7.64E-19 | 1.37E-17 |
| PROX1-AS1 | 0.032032826 | 0.14788477 | 2.206850497 | 7.88E-13 | 5.34E-12 |
| AC007556.1 | 0.489560966 | 0.066423022 | -2.881733271 | 2.42E-26 | 3.57E-24 |
| AL365361.1 | 1.481368712 | 0.374605233 | -1.983487813 | 1.66E-19 | 3.54E-18 |
| LINC01996 | 0.008714436 | 0.31279122 | 5.165648981 | 2.49E-13 | 1.78E-12 |
| LINC02441 | 15.03200112 | 2.962197084 | -2.343297544 | 3.49E-22 | 1.44E-20 |
| AC024243.1 | 0.028201062 | 0.0751856 | 1.414706862 | 8.89E-05 | 0.00017622 |
| AL118505.1 | 1.366290399 | 2.76545427 | 1.017252331 | 4.20E-05 | 8.80E-05 |
| AC034213.1 | 0.0176958 | 0.090633786 | 2.356641959 | 0.00197393 | 0.00318081 |
| AC007608.1 | 0.03757877 | 0.510756214 | 3.764645096 | 1.04E-05 | 2.40E-05 |
| AC026356.1 | 0.182409804 | 0.822678164 | 2.173144877 | 3.05E-17 | 4.14E-16 |
| AL136115.2 | 0.055318641 | 0.777498055 | 3.813001443 | 2.70E-05 | 5.80E-05 |
| AC007608.3 | 0.016757186 | 0.055297549 | 1.722435675 | 6.59E-05 | 0.00013348 |
| AL049539.1 | 0.048230769 | 0.695946392 | 3.850950455 | 3.99E-14 | 3.20E-13 |
| AP000777.2 | 0.031159003 | 0.086129716 | 1.466862012 | 0.00012629 | 0.00024437 |
| RBPMS-AS1 | 0.212029749 | 0.652908885 | 1.62261498 | 5.52E-11 | 2.80E-10 |
| AC009133.3 | 0.343738687 | 0.061232439 | -2.488944186 | 2.65E-20 | 6.87E-19 |
| AC004053.1 | 0.055722245 | 0.02173508 | -1.358227974 | 1.41E-10 | 6.76E-10 |
| RPS6KA2-IT1 | 0.031426156 | 0.128800604 | 2.03510163 | 0.00365745 | 0.00563075 |
| ZFPM2-AS1 | 0.032026066 | 0.171319889 | 2.419374139 | 2.76E-08 | 9.29E-08 |
| GDNF-AS1 | 0.068368864 | 0.026347072 | -1.375696811 | 1.82E-11 | 9.86E-11 |
| AC011498.3 | 0.014795474 | 0.150501866 | 3.346553603 | 2.18E-09 | 8.67E-09 |
| LAMC1-AS1 | 0.195440608 | 0.40402735 | 1.047722701 | 0.01321454 | 0.01848138 |
| MMP25-AS1 | 0.366275592 | 0.984318935 | 1.426196282 | 2.76E-11 | 1.46E-10 |
| AC008456.1 | 0.024981742 | 0.394293293 | 3.9803232 | 3.14E-07 | 9.11E-07 |
| AC233280.1 | 0.042164286 | 0.117081847 | 1.473423968 | 1.42E-06 | 3.74E-06 |
| AC040934.1 | 0.072092145 | 0.249252604 | 1.78969459 | 0.00063764 | 0.00111372 |
| AL512506.1 | 0.06253776 | 0.464946923 | 2.894266581 | 6.36E-07 | 1.77E-06 |
| PANTR1 | 0.117873093 | 0.02359758 | -2.320523641 | 4.46E-28 | 8.38E-26 |
| RNF157-AS1 | 0.080426273 | 0.189924118 | 1.239684358 | 4.83E-06 | 1.18E-05 |
| AL365356.3 | 0.060799656 | 0.018154465 | -1.74373876 | 1.75E-10 | 8.25E-10 |
| AC022973.4 | 0.087261549 | 0.273628852 | 1.648802368 | 0.0001042 | 0.00020446 |
| AC020659.1 | 0.036204333 | 0.687208534 | 4.246513672 | 1.98E-15 | 1.97E-14 |
| AC004253.1 | 0.343672313 | 0.841821973 | 1.292481537 | 1.04E-06 | 2.79E-06 |
| SNHG25 | 0.96788818 | 29.11773565 | 4.910913975 | 5.61E-20 | 1.32E-18 |
| AC022445.1 | 0.01501391 | 0.15027927 | 3.323274377 | 0.00017034 | 0.0003232 |
| LINC02447 | 0.141126617 | 0.055076673 | -1.3574768 | 4.77E-16 | 5.28E-15 |
| AL033397.2 | 0.115674179 | 0.277420232 | 1.262006146 | 0.0147158 | 0.02041344 |
| AC087482.1 | 0.600131591 | 0.142808595 | -2.07119606 | 1.72E-10 | 8.14E-10 |
| AC108058.1 | 0.271417454 | 0.773865898 | 1.511570087 | 0.01144567 | 0.01615668 |
| AC007390.2 | 0.177124778 | 0.392587854 | 1.148249493 | 0.00072937 | 0.00126303 |
| AL353150.1 | 0.159943683 | 1.065007769 | 2.735228032 | 6.31E-19 | 1.16E-17 |
| CDC42-IT1 | 0.0263021 | 0.292618805 | 3.47577257 | 0.00144103 | 0.00237687 |
| AC004990.1 | 0.696659554 | 0.251796499 | -1.468195584 | 1.11E-13 | 8.36E-13 |
| LMO7-AS1 | 0.078464889 | 0.758371016 | 3.272784691 | 1.24E-17 | 1.82E-16 |
| AP000911.1 | 0.15313057 | 0.053589923 | -1.514728664 | 2.36E-10 | 1.09E-09 |
| AC004466.3 | 0.070637716 | 0.299278621 | 2.082978612 | 2.60E-05 | 5.62E-05 |
| AP005899.1 | 0.223091025 | 0.656699068 | 1.557599931 | 0.00011062 | 0.00021597 |
| AL592429.2 | 0.114036319 | 0.027018081 | -2.077496261 | 1.22E-20 | 3.38E-19 |
| LINC01271 | 0.01168575 | 0.046183346 | 1.982622382 | 5.11E-07 | 1.44E-06 |
| C1orf137 | 0.066353386 | 0.01028418 | -2.68974331 | 1.37E-27 | 2.34E-25 |
| AC007671.1 | 0.090856622 | 0.031132405 | -1.545174634 | 4.54E-12 | 2.71E-11 |
| FAM222A-AS1 | 0.079788575 | 1.064712411 | 3.738137799 | 2.38E-22 | 1.04E-20 |
| AC010331.1 | 0.22246483 | 0.463332122 | 1.058469427 | 2.15E-06 | 5.52E-06 |
| AC073316.3 | 0.116014953 | 0.049321308 | -1.234027791 | 3.97E-12 | 2.40E-11 |
| AC012313.8 | 0.039255031 | 0.118719826 | 1.596611433 | 9.30E-14 | 7.09E-13 |
| AC021074.3 | 0.032052698 | 0.010727264 | -1.579163617 | 3.44E-10 | 1.54E-09 |
| AC016735.1 | 2.0027528 | 6.722739683 | 1.747064927 | 1.71E-11 | 9.28E-11 |
| AC092436.4 | 0.036723565 | 0.326888619 | 3.154021115 | 0.02805116 | 0.03697437 |
| AL160313.1 | 0.122292212 | 0.04762634 | -1.360500948 | 2.23E-15 | 2.20E-14 |
| PGM5P4-AS1 | 0.058584455 | 0.01252855 | -2.225298434 | 1.94E-22 | 8.52E-21 |
| AC087683.2 | 0.085126269 | 0.338767951 | 1.992621097 | 0.02553278 | 0.03385126 |
| AL133243.3 | 0.144991761 | 0.430247134 | 1.569194662 | 9.79E-06 | 2.27E-05 |
| AL591845.1 | 0.644059224 | 2.068129212 | 1.683061062 | 3.20E-10 | 1.45E-09 |
| PLA2G4C-AS1 | 0.043999508 | 0.092568536 | 1.073034504 | 0.02383327 | 0.03182244 |
| STEAP2-AS1 | 0.005447164 | 0.055420659 | 3.34684661 | 2.10E-08 | 7.20E-08 |
| AC099518.1 | 0.088380801 | 0.261167346 | 1.563169607 | 1.15E-06 | 3.08E-06 |
| AL022316.1 | 0.136962743 | 0.947926381 | 2.790991515 | 1.80E-10 | 8.51E-10 |
| AC005392.2 | 14.79930341 | 2.686777928 | -2.461580284 | 1.37E-11 | 7.63E-11 |
| AL139082.1 | 0.070016979 | 0.157503885 | 1.169610698 | 0.00195733 | 0.00315686 |
| KCNH1-IT1 | 0.040770944 | 0.017368324 | -1.231082806 | 3.05E-10 | 1.38E-09 |
| AC087516.2 | 0.022458632 | 0.00540872 | -2.053910948 | 1.98E-14 | 1.66E-13 |
| AP003071.4 | 1.240984941 | 0.342559078 | -1.857060885 | 2.00E-11 | 1.08E-10 |
| U62631.1 | 0.305405552 | 0.098264091 | -1.635990076 | 2.83E-08 | 9.48E-08 |
| AC015908.4 | 0.119160917 | 0.039238369 | -1.602574155 | 1.38E-20 | 3.77E-19 |
| AC010999.2 | 0.063661074 | 0.16269427 | 1.353680048 | 4.76E-05 | 9.88E-05 |
| AL157838.1 | 0.268042508 | 0.603444807 | 1.17076001 | 0.00020602 | 0.00038632 |
| AP002812.5 | 0.096069887 | 0.251405979 | 1.387862762 | 0.01469368 | 0.02038794 |
| AC099314.1 | 0.014676446 | 0.087608757 | 2.577572464 | 5.76E-05 | 0.00011784 |
| AL590483.1 | 0.037415543 | 0.575426576 | 3.942922245 | 3.50E-16 | 3.98E-15 |
| AP000845.1 | 0.055749912 | 0.11266244 | 1.014965198 | 8.34E-05 | 0.0001662 |
| AC006237.1 | 0.257927308 | 0.084896652 | -1.603184956 | 4.76E-16 | 5.28E-15 |
| AC006273.1 | 0.027172742 | 0.102146169 | 1.91040301 | 3.71E-07 | 1.07E-06 |
| AP005264.1 | 0.14644779 | 0.054024512 | -1.438700382 | 0.00159859 | 0.0026209 |
| LINC00698 | 0.002704689 | 0.067747433 | 4.646631798 | 6.01E-13 | 4.12E-12 |
| AP001107.5 | 1.142434599 | 0.167161035 | -2.77280108 | 1.16E-16 | 1.42E-15 |
| AC068790.6 | 0.090242433 | 0.387415542 | 2.102003955 | 0.01015296 | 0.01445161 |
| TUSC8 | 0.859314718 | 3.138400847 | 1.868771121 | 1.65E-06 | 4.30E-06 |
| AL772337.2 | 0.04950621 | 0.006553587 | -2.917252807 | 7.18E-40 | 1.31E-36 |
| AC016831.4 | 0.050568129 | 0.99338723 | 4.296055906 | 6.18E-06 | 1.47E-05 |
| OSBPL10-AS1 | 0.004626077 | 0.106952667 | 4.531039508 | 0.00066308 | 0.0011552 |
| AC090527.3 | 0.080690393 | 0.221632513 | 1.457700713 | 5.79E-05 | 0.00011852 |
| AL158847.1 | 0.096446303 | 0.03196325 | -1.593311837 | 1.06E-15 | 1.11E-14 |
| AC005307.1 | 0.045997132 | 0.38663283 | 3.071348338 | 0.00673176 | 0.00988109 |
| LINC01719 | 0.103172565 | 0.239785138 | 1.21668285 | 3.49E-07 | 1.01E-06 |
| AL596223.1 | 0.638889531 | 0.203955919 | -1.647309125 | 1.14E-13 | 8.59E-13 |
| AL355075.2 | 0.397000861 | 0.886025004 | 1.158205276 | 2.66E-05 | 5.74E-05 |
| Z69733.1 | 0.078954081 | 0.160454009 | 1.023074098 | 0.02716684 | 0.03586076 |
| AC097358.2 | 0.01442131 | 0.081841184 | 2.504624777 | 6.09E-11 | 3.06E-10 |
| TSPEAR-AS2 | 0.038788487 | 0.356988198 | 3.202175984 | 6.09E-12 | 3.56E-11 |
| AC114488.2 | 0.081946356 | 0.23749589 | 1.535150847 | 2.29E-06 | 5.85E-06 |
| AC005256.1 | 0.006583988 | 0.909160092 | 7.109428826 | 2.14E-17 | 2.97E-16 |
| FAM30A | 0.774286367 | 0.206710691 | -1.905254238 | 4.51E-17 | 5.93E-16 |
| AL359881.2 | 0.026872604 | 0.224062588 | 3.05969375 | 4.55E-08 | 1.48E-07 |
| AL442128.2 | 0.069658828 | 0.19987605 | 1.520727508 | 4.17E-07 | 1.19E-06 |
| AC106820.2 | 0.005375993 | 0.019278749 | 1.842408159 | 0.00072438 | 0.00125517 |
| AL136307.1 | 0.049225294 | 0.338406866 | 2.7812871 | 6.87E-06 | 1.63E-05 |
| AC026461.2 | 0.212505749 | 0.029021738 | -2.872296062 | 7.20E-37 | 5.61E-34 |
| AC079779.1 | 0.005543051 | 0.001631422 | -1.764550735 | 1.26E-20 | 3.45E-19 |
| AC105219.3 | 0.058620173 | 0.411660607 | 2.811986271 | 1.18E-20 | 3.31E-19 |
| AC004551.1 | 0.029462995 | 0.145310103 | 2.30215902 | 4.27E-05 | 8.94E-05 |
| AC087741.1 | 0.324156472 | 0.821578119 | 1.341707382 | 1.34E-10 | 6.44E-10 |
| AC109992.2 | 0.036006628 | 0.134347248 | 1.899632369 | 0.00604971 | 0.00897662 |
| AC011466.3 | 0.049683613 | 0.176063766 | 1.825256037 | 4.31E-09 | 1.63E-08 |
| AC073283.1 | 0.052729573 | 0.551519218 | 3.386726945 | 2.58E-14 | 2.13E-13 |
| AC115522.1 | 0.278236326 | 0.56316256 | 1.017240635 | 6.28E-06 | 1.50E-05 |
| LINC01197 | 0.14815984 | 0.064022508 | -1.210503337 | 9.77E-14 | 7.43E-13 |
| AC096734.2 | 0.145387455 | 0.046530027 | -1.643668855 | 3.30E-13 | 2.34E-12 |
| AC036214.1 | 0.005090837 | 0.026921754 | 2.402797752 | 3.52E-05 | 7.45E-05 |
| AL035071.2 | 0.079781599 | 0.573804884 | 2.846432307 | 1.03E-05 | 2.37E-05 |
| AL807757.2 | 0.025910136 | 0.274891593 | 3.40727429 | 2.29E-05 | 5.01E-05 |
| AC004817.3 | 0.005089931 | 0.032978538 | 2.695809385 | 6.02E-05 | 0.0001228 |
| AL031186.1 | 0.425978496 | 0.86143193 | 1.015956197 | 1.53E-07 | 4.66E-07 |
| ABCA9-AS1 | 0.003798246 | 0.096290084 | 4.663982099 | 2.18E-07 | 6.47E-07 |
| AL109615.2 | 0.003583482 | 0.07216213 | 4.331808001 | 1.96E-10 | 9.18E-10 |
| VPS9D1-AS1 | 1.281297237 | 7.816070012 | 2.608838197 | 4.69E-22 | 1.85E-20 |
| AC005618.1 | 0.130727384 | 0.317025946 | 1.27803954 | 0.00064419 | 0.00112408 |
| HAND2-AS1 | 1.763418924 | 0.099036388 | -4.154272745 | 3.03E-20 | 7.77E-19 |
| CASC9 | 1.855974177 | 4.790518616 | 1.368005211 | 6.33E-06 | 1.51E-05 |
| AC079354.3 | 0.036431321 | 0.145224772 | 1.995036351 | 1.08E-05 | 2.48E-05 |
| AC018521.6 | 1.381136117 | 0.661864085 | -1.061248618 | 1.79E-15 | 1.80E-14 |
| LINC01473 | 0.197177622 | 0.810155174 | 2.038702436 | 7.31E-10 | 3.12E-09 |
| AC104088.1 | 0.259693009 | 0.86084206 | 1.728941392 | 2.49E-10 | 1.15E-09 |
| AC108865.2 | 0.285568209 | 2.121047158 | 2.892869414 | 0.01354202 | 0.01891028 |
| LINC02340 | 0.041901285 | 0.179656024 | 2.100170915 | 5.31E-06 | 1.28E-05 |
| C9orf147 | 0.101223877 | 0.209746916 | 1.051099955 | 7.58E-07 | 2.08E-06 |
| AC106772.1 | 0.013187309 | 0.249136235 | 4.239712727 | 5.54E-14 | 4.37E-13 |
| AL590096.1 | 0.09579873 | 0.263147682 | 1.457794254 | 9.17E-07 | 2.48E-06 |
| AF230666.2 | 0.008979224 | 0.117328901 | 3.70782386 | 1.98E-05 | 4.37E-05 |
| ERVH-1 | 1.014190281 | 0.346700887 | -1.548564924 | 1.28E-15 | 1.33E-14 |
| AC009121.2 | 0.124683382 | 0.259695594 | 1.058552341 | 5.81E-07 | 1.63E-06 |
| AP001429.1 | 0.056636393 | 1.107091803 | 4.288901666 | 2.70E-07 | 7.89E-07 |
| AC127024.3 | 0.032583074 | 0.39508197 | 3.599957381 | 6.31E-05 | 0.00012831 |
| AC007038.2 | 0.462811093 | 1.193304684 | 1.366467099 | 2.43E-07 | 7.15E-07 |
| AC026401.2 | 0.019631666 | 0.14123976 | 2.846891795 | 0.00182866 | 0.00296335 |
| C2-AS1 | 0.027496825 | 0.294056812 | 3.41875796 | 1.03E-14 | 9.09E-14 |
| AP001469.3 | 0.367466115 | 1.069319339 | 1.541009636 | 3.59E-19 | 7.00E-18 |
| AP003559.1 | 0.113630832 | 0.379109619 | 1.738260724 | 1.50E-08 | 5.26E-08 |
| AC007495.1 | 0.082429908 | 0.020728656 | -1.99154133 | 2.91E-16 | 3.34E-15 |
| AC103736.1 | 0.069937894 | 0.030145884 | -1.21411332 | 2.11E-12 | 1.32E-11 |
| AP001627.1 | 0.741404434 | 0.084928949 | -3.125932438 | 8.68E-18 | 1.31E-16 |
| FAM182B | 0.027026457 | 0.05926964 | 1.13292091 | 0.00354306 | 0.00546698 |
| AC048344.4 | 0.074043107 | 0.545113531 | 2.880119396 | 2.42E-16 | 2.82E-15 |
| TRIM31-AS1 | 1.366672614 | 2.982842226 | 1.126019983 | 9.56E-09 | 3.45E-08 |
| MIRLET7BHG | 0.202839466 | 0.573420941 | 1.499256212 | 1.52E-09 | 6.15E-09 |
| AC004066.2 | 0.261851216 | 0.530019893 | 1.017299206 | 0.00569934 | 0.00849371 |
| AC004221.1 | 0.015471672 | 0.048545475 | 1.649707743 | 0.00479642 | 0.0072371 |
| AC068790.3 | 0.145941251 | 0.489110452 | 1.744772571 | 0.00268831 | 0.00423926 |
| CASC18 | 0.17463968 | 0.034260695 | -2.349755068 | 2.07E-15 | 2.05E-14 |
| AL121992.1 | 0.025148662 | 0.053611254 | 1.092054219 | 0.00011253 | 0.00021954 |
| CAPN10-DT | 0.290576001 | 0.745308374 | 1.358921912 | 4.87E-16 | 5.38E-15 |
| AP000640.2 | 0.013768105 | 0.043877728 | 1.672158784 | 0.00047696 | 0.00085079 |
| AC073050.1 | 0.799301201 | 0.095558287 | -3.064286367 | 1.45E-25 | 1.65E-23 |
| AL354719.2 | 0.043468169 | 0.369933999 | 3.089236682 | 1.20E-14 | 1.04E-13 |
| AC120498.6 | 0.333815713 | 0.138614332 | -1.267975435 | 2.44E-09 | 9.64E-09 |
| AP001065.1 | 0.771918086 | 0.330789204 | -1.222535609 | 9.90E-08 | 3.08E-07 |
| MAFTRR | 0.171826248 | 0.080942347 | -1.085983856 | 8.01E-11 | 3.97E-10 |
| AC097347.1 | 0.035761915 | 0.194065834 | 2.440050245 | 2.96E-05 | 6.32E-05 |
| LINC00867 | 0.021965332 | 0.105207761 | 2.25994092 | 0.00062173 | 0.00108872 |
| RHPN1-AS1 | 0.194212139 | 0.824957447 | 2.086686327 | 4.21E-16 | 4.72E-15 |
| LINC02550 | 0.345122313 | 0.146083339 | -1.240316105 | 9.89E-09 | 3.56E-08 |
| LNCOC1 | 0.014263342 | 0.032717507 | 1.197750817 | 2.45E-05 | 5.34E-05 |
| GNG12-AS1 | 0.5271958 | 0.247576933 | -1.090461976 | 6.87E-17 | 8.83E-16 |
| AC090673.1 | 0.017694512 | 0.262631372 | 3.89166536 | 1.11E-12 | 7.30E-12 |
| ZKSCAN2-DT | 0.421689297 | 1.026490439 | 1.28346788 | 2.24E-09 | 8.88E-09 |
| HSPC324 | 0.388652386 | 0.141820149 | -1.454417857 | 1.57E-17 | 2.24E-16 |
| AL513218.1 | 0.159684427 | 0.417212352 | 1.38555825 | 2.98E-07 | 8.68E-07 |
| AL021395.1 | 0.013173343 | 0.0041368 | -1.671034343 | 8.15E-27 | 1.27E-24 |
| AL513327.3 | 0.052336597 | 0.185156991 | 1.822856995 | 0.00696718 | 0.01019647 |
| AC019186.1 | 0.073512847 | 0.168040173 | 1.192737875 | 0.00059521 | 0.00104363 |
| AC022075.1 | 0.342657359 | 1.605271132 | 2.227978415 | 1.45E-07 | 4.40E-07 |
| EWSAT1 | 0.012890183 | 0.04072358 | 1.659591652 | 4.02E-05 | 8.45E-05 |
| SLC14A2-AS1 | 0.029896021 | 0.009730843 | -1.619316746 | 1.91E-10 | 8.95E-10 |
| ADD3-AS1 | 0.101049771 | 0.319808462 | 1.662142067 | 1.98E-09 | 7.89E-09 |
| SLFNL1-AS1 | 0.108702191 | 0.23410706 | 1.106787429 | 2.79E-06 | 7.05E-06 |
| AC109460.3 | 0.06710115 | 0.135328688 | 1.012058322 | 1.51E-07 | 4.58E-07 |
| AL032819.1 | 0.017632542 | 0.125301246 | 2.829088401 | 3.60E-05 | 7.61E-05 |
| AC130650.2 | 0.128000568 | 0.369293653 | 1.528618259 | 2.60E-06 | 6.60E-06 |
| AP000229.1 | 0.136398327 | 0.057037838 | -1.257834747 | 8.11E-13 | 5.47E-12 |
| AL034346.1 | 0.29479768 | 0.077561372 | -1.926314934 | 7.09E-20 | 1.63E-18 |
| AC091887.1 | 0.079656192 | 0.174084677 | 1.127930804 | 0.03649366 | 0.04717875 |
| TEX41 | 0.004162905 | 0.030871452 | 2.890610779 | 3.79E-05 | 7.98E-05 |
| AC108134.3 | 0.403313261 | 2.341315425 | 2.537346562 | 6.85E-07 | 1.90E-06 |
| DIAPH2-AS1 | 0.022961352 | 0.333844674 | 3.861897514 | 5.86E-09 | 2.19E-08 |
| AL133230.1 | 0.048179332 | 0.261173416 | 2.438521756 | 3.76E-08 | 1.24E-07 |
| AC055874.1 | 0.222127231 | 0.010980611 | -4.338356035 | 1.71E-31 | 5.50E-29 |
| AC061975.7 | 0.022741993 | 0.092227619 | 2.019840136 | 0.00066846 | 0.00116383 |
| AC093788.1 | 0.250890557 | 0.723105226 | 1.527147432 | 6.60E-08 | 2.10E-07 |
| AC007384.1 | 1.156633837 | 0.351892378 | -1.716726043 | 1.04E-18 | 1.79E-17 |
| AL024508.1 | 0.163080845 | 0.555027502 | 1.766971924 | 0.00069356 | 0.00120484 |
| LINC01082 | 6.949880676 | 1.257572363 | -2.466346791 | 1.77E-24 | 1.46E-22 |
| AC136475.3 | 0.587520443 | 7.800063485 | 3.730774909 | 6.63E-15 | 6.03E-14 |
| L29074.1 | 0.018016518 | 0.0943428 | 2.388592191 | 0.00024379 | 0.00045264 |
| LINC00682 | 0.145740088 | 0.007643279 | -4.253062224 | 4.28E-34 | 1.80E-31 |
| AC093382.1 | 0.038107386 | 0.118006302 | 1.630721361 | 0.01100641 | 0.01557289 |
| AC100803.1 | 0.005734557 | 0.091580679 | 3.997289289 | 8.16E-13 | 5.50E-12 |
| SMIM2-AS1 | 3.3013877 | 1.560787835 | -1.080798134 | 2.24E-11 | 1.20E-10 |
| SMARCA5-AS1 | 0.018483542 | 0.062390107 | 1.755076015 | 0.00337758 | 0.0052294 |
| AC004231.1 | 0.051303902 | 0.362551362 | 2.821044936 | 6.37E-05 | 0.00012937 |
| AL137026.1 | 0.062447023 | 0.018668484 | -1.742028031 | 1.83E-14 | 1.54E-13 |
| AC005005.4 | 0.00401415 | 0.020470335 | 2.350368198 | 3.02E-09 | 1.18E-08 |
| CA3-AS1 | 1.813780224 | 0.249809783 | -2.860097775 | 6.03E-23 | 2.96E-21 |
| AL354707.1 | 0.79269981 | 2.009429545 | 1.341939459 | 7.19E-09 | 2.65E-08 |
| AC135012.3 | 0.406821721 | 0.089155816 | -2.189995888 | 8.10E-24 | 5.14E-22 |
| AL139289.1 | 0.247046876 | 0.608525597 | 1.300533136 | 3.82E-09 | 1.46E-08 |
| GAS5 | 23.06745049 | 59.95981416 | 1.378137351 | 6.63E-19 | 1.21E-17 |
| AC073641.1 | 0.045100202 | 0.120044789 | 1.412366969 | 0.00621225 | 0.00919029 |
| LINC01336 | 0.173540921 | 0.071751244 | -1.274200138 | 6.30E-10 | 2.72E-09 |
| AL365356.5 | 0.047901239 | 0.311888192 | 2.702894044 | 1.55E-11 | 8.53E-11 |
| AC231981.1 | 0.119760481 | 0.253671704 | 1.082810687 | 3.83E-08 | 1.26E-07 |
| SOX9-AS1 | 0.116625493 | 0.242140116 | 1.05395893 | 6.11E-06 | 1.46E-05 |
| AL353747.3 | 0.056532251 | 0.320096307 | 2.501359974 | 1.44E-05 | 3.25E-05 |
| AL096828.3 | 0.142975175 | 0.531379764 | 1.893978615 | 2.07E-12 | 1.29E-11 |
| AC018742.1 | 0.04045192 | 0.018483154 | -1.12999722 | 1.01E-07 | 3.13E-07 |
| GTF3C2-AS1 | 0.048024904 | 0.157043703 | 1.709311473 | 5.94E-10 | 2.58E-09 |
| AC008114.1 | 0.048552061 | 0.252408662 | 2.378156984 | 0.0002706 | 0.00049901 |
| AC093591.2 | 0.008931121 | 0.062966078 | 2.817661606 | 4.61E-05 | 9.59E-05 |
| AC007342.4 | 0.586684039 | 1.539022983 | 1.391359128 | 1.22E-10 | 5.88E-10 |
| GAS8-AS1 | 0.070029003 | 0.342616609 | 2.29057063 | 6.92E-07 | 1.91E-06 |
| AC027277.2 | 0.05184122 | 0.296930641 | 2.517954398 | 0.00669196 | 0.00983061 |
| AC017083.1 | 0.09625004 | 0.318469471 | 1.726296036 | 2.25E-13 | 1.62E-12 |
| AL139089.1 | 0.388858155 | 1.471617892 | 1.920087222 | 4.76E-15 | 4.44E-14 |
| FLJ46906 | 0.571628844 | 1.272747844 | 1.154796001 | 5.88E-14 | 4.61E-13 |
| AC069133.1 | 0.270687471 | 0.010860914 | -4.639410741 | 9.28E-23 | 4.40E-21 |
| AC002091.2 | 0.402771989 | 0.158085152 | -1.349261484 | 4.82E-13 | 3.38E-12 |
| CASK-AS1 | 0.052636728 | 0.154872375 | 1.556938097 | 0.0001959 | 0.00036861 |
| AC144548.1 | 0.115421436 | 0.302089105 | 1.388062966 | 2.11E-07 | 6.27E-07 |
| AL121832.1 | 0.057358206 | 0.924386491 | 4.010424362 | 3.24E-18 | 5.21E-17 |
| AC084064.1 | 0.106505886 | 0.021418493 | -2.314004276 | 9.84E-33 | 3.58E-30 |
| AP001636.3 | 0.041134219 | 0.127954303 | 1.637217698 | 0.0094969 | 0.01357447 |
| RDH10-AS1 | 0.041518454 | 0.231321 | 2.478071626 | 5.31E-05 | 0.00010919 |
| AC104823.1 | 0.017547794 | 4.879805269 | 8.119390076 | 2.38E-17 | 3.27E-16 |
| AC233976.1 | 0.094842249 | 0.027249173 | -1.799317445 | 1.80E-16 | 2.14E-15 |
| AC079385.1 | 0.038358208 | 0.115195317 | 1.586474845 | 0.00877708 | 0.01262482 |
| AL358115.1 | 0.156410203 | 0.432034076 | 1.465810477 | 3.71E-06 | 9.20E-06 |
| AC007216.3 | 0.089743742 | 0.404419347 | 2.171968768 | 2.11E-11 | 1.13E-10 |
| AL136366.1 | 0.092444982 | 0.027515234 | -1.748364394 | 1.49E-12 | 9.60E-12 |
| AC006116.10 | 0.097305837 | 0.044066655 | -1.142838948 | 5.65E-13 | 3.90E-12 |
| AC004837.2 | 0.129295773 | 0.735377804 | 2.507810519 | 3.42E-08 | 1.13E-07 |
| AC005841.1 | 0.073815183 | 0.340151319 | 2.204187192 | 1.64E-08 | 5.71E-08 |
| AC007601.1 | 0.006317218 | 0.051742137 | 3.033978268 | 0.03004351 | 0.03939103 |
| AL731557.1 | 0.118473097 | 0.021308472 | -2.475060445 | 3.60E-24 | 2.61E-22 |
| LINC01644 | 0.189863773 | 0.069866529 | -1.442291294 | 1.09E-12 | 7.20E-12 |
| AL122058.1 | 0.018365012 | 0.104767869 | 2.512164605 | 0.00051072 | 0.00090451 |
| IL12A-AS1 | 0.059905142 | 0.02759673 | -1.118182542 | 0.00126907 | 0.00210725 |
| AP006621.4 | 0.277040781 | 0.605882685 | 1.128940118 | 0.00081265 | 0.00139615 |
| AC016888.1 | 9.851159097 | 2.454136076 | -2.005078238 | 1.12E-16 | 1.38E-15 |
| AC092723.1 | 4.11745367 | 0.426753736 | -3.270276728 | 8.86E-24 | 5.55E-22 |
| AC011997.1 | 0.027155367 | 0.08294737 | 1.610958862 | 0.00493541 | 0.00742832 |
| ZNF341-AS1 | 0.073832781 | 0.171669779 | 1.21730268 | 2.43E-05 | 5.29E-05 |
| AL161772.1 | 0.383792253 | 0.988716221 | 1.365230911 | 5.32E-12 | 3.13E-11 |
| AC008121.2 | 0.045724155 | 0.153871517 | 1.750697788 | 0.00336098 | 0.00520518 |
| MIR122HG | 0.007830322 | 0.002743152 | -1.513237289 | 9.62E-14 | 7.33E-13 |
| HOXC-AS2 | 0.013399905 | 0.094862844 | 2.823620292 | 0.00219396 | 0.00350328 |
| AC141930.1 | 0.042516846 | 0.293827892 | 2.788864872 | 0.00050684 | 0.00089851 |
| AL021707.6 | 1.606824157 | 3.218186788 | 1.002036008 | 5.56E-07 | 1.56E-06 |
| AC007663.3 | 0.075823018 | 0.173155587 | 1.191361159 | 0.00762966 | 0.01111529 |
| LINC01687 | 0.489739852 | 0.058777551 | -3.058678442 | 2.81E-25 | 2.95E-23 |
| AC148477.3 | 0.022729008 | 0.094876888 | 2.061522005 | 0.00198306 | 0.00319458 |
| AC148476.1 | 0.171461761 | 0.511978501 | 1.57819636 | 3.36E-10 | 1.51E-09 |
| AC027288.3 | 0.041691172 | 0.12059561 | 1.532363566 | 0.02597578 | 0.03438842 |
| AC107993.1 | 0.028241873 | 0.124863075 | 2.144439223 | 0.02157521 | 0.02902062 |
| AL513542.1 | 0.440360502 | 0.107754786 | -2.030933126 | 4.58E-17 | 6.01E-16 |
| AC024884.2 | 0.01479515 | 0.067580953 | 2.191492415 | 1.22E-08 | 4.34E-08 |
| AC126768.2 | 0.102812336 | 0.046568248 | -1.142594877 | 5.16E-15 | 4.79E-14 |
| AC074117.1 | 0.963856699 | 2.011721916 | 1.061540317 | 8.92E-14 | 6.83E-13 |
| AL359513.1 | 0.441486929 | 1.082022635 | 1.293288048 | 5.25E-09 | 1.97E-08 |
| LINC01593 | 0.001345562 | 0.318892764 | 7.888718345 | 4.70E-11 | 2.41E-10 |
| AL353747.2 | 0.144167619 | 0.754303159 | 2.387397303 | 4.06E-08 | 1.34E-07 |
| AC007541.1 | 0.48180214 | 0.196236274 | -1.295849055 | 5.62E-08 | 1.81E-07 |
| AC009704.2 | 0.101578584 | 0.346428157 | 1.769959929 | 0.0001386 | 0.00026669 |
| LINC01132 | 0.071452902 | 0.255337953 | 1.83734349 | 3.13E-18 | 5.05E-17 |
| AC084864.1 | 0.053189438 | 0.235736469 | 2.147963268 | 1.13E-06 | 3.03E-06 |
| LINC01602 | 0.000928904 | 0.20712851 | 7.800780777 | 9.65E-13 | 6.45E-12 |
| AL139123.1 | 0.17019446 | 0.427392749 | 1.328378357 | 6.40E-07 | 1.78E-06 |
| AL023803.1 | 0.17150202 | 0.473097 | 1.463910447 | 0.00670172 | 0.00983966 |
| VLDLR-AS1 | 0.176010002 | 0.059798978 | -1.55746469 | 6.69E-10 | 2.87E-09 |
| AC092171.1 | 0.104701289 | 0.480593533 | 2.198538034 | 5.87E-12 | 3.44E-11 |
| PTOV1-AS2 | 1.283228418 | 2.738677169 | 1.093701216 | 8.63E-11 | 4.26E-10 |
| LINC00114 | 0.24664557 | 0.776874462 | 1.655242113 | 1.13E-05 | 2.59E-05 |
| AC137894.1 | 0.044759692 | 0.268372239 | 2.583963439 | 3.15E-05 | 6.69E-05 |
| AC068870.1 | 0.011654304 | 0.028501068 | 1.290153096 | 0.02031887 | 0.02743903 |
| ZNF451-AS1 | 0.060217778 | 0.201274087 | 1.74090008 | 0.00255643 | 0.00404532 |
| NEBL-AS1 | 0.155290238 | 0.332041255 | 1.096395364 | 2.60E-07 | 7.63E-07 |
| AC023794.2 | 0.053503244 | 0.025283831 | -1.081411273 | 1.86E-08 | 6.44E-08 |
| AC091182.2 | 0.06493338 | 0.728536377 | 3.487968785 | 8.73E-17 | 1.10E-15 |
| LINC02287 | 0.030678399 | 0.014213868 | -1.10992404 | 3.29E-12 | 2.01E-11 |
| Z83843.1 | 0.615517272 | 3.47677937 | 2.497880274 | 4.36E-05 | 9.10E-05 |
| AC120042.2 | 0.020979223 | 0.191269418 | 3.188573093 | 1.18E-05 | 2.69E-05 |
| AC126773.1 | 0.018780344 | 0.070008949 | 1.89831589 | 0.00969847 | 0.01383868 |
| PLCG1-AS1 | 0.038144371 | 0.206055678 | 2.433492158 | 3.81E-09 | 1.46E-08 |
| IGF2-AS | 0.008223694 | 0.123769888 | 3.911730023 | 0.00024711 | 0.00045864 |
| FAM83C-AS1 | 0.05295595 | 0.257424194 | 2.281282959 | 7.11E-08 | 2.26E-07 |
| AC124067.2 | 0.546104202 | 6.473841428 | 3.567373866 | 3.47E-21 | 1.11E-19 |
| AL135905.1 | 2.411777879 | 0.835785714 | -1.528892039 | 4.86E-20 | 1.17E-18 |
| AC243772.2 | 0.00714593 | 0.036165286 | 2.339411875 | 6.56E-06 | 1.56E-05 |
| AC007249.1 | 0.194082084 | 0.488849337 | 1.332722947 | 1.49E-08 | 5.22E-08 |
| AC004593.1 | 0.032150256 | 0.767204442 | 4.576708946 | 2.54E-12 | 1.57E-11 |
| UNQ6494 | 0.04213348 | 0.104508582 | 1.31058243 | 2.75E-06 | 6.96E-06 |
| AC092171.4 | 0.314713214 | 0.843656684 | 1.422618274 | 4.66E-09 | 1.76E-08 |
| AL161781.2 | 0.098985401 | 0.020511992 | -2.270748132 | 3.88E-14 | 3.11E-13 |
| LINC00106 | 0.891719059 | 2.294282038 | 1.363381595 | 6.25E-08 | 1.99E-07 |
| AC008083.1 | 0.021852649 | 0.209506237 | 3.261113119 | 1.73E-08 | 6.01E-08 |
| SNHG7 | 3.863125082 | 10.23282591 | 1.405364321 | 4.93E-18 | 7.65E-17 |
| LINC01894 | 0.063281427 | 0.020302465 | -1.640127267 | 3.65E-09 | 1.41E-08 |
| AC026368.1 | 0.073144756 | 0.642134341 | 3.134048805 | 1.67E-15 | 1.69E-14 |
| AC073326.1 | 0.017831696 | 0.23252599 | 3.704876158 | 8.38E-06 | 1.96E-05 |
| AF131216.3 | 0.022255047 | 0.005496194 | -2.017627623 | 3.09E-17 | 4.18E-16 |
| LINC01629 | 0.085456549 | 0.041409766 | -1.045220014 | 0.00028416 | 0.00052295 |
| RAET1E-AS1 | 0.060934057 | 0.303612021 | 2.316908217 | 1.10E-07 | 3.41E-07 |
| LINC02560 | 0.02734636 | 0.244644974 | 3.161268923 | 1.16E-06 | 3.08E-06 |
| GRIK1-AS1 | 0.080399979 | 0.024698621 | -1.702764608 | 1.71E-13 | 1.25E-12 |
| AC129507.1 | 0.309283038 | 0.142049747 | -1.122531457 | 3.39E-14 | 2.75E-13 |
| MIR3150BHG | 0.485344053 | 0.101767347 | -2.253733087 | 1.35E-15 | 1.39E-14 |
| AC127024.2 | 0.034610313 | 0.283008119 | 3.031569552 | 1.78E-08 | 6.16E-08 |
| AC004947.1 | 0.20823779 | 0.026990326 | -2.947717579 | 3.42E-24 | 2.52E-22 |
| AC134312.1 | 0.112964795 | 0.030201717 | -1.903170739 | 1.66E-18 | 2.81E-17 |
| MIR17HG | 0.157807249 | 1.42982472 | 3.179602919 | 4.79E-19 | 9.07E-18 |
| AC011306.1 | 0.17064568 | 0.022416344 | -2.928381004 | 4.24E-12 | 2.56E-11 |
| AC112484.1 | 0.128500392 | 0.258692911 | 1.009467759 | 2.49E-05 | 5.40E-05 |
| AC092718.5 | 0.107863142 | 0.293003797 | 1.441717404 | 9.43E-09 | 3.41E-08 |
| AC005670.1 | 0.022858105 | 0.107326404 | 2.231227335 | 1.73E-05 | 3.86E-05 |
| LINC01133 | 31.08331905 | 10.44951728 | -1.572704264 | 2.43E-18 | 4.00E-17 |
| SNHG15 | 2.198050072 | 8.432433854 | 1.939724845 | 3.74E-25 | 3.71E-23 |
| AP003390.1 | 0.599363732 | 1.400358331 | 1.224292346 | 2.44E-06 | 6.21E-06 |
| AC005332.1 | 0.043865879 | 0.098396043 | 1.165501139 | 0.00070419 | 0.00122253 |
| MCPH1-AS1 | 0.388643787 | 0.168849704 | -1.202708802 | 3.25E-18 | 5.22E-17 |
| LINC01348 | 0.091089445 | 0.421464424 | 2.210055058 | 2.02E-10 | 9.40E-10 |
| AC104458.1 | 0.036826646 | 0.017271825 | -1.092329427 | 1.52E-07 | 4.63E-07 |
| DSCR9 | 0.023747232 | 0.087798695 | 1.886440117 | 4.50E-10 | 1.98E-09 |
| AL929236.1 | 0.028947506 | 0.122542592 | 2.081772312 | 0.00042928 | 0.00077128 |
| AC118344.1 | 0.102818878 | 0.260157046 | 1.339277602 | 0.01012019 | 0.01440873 |
| AC009996.1 | 0.131424152 | 0.516888671 | 1.975623158 | 7.14E-08 | 2.27E-07 |
| AC025035.1 | 0.046706857 | 0.017119545 | -1.447989987 | 1.64E-09 | 6.59E-09 |
| AP005233.2 | 0.180155699 | 2.665080847 | 3.886863104 | 7.49E-16 | 8.04E-15 |
| CASC11 | 0.014038078 | 0.046905238 | 1.740403611 | 6.87E-10 | 2.94E-09 |
| LINC02595 | 0.047313634 | 1.040667504 | 4.459109402 | 1.01E-19 | 2.21E-18 |
| AC004637.1 | 0.367545701 | 0.138048352 | -1.412749984 | 1.37E-11 | 7.63E-11 |
| AC090197.1 | 0.024206634 | 0.114568459 | 2.242735555 | 1.62E-06 | 4.24E-06 |
| AC093585.1 | 0.168993404 | 0.549130984 | 1.700183377 | 0.00600184 | 0.00891287 |
| AC008434.1 | 0.079510924 | 0.191346031 | 1.266958987 | 0.00044671 | 0.0008005 |
| LINC02563 | 0.045169845 | 0.91194932 | 4.335521774 | 4.81E-07 | 1.36E-06 |
| AC097641.1 | 0.052887294 | 0.023024156 | -1.199772875 | 3.68E-16 | 4.15E-15 |
| LINC00525 | 0.172167097 | 0.634602509 | 1.882043767 | 1.45E-08 | 5.10E-08 |
| AC007639.1 | 0.062487139 | 0.362632308 | 2.536876274 | 1.06E-09 | 4.39E-09 |
| AJ009632.2 | 0.020723277 | 0.051169553 | 1.304033468 | 0.02421329 | 0.03228056 |
| HIF1A-AS2 | 0.183174763 | 1.922161229 | 3.391436698 | 0.00428522 | 0.00652718 |
| LINC00582 | 0.762766188 | 0.165762258 | -2.202125334 | 8.64E-18 | 1.30E-16 |
| AP000439.1 | 0.030657065 | 0.011491093 | -1.415703578 | 8.18E-12 | 4.68E-11 |
| AC010401.1 | 0.006332223 | 0.022713987 | 1.842796945 | 0.00127893 | 0.00212299 |
| AC008742.1 | 0.009996539 | 0.083726172 | 3.06617802 | 0.00033062 | 0.00060419 |
| LINC00524 | 0.015338609 | 0.253803617 | 4.0484731 | 2.64E-06 | 6.70E-06 |
| LINC02038 | 4.192288643 | 1.159408905 | -1.85434858 | 1.09E-20 | 3.08E-19 |
| AL161729.4 | 0.16439351 | 0.780221152 | 2.24672977 | 5.32E-12 | 3.13E-11 |
| AC022613.2 | 0.327390235 | 0.157965467 | -1.051402085 | 9.75E-17 | 1.22E-15 |
| AC068790.5 | 0.154574632 | 0.533805671 | 1.78801106 | 0.00045376 | 0.00081206 |
| DLX6-AS1 | 0.004770543 | 0.10826868 | 4.504318567 | 0.0001591 | 0.00030335 |
| JAZF1-AS1 | 0.052227226 | 0.010343468 | -2.336082116 | 1.53E-15 | 1.56E-14 |
| AC084026.2 | 0.023419426 | 0.091116041 | 1.959999357 | 0.00274053 | 0.00431163 |
| AC006238.1 | 0.160131947 | 0.063292013 | -1.339165801 | 1.48E-11 | 8.21E-11 |
| AC078850.1 | 0.022134348 | 0.063200354 | 1.513645759 | 0.00212409 | 0.00340467 |
| AL138760.1 | 0.021199708 | 0.158599175 | 2.903268998 | 1.34E-06 | 3.53E-06 |
| AC100827.4 | 0.007763463 | 0.032830018 | 2.080243389 | 0.03718285 | 0.04797873 |
| AP000851.1 | 0.006189699 | 0.360603184 | 5.864399042 | 5.75E-14 | 4.52E-13 |
| AC020658.4 | 0.04971525 | 0.15367638 | 1.628135083 | 5.75E-06 | 1.38E-05 |
| AC015468.3 | 0.045791186 | 0.011805939 | -1.955557091 | 5.13E-12 | 3.04E-11 |
| AC104958.2 | 0.588363618 | 4.36088508 | 2.889841028 | 3.70E-20 | 9.18E-19 |
| TRG-AS1 | 0.508336443 | 0.246185139 | -1.046039986 | 5.92E-12 | 3.47E-11 |
| AC007128.1 | 0.017840389 | 0.623272804 | 5.12664476 | 4.12E-22 | 1.68E-20 |
| AC005363.2 | 0.016988913 | 0.175593693 | 3.369575606 | 4.89E-08 | 1.59E-07 |
| AC064805.2 | 0.029581314 | 0.011985447 | -1.30340241 | 1.00E-12 | 6.67E-12 |
| LINC02389 | 0.005579179 | 0.021437214 | 1.941992737 | 0.00253064 | 0.00400801 |
| PTENP1-AS | 0.076425082 | 0.018618033 | -2.037345554 | 3.41E-20 | 8.53E-19 |
| LINC02266 | 0.000613675 | 0.08834418 | 7.169517226 | 2.41E-09 | 9.51E-09 |
| AC055811.1 | 0.03073896 | 0.154848032 | 2.332712778 | 0.00335087 | 0.005191 |
| AC090825.1 | 0.267994425 | 0.102830567 | -1.381933812 | 8.25E-14 | 6.39E-13 |
| C5orf64 | 0.016973653 | 0.007851861 | -1.112190572 | 3.18E-10 | 1.44E-09 |
| AC105935.2 | 0.039184136 | 0.140149777 | 1.838627874 | 0.00016241 | 0.00030922 |
| SH3TC2-DT | 0.006251086 | 0.061433558 | 3.29684815 | 5.39E-12 | 3.17E-11 |
| AC010973.2 | 0.269372822 | 0.861289303 | 1.676893614 | 1.08E-15 | 1.13E-14 |
| AL442638.1 | 0.091470376 | 0.037470621 | -1.287544673 | 4.06E-17 | 5.40E-16 |
| AC105398.1 | 0.076359903 | 0.008968649 | -3.089852737 | 3.34E-25 | 3.37E-23 |
| AL033527.3 | 0.086959437 | 0.363691434 | 2.064300447 | 3.10E-15 | 2.98E-14 |
| AC005674.1 | 0.558874041 | 0.16624191 | -1.749239032 | 1.70E-15 | 1.72E-14 |
| LINC02185 | 0.197400177 | 0.032315892 | -2.610807566 | 1.05E-23 | 6.51E-22 |
| AC104365.1 | 0.008510809 | 0.413861339 | 5.603707334 | 3.67E-07 | 1.06E-06 |
| AC104791.1 | 0.0195724 | 0.135860748 | 2.795236111 | 1.74E-05 | 3.87E-05 |
| STAG3L5P-PVRIG2P-PILRB | 0.279961598 | 0.708892717 | 1.34033836 | 1.20E-11 | 6.72E-11 |
| LINC02416 | 0.046258958 | 0.141475558 | 1.612748162 | 0.00045814 | 0.00081964 |
| AP003396.3 | 0.050977951 | 0.014793685 | -1.78489198 | 7.28E-16 | 7.85E-15 |
| AL355112.1 | 0.005081874 | 0.084829868 | 4.061139786 | 9.97E-08 | 3.10E-07 |
| MYCNOS | 0.010970055 | 0.048227935 | 2.136298274 | 0.00074511 | 0.00128782 |
| AC084876.1 | 0.077639082 | 0.176368482 | 1.183737809 | 0.00129887 | 0.00215281 |
| AC008610.1 | 0.798199749 | 1.764051585 | 1.144071019 | 1.08E-08 | 3.87E-08 |
| AP001412.1 | 0.126731254 | 0.298517639 | 1.236043822 | 5.48E-07 | 1.54E-06 |
| INE1 | 0.863148279 | 1.732633271 | 1.005286002 | 1.11E-05 | 2.54E-05 |
| AL590004.3 | 0.005391614 | 0.136362558 | 4.660586588 | 1.53E-17 | 2.18E-16 |
| AL121820.2 | 0.2105439 | 0.59073049 | 1.488378998 | 8.47E-07 | 2.30E-06 |
| AL356652.1 | 0.02837515 | 0.230980155 | 3.025068969 | 1.36E-11 | 7.56E-11 |
| AC010719.1 | 0.684743158 | 3.466083466 | 2.339671545 | 1.37E-16 | 1.66E-15 |
| AC069222.1 | 0.020271188 | 0.130055738 | 2.681627505 | 8.24E-13 | 5.55E-12 |
| AC002306.1 | 0.020459496 | 0.113447183 | 2.471178268 | 0.00876649 | 0.01261643 |
| LINC00954 | 0.088531902 | 0.228703329 | 1.369208038 | 0.01166136 | 0.01644411 |
| ERICD | 0.128567486 | 0.311750772 | 1.277867292 | 1.54E-13 | 1.13E-12 |
| AC074044.1 | 0.040645752 | 0.126438432 | 1.637258574 | 8.34E-06 | 1.95E-05 |
| AC004839.1 | 0.042451294 | 0.107631289 | 1.342217099 | 0.00142646 | 0.00235426 |
| AF117829.1 | 0.208630725 | 0.663883357 | 1.669978147 | 1.05E-12 | 6.94E-12 |
| AC144831.1 | 0.936002833 | 0.260477873 | -1.845352074 | 1.71E-20 | 4.60E-19 |
| AL021707.5 | 0.068601538 | 0.202287119 | 1.560091641 | 0.00126423 | 0.00209986 |
| AC026369.1 | 0.00517043 | 0.014828128 | 1.519980376 | 0.00901268 | 0.01295024 |
| AC087311.2 | 0.069213818 | 0.014000339 | -2.30559836 | 8.49E-16 | 9.04E-15 |
| AC096888.1 | 0.023086252 | 0.117835226 | 2.351665035 | 0.00090878 | 0.00154573 |
| TRDN-AS1 | 0.01143943 | 0.003199864 | -1.837932875 | 5.54E-21 | 1.70E-19 |
| ZNF252P-AS1 | 0.095917081 | 0.229989355 | 1.261707434 | 3.55E-07 | 1.03E-06 |
| AL117379.1 | 0.477144293 | 1.629264701 | 1.77172349 | 3.41E-10 | 1.53E-09 |
| AL445483.1 | 0.075199766 | 0.174497848 | 1.21440917 | 0.008522 | 0.01227753 |
| AL136172.1 | 0.009479526 | 0.037373575 | 1.979131731 | 2.84E-05 | 6.08E-05 |
| BX470102.1 | 0.915102662 | 5.45645785 | 2.575959197 | 2.15E-20 | 5.63E-19 |
| AC023421.1 | 1.269730338 | 0.281710725 | -2.172235738 | 2.55E-06 | 6.48E-06 |
| AC131212.1 | 0.051545248 | 0.127662132 | 1.3084193 | 0.00048269 | 0.00085979 |
| AL117381.1 | 0.087877882 | 1.025271 | 3.544361382 | 0.02855952 | 0.03760808 |
| AC092338.1 | 0.134231162 | 0.628867949 | 2.228037476 | 0.02489893 | 0.03307524 |
| AC104564.4 | 0.01882929 | 0.222727714 | 3.564230577 | 3.40E-09 | 1.31E-08 |
| AC093627.7 | 0.069199506 | 0.023283017 | -1.571483745 | 4.30E-13 | 3.02E-12 |
| AL137782.1 | 0.240372681 | 0.818514011 | 1.767734175 | 1.05E-13 | 7.96E-13 |
| AC093752.2 | 0.023837103 | 0.12137195 | 2.348154208 | 0.00474126 | 0.00717373 |
| AP002387.1 | 2.9332008 | 6.450162033 | 1.136859564 | 3.28E-08 | 1.09E-07 |
| AC009336.1 | 0.156304342 | 0.050690104 | -1.624581839 | 6.24E-14 | 4.88E-13 |
| LINC01060 | 0.001423946 | 0.026435424 | 4.214505767 | 3.04E-06 | 7.64E-06 |
| AL160408.3 | 0.005539743 | 0.020750589 | 1.905261342 | 0.00646061 | 0.00953959 |
| AC099850.3 | 3.225716456 | 9.051678605 | 1.488565731 | 5.72E-19 | 1.06E-17 |
| AL592071.1 | 0.038598192 | 0.094536697 | 1.292341179 | 0.00219112 | 0.00349977 |
| AL359694.2 | 0.007422224 | 0.037417384 | 2.33378517 | 0.00017297 | 0.00032772 |
| AL512274.1 | 0.582787494 | 1.308074907 | 1.166403334 | 7.01E-07 | 1.94E-06 |
| RNF219-AS1 | 0.00486626 | 0.014358765 | 1.561046361 | 0.00327872 | 0.00509225 |
| AC245100.7 | 0.188106906 | 0.632980234 | 1.750607631 | 8.07E-07 | 2.20E-06 |
| AL596223.2 | 0.121088698 | 0.564424898 | 2.220717419 | 5.97E-11 | 3.01E-10 |
| AL606834.1 | 0.370179899 | 0.817185797 | 1.142437573 | 2.29E-05 | 5.02E-05 |
| Z82243.1 | 0.190426283 | 0.916761655 | 2.267314086 | 7.09E-12 | 4.09E-11 |
| LINC02418 | 0.006824177 | 4.285042812 | 9.294438935 | 9.20E-20 | 2.04E-18 |
| AC107959.3 | 0.070196282 | 0.664314956 | 3.242400864 | 3.07E-19 | 6.09E-18 |
| AC004816.2 | 0.116331265 | 0.259238016 | 1.156038415 | 0.00040958 | 0.00073711 |
| AC015689.1 | 0.131516054 | 0.324015116 | 1.3008222 | 7.66E-05 | 0.00015347 |
| AC015912.3 | 1.41727781 | 3.107834449 | 1.132787077 | 9.44E-09 | 3.41E-08 |
| AL365181.3 | 4.571831656 | 11.20565993 | 1.293383428 | 1.18E-13 | 8.87E-13 |
| H19 | 1.226784421 | 18.9259648 | 3.947413191 | 1.81E-11 | 9.84E-11 |
| AC117382.2 | 0.004017994 | 0.014473224 | 1.848839035 | 0.02317821 | 0.03101614 |
| LINC01234 | 0.004358941 | 1.014206422 | 7.862158036 | 3.59E-16 | 4.06E-15 |
| MGC32805 | 0.523617246 | 1.122353952 | 1.099943205 | 0.0003859 | 0.0006984 |
| AC105339.2 | 0.054511632 | 0.167765023 | 1.62180593 | 1.82E-09 | 7.27E-09 |
| AC018682.1 | 0.070332648 | 0.385187382 | 2.453294008 | 0.01535626 | 0.02120479 |
| AL390037.1 | 0.031803222 | 0.129150919 | 2.021813049 | 0.00057234 | 0.00100643 |
| LINC00887 | 0.006622907 | 0.038840129 | 2.552011507 | 7.28E-08 | 2.31E-07 |
| AC018809.1 | 0.226766929 | 0.581761357 | 1.359217209 | 6.36E-06 | 1.51E-05 |
| AC026333.4 | 0.231965245 | 0.5254284 | 1.179585518 | 1.33E-06 | 3.52E-06 |
| DLEU2 | 0.342306366 | 1.2389737 | 1.855785536 | 5.58E-13 | 3.86E-12 |
| KLHL7-DT | 0.098076948 | 0.220457067 | 1.168511728 | 0.00170902 | 0.00278769 |
| ODF2-AS1 | 0.062908731 | 0.258987192 | 2.041548583 | 7.02E-05 | 0.00014151 |
| RNASEH2B-AS1 | 0.040383382 | 0.148399859 | 1.877656061 | 3.85E-07 | 1.10E-06 |
| AP000785.1 | 0.064599441 | 0.460853729 | 2.834715338 | 0.00072398 | 0.00125488 |
| LINC01855 | 0.112793838 | 0.005695944 | -4.307609414 | 6.03E-28 | 1.06E-25 |
| TBL1XR1-AS1 | 0.010775458 | 0.333929952 | 4.953724341 | 7.32E-06 | 1.73E-05 |
| LNCTAM34A | 0.190121409 | 0.558738233 | 1.555251544 | 4.33E-11 | 2.23E-10 |
| LINC02280 | 0.01480555 | 0.039876941 | 1.429416624 | 0.00381717 | 0.00586174 |
| LINC02023 | 1.089774214 | 0.086154656 | -3.660956685 | 8.17E-23 | 3.91E-21 |
| LINC02487 | 0.307977002 | 1.167392158 | 1.922394756 | 7.57E-12 | 4.34E-11 |
| AP001043.1 | 0.11977358 | 0.550251924 | 2.199782579 | 2.97E-08 | 9.94E-08 |
| AC107959.1 | 0.363086051 | 0.136682174 | -1.40948641 | 1.12E-16 | 1.38E-15 |
| AC024651.1 | 0.129670845 | 0.019313741 | -2.747154581 | 2.65E-13 | 1.89E-12 |
| AC010319.4 | 0.220856987 | 0.513713784 | 1.217852309 | 1.59E-11 | 8.75E-11 |
| AC026904.1 | 0.068478461 | 0.025390862 | -1.431340894 | 3.69E-07 | 1.06E-06 |
| AL139339.1 | 0.159762381 | 0.068059329 | -1.231062897 | 1.11E-10 | 5.36E-10 |
| AL670729.3 | 0.030155792 | 0.067346623 | 1.159170479 | 0.03131271 | 0.04090757 |
| AC105460.1 | 0.01072643 | 4.424787667 | 8.688294356 | 3.57E-05 | 7.55E-05 |
| AC020891.2 | 0.03670374 | 0.275053589 | 2.905713739 | 1.49E-12 | 9.56E-12 |
| AC011365.1 | 0.271600279 | 0.067166692 | -2.015667081 | 1.48E-18 | 2.51E-17 |
| AC023794.1 | 0.191943556 | 0.092668479 | -1.05053153 | 1.34E-15 | 1.38E-14 |
| AP001527.2 | 0.154976052 | 0.467592204 | 1.59320558 | 1.70E-11 | 9.26E-11 |
| AC104809.2 | 0.020849644 | 0.287906735 | 3.787506853 | 0.00020325 | 0.00038152 |
| FLJ16779 | 0.014198996 | 0.111589695 | 2.974342994 | 2.48E-08 | 8.38E-08 |
| LINC00621 | 0.069707257 | 0.017704183 | -1.977218616 | 3.02E-15 | 2.93E-14 |
| AL031717.1 | 0.181780958 | 0.423573294 | 1.22041055 | 0.0004941 | 0.00087764 |
| AL645608.8 | 0.009820173 | 0.077690299 | 2.98391417 | 1.33E-05 | 3.01E-05 |
| LINC02198 | 0.016773132 | 0.061686458 | 1.87880171 | 0.00087543 | 0.00149553 |
| AC009502.1 | 0.001476273 | 0.019718585 | 3.739524248 | 5.98E-06 | 1.43E-05 |
| HSD52 | 0.019327584 | 0.166150095 | 3.103753908 | 0.00909785 | 0.01305885 |
| CASC15 | 0.101085442 | 0.407202623 | 2.010171621 | 5.33E-11 | 2.71E-10 |
| AC124016.1 | 0.146364724 | 0.344480099 | 1.234852757 | 1.48E-10 | 7.08E-10 |
| LINC01160 | 0.23581763 | 0.104921527 | -1.168360861 | 3.70E-06 | 9.18E-06 |
| AC068768.2 | 0.065166224 | 0.166757849 | 1.355558358 | 0.02476915 | 0.03291916 |
| AC007938.3 | 0.276721713 | 0.799011882 | 1.529781109 | 4.25E-10 | 1.88E-09 |
| U47924.1 | 0.121456291 | 0.415659555 | 1.774965158 | 4.60E-09 | 1.74E-08 |
| AC005342.2 | 0.134129776 | 0.027427227 | -2.289948888 | 4.06E-15 | 3.83E-14 |
| AL451050.2 | 0.27530969 | 0.617427088 | 1.165213387 | 4.32E-08 | 1.41E-07 |
| AC012184.3 | 0.235202919 | 0.489012601 | 1.055965674 | 1.28E-07 | 3.93E-07 |
| LINC00239 | 1.222472645 | 3.060575993 | 1.324001007 | 1.38E-07 | 4.21E-07 |
| FEZF1-AS1 | 0.002211203 | 1.592762213 | 9.492483404 | 1.01E-21 | 3.65E-20 |
| AC022034.3 | 0.249091589 | 0.051875815 | -2.263542308 | 6.23E-14 | 4.88E-13 |
| JARID2-AS1 | 0.118256569 | 0.376621856 | 1.671196401 | 0.00021435 | 0.00040098 |
| AL136115.1 | 0.051438876 | 0.489873644 | 3.251478646 | 0.00231207 | 0.00368324 |
| AC009065.1 | 0.00889687 | 0.045616171 | 2.358175611 | 1.70E-05 | 3.80E-05 |
| LINC00299 | 0.03382811 | 0.082393637 | 1.284310355 | 0.00017904 | 0.00033852 |
| AL133299.1 | 0.040730804 | 0.192004645 | 2.236949026 | 8.42E-07 | 2.29E-06 |
| FGF14-AS2 | 1.082603797 | 0.301966123 | -1.842046743 | 1.18E-19 | 2.55E-18 |
| AC010547.2 | 0.011643062 | 0.781129899 | 6.068020089 | 5.23E-07 | 1.47E-06 |
| BANCR | 0.029197157 | 0.108026318 | 1.887483041 | 0.00128095 | 0.00212569 |
| EGOT | 0.01799451 | 0.160812362 | 3.159749599 | 1.56E-08 | 5.46E-08 |
| AC005726.2 | 0.023029854 | 0.148022049 | 2.68423494 | 4.58E-08 | 1.49E-07 |
| MEF2C-AS1 | 0.140701842 | 0.028765914 | -2.290208971 | 1.12E-21 | 3.99E-20 |
| CASC8 | 0.296229327 | 1.176193062 | 1.989338506 | 7.06E-17 | 9.06E-16 |
| MNX1-AS1 | 1.072486212 | 4.27349523 | 1.994457411 | 5.33E-20 | 1.27E-18 |
| MBNL1-AS1 | 2.8156113 | 0.530347685 | -2.408437805 | 1.79E-19 | 3.73E-18 |
| AC090150.1 | 0.121783348 | 0.052343509 | -1.218234322 | 2.53E-09 | 9.95E-09 |
| AP000487.1 | 0.12394908 | 0.32265254 | 1.380233822 | 6.73E-16 | 7.30E-15 |
| ALG13-AS1 | 0.337670635 | 1.092732693 | 1.694251899 | 0.03818407 | 0.04917545 |
| AL353708.3 | 0.145668153 | 0.399115178 | 1.454119647 | 3.34E-10 | 1.50E-09 |
| AC211433.1 | 0.022103396 | 0.324470774 | 3.875748574 | 1.18E-07 | 3.65E-07 |
| PDCD4-AS1 | 4.265295819 | 1.713467579 | -1.315726908 | 6.96E-22 | 2.60E-20 |
| LINC01146 | 0.02887273 | 0.174655716 | 2.596734408 | 1.78E-07 | 5.34E-07 |
| AC083809.1 | 0.183219641 | 1.447533406 | 2.981950573 | 1.06E-10 | 5.12E-10 |
| LINC01625 | 0.034515578 | 0.009158575 | -1.914052649 | 2.96E-14 | 2.42E-13 |
| AC141002.1 | 0.253238957 | 0.557652702 | 1.138867553 | 0.00032125 | 0.00058765 |
| AP001001.1 | 0.071998913 | 0.195660118 | 1.442302682 | 0.00972607 | 0.01387294 |
| AL158055.1 | 0.085461601 | 0.018592529 | -2.200553315 | 5.17E-28 | 9.39E-26 |
| AL096677.1 | 0.032019742 | 0.137117544 | 2.098379601 | 4.14E-09 | 1.57E-08 |
| AC078923.1 | 0.004940465 | 0.253055197 | 5.678661474 | 4.80E-18 | 7.50E-17 |
| AL355102.4 | 0.283637553 | 0.12604338 | -1.170128212 | 1.14E-14 | 9.92E-14 |
| AC011773.1 | 0.030574594 | 0.215014355 | 2.814027734 | 1.69E-08 | 5.89E-08 |
| AC092828.1 | 0.024098374 | 0.318487163 | 3.724227517 | 0.02536814 | 0.03364934 |
| KRT7-AS | 0.010522484 | 0.182711649 | 4.118021464 | 8.04E-17 | 1.02E-15 |
| LINC02073 | 0.007932242 | 0.032538062 | 2.036327771 | 1.29E-06 | 3.42E-06 |
| AC092849.1 | 0.161450017 | 0.380535896 | 1.236944951 | 8.47E-06 | 1.98E-05 |
| LINC01752 | 9.941644129 | 2.290010814 | -2.11813005 | 4.26E-20 | 1.04E-18 |
| LINC02332 | 0.033548253 | 0.15537981 | 2.211489511 | 0.00017011 | 0.00032286 |
| LINC01624 | 0.077863129 | 0.018376209 | -2.083101167 | 3.28E-20 | 8.25E-19 |
| LINC02473 | 0.073501174 | 0.245328707 | 1.73887686 | 0.00407851 | 0.00622973 |
| GS1-124K5.4 | 0.858589932 | 2.287778621 | 1.413906295 | 5.43E-15 | 5.00E-14 |
| AC025754.2 | 0.027951351 | 0.069809013 | 1.320495276 | 0.01575322 | 0.02170346 |
| AF111167.2 | 0.485343502 | 0.159037245 | -1.609641502 | 2.52E-24 | 1.99E-22 |
| DLGAP1-AS2 | 0.692356615 | 3.583798482 | 2.371902288 | 2.70E-23 | 1.52E-21 |
| AL009178.2 | 0.204572648 | 0.453759601 | 1.149314902 | 0.00015306 | 0.00029234 |
| LINC01018 | 0.149482776 | 0.053324603 | -1.487106038 | 3.73E-11 | 1.95E-10 |
| AC108156.1 | 0.092257842 | 0.028064833 | -1.716908059 | 3.83E-19 | 7.41E-18 |
| AC012507.2 | 0.047659906 | 0.014985143 | -1.669243239 | 8.54E-17 | 1.08E-15 |
| AC002511.1 | 0.54892906 | 0.170323932 | -1.688338557 | 1.26E-15 | 1.31E-14 |
| AP000525.1 | 0.029066257 | 0.299896776 | 3.367048802 | 7.55E-07 | 2.07E-06 |
| NPSR1-AS1 | 0.001041339 | 0.187349494 | 7.491149012 | 3.14E-19 | 6.21E-18 |
| AC005393.1 | 0.180754685 | 0.405250474 | 1.164780834 | 4.87E-05 | 0.00010079 |
| AC022217.3 | 0.242499862 | 0.065203145 | -1.894970457 | 2.33E-17 | 3.21E-16 |
| AL157895.1 | 0.221753803 | 0.078452429 | -1.499068819 | 6.18E-15 | 5.64E-14 |
| LINC00174 | 0.536191006 | 1.191296606 | 1.15171373 | 1.23E-10 | 5.93E-10 |
| AC093620.1 | 0.421372575 | 1.436259238 | 1.769147847 | 2.75E-12 | 1.70E-11 |
| AC015722.2 | 0.078283281 | 0.027271949 | -1.521286398 | 5.32E-12 | 3.13E-11 |
| LINC02432 | 0.022282791 | 0.629348433 | 4.819857128 | 0.00210632 | 0.00337916 |
| NKILA | 0.342941527 | 1.285272646 | 1.906039915 | 2.00E-11 | 1.08E-10 |
| AL031705.1 | 0.073383945 | 0.192434608 | 1.390831922 | 4.74E-05 | 9.86E-05 |
| AC018648.1 | 0.097073317 | 0.236941079 | 1.287381656 | 0.0017603 | 0.00286534 |
| AL031722.1 | 0.655429793 | 0.327139428 | -1.002535606 | 6.03E-10 | 2.61E-09 |
| AC016405.1 | 0.024579051 | 0.28445516 | 3.532700151 | 0.03082311 | 0.04034528 |
| LINC01080 | 0.101811518 | 0.02093581 | -2.281856162 | 3.48E-22 | 1.44E-20 |
| AL109945.1 | 0.012970912 | 0.055062766 | 2.085797141 | 0.00024909 | 0.00046168 |
| AC116552.1 | 0.063385944 | 0.266250767 | 2.070550818 | 3.70E-08 | 1.22E-07 |
| AC022733.2 | 0.185690477 | 0.045991188 | -2.013470469 | 2.94E-11 | 1.55E-10 |
| AL583824.1 | 0.012532346 | 0.095691953 | 2.932741098 | 0.02448401 | 0.03260251 |
| AC026688.2 | 0.022773896 | 0.009335466 | -1.286587223 | 6.00E-10 | 2.60E-09 |
| AC067930.5 | 0.103820026 | 0.534719646 | 2.364697928 | 1.21E-11 | 6.78E-11 |
| AL390198.1 | 0.946519283 | 2.589635378 | 1.452045178 | 0.00233924 | 0.00372217 |
| LINC01289 | 0.033914453 | 0.00560285 | -2.597667485 | 5.47E-25 | 5.06E-23 |
| AC106886.3 | 0.078254333 | 0.213238384 | 1.44622462 | 4.45E-05 | 9.27E-05 |
| AL158839.1 | 0.057485602 | 0.223282671 | 1.95759872 | 0.00927326 | 0.01327221 |
| LINC01252 | 0.057108636 | 0.14564181 | 1.350643751 | 0.00011664 | 0.00022666 |
| AL445183.2 | 0.017061823 | 0.09819996 | 2.524950673 | 6.67E-07 | 1.85E-06 |
| AC109361.1 | 0.007482581 | 0.023105732 | 1.626642833 | 0.02328802 | 0.03115544 |
| AC134312.3 | 0.212467428 | 0.048961612 | -2.117518741 | 8.14E-20 | 1.83E-18 |
| AC020913.2 | 0.020606187 | 0.062961861 | 1.61140057 | 0.00453035 | 0.00687176 |
| AC012213.2 | 0.003668275 | 0.022939093 | 2.644634475 | 1.60E-05 | 3.59E-05 |
| AL121829.2 | 0.112528088 | 0.59473405 | 2.401959517 | 1.12E-09 | 4.64E-09 |
| LINC-PINT | 0.287557705 | 1.282812734 | 2.157387178 | 8.75E-18 | 1.31E-16 |
| AC020978.7 | 0.076516453 | 0.168675288 | 1.140406719 | 0.00016278 | 0.00030981 |
| AC113383.1 | 0.589341245 | 0.129207072 | -2.189418198 | 4.03E-22 | 1.65E-20 |
| AL021328.1 | 0.008780174 | 0.153829517 | 4.130938936 | 0.00804274 | 0.01164244 |
| SATB2-AS1 | 5.90169831 | 2.877276945 | -1.036426082 | 5.97E-12 | 3.49E-11 |
| AC005062.1 | 0.101932092 | 0.290434481 | 1.510604408 | 2.98E-05 | 6.36E-05 |
| AL162724.2 | 0.205176462 | 0.732718607 | 1.83639402 | 2.65E-05 | 5.72E-05 |
| AL049646.1 | 0.005667169 | 0.038336129 | 2.758004619 | 0.02572612 | 0.03407445 |
| LINC00390 | 0.560314805 | 0.190000973 | -1.560230809 | 5.14E-13 | 3.58E-12 |
| AL162742.1 | 0.022121354 | 0.23569507 | 3.413409992 | 3.55E-07 | 1.02E-06 |
| CRAT37 | 0.004429749 | 0.268107313 | 5.919441712 | 1.29E-08 | 4.57E-08 |
| LINC01989 | 0.055681346 | 0.01818837 | -1.614177857 | 2.35E-19 | 4.76E-18 |
| LINC02223 | 0.002746031 | 0.134881801 | 5.61820375 | 7.42E-12 | 4.26E-11 |
| AC120498.9 | 0.017199923 | 0.050199953 | 1.545283902 | 7.14E-07 | 1.97E-06 |
| FAM95B1 | 0.017364346 | 0.004594617 | -1.918111619 | 1.79E-16 | 2.14E-15 |
| AC022733.1 | 0.140869849 | 0.029337771 | -2.26353167 | 1.87E-15 | 1.88E-14 |
| AL161629.1 | 0.100559168 | 0.012862774 | -2.966770882 | 3.94E-21 | 1.24E-19 |
| AC015908.3 | 1.050165535 | 0.153848705 | -2.771032549 | 4.57E-24 | 3.15E-22 |
| AC009275.1 | 0.207982258 | 0.436845401 | 1.07066234 | 0.00282746 | 0.00443943 |
| AC110285.6 | 0.147122597 | 0.99991578 | 2.764787736 | 2.28E-10 | 1.06E-09 |
| B3GALT5-AS1 | 3.897200965 | 0.218697729 | -4.155428186 | 1.93E-19 | 3.99E-18 |
| AC010542.1 | 0.034521454 | 0.136856714 | 1.987101083 | 0.01678004 | 0.02300768 |
| AC092118.1 | 0.001418787 | 0.024693606 | 4.12140736 | 1.46E-06 | 3.84E-06 |
| KCNQ1-AS1 | 0.008416625 | 0.051229191 | 2.605652331 | 0.0032705 | 0.00508093 |
| LINC01942 | 0.026442975 | 0.205718716 | 2.959716671 | 1.75E-09 | 7.03E-09 |
| NCBP2-AS1 | 0.22880811 | 0.576239778 | 1.332531068 | 5.81E-06 | 1.39E-05 |
| AC090116.1 | 0.04859718 | 1.11526258 | 4.520367026 | 6.36E-12 | 3.70E-11 |
| RHOA-IT1 | 0.229632188 | 0.478843056 | 1.060227998 | 0.00809559 | 0.01171583 |
| AP003973.2 | 0.086950626 | 0.026658452 | -1.70560341 | 1.31E-09 | 5.41E-09 |
| SNHG10 | 1.202189035 | 2.586971083 | 1.105600161 | 5.77E-15 | 5.28E-14 |
| AL513327.1 | 0.272124028 | 0.634476814 | 1.221303093 | 0.00010766 | 0.00021057 |
| AC009088.2 | 0.03056207 | 0.154354701 | 2.33643526 | 0.009485 | 0.01356102 |
| AC025580.3 | 0.02322558 | 0.054357883 | 1.226774621 | 0.02918706 | 0.03837884 |
| AL732292.2 | 0.101561731 | 0.284368784 | 1.485406213 | 5.20E-08 | 1.68E-07 |
| AC010655.4 | 0.015005189 | 0.049264642 | 1.715091055 | 0.00015202 | 0.00029086 |
| AL365356.1 | 0.016281853 | 0.304403206 | 4.224646775 | 0.03310348 | 0.04306137 |
| AC025871.2 | 0.019676878 | 0.076487432 | 1.958721409 | 0.01139135 | 0.01608833 |
| TFAP2A-AS1 | 0.04018322 | 0.599963384 | 3.900209382 | 2.26E-15 | 2.23E-14 |
| AC027237.3 | 0.092761821 | 0.211655321 | 1.190113704 | 1.03E-12 | 6.82E-12 |
| AC007881.2 | 0.044465018 | 0.013020471 | -1.771889146 | 2.38E-08 | 8.09E-08 |
| LINC01645 | 2.510503528 | 0.130614517 | -4.264589595 | 3.35E-24 | 2.50E-22 |
| LINC02435 | 0.023375194 | 0.082945593 | 1.827186986 | 0.00348961 | 0.0053906 |
| AC010655.2 | 0.057280876 | 0.277247484 | 2.275048901 | 4.58E-12 | 2.73E-11 |
| AC024060.1 | 1.764880435 | 3.638910701 | 1.043936199 | 4.78E-10 | 2.10E-09 |
| AL512306.3 | 0.29258925 | 0.083992664 | -1.800541526 | 1.45E-17 | 2.08E-16 |
| AL135999.3 | 0.219117454 | 0.603742992 | 1.462230131 | 2.62E-05 | 5.66E-05 |
| AL033381.2 | 0.008521383 | 0.089322221 | 3.389859602 | 2.81E-05 | 6.02E-05 |
| AC008667.1 | 0.023353336 | 0.143525321 | 2.619604743 | 0.00011275 | 0.00021989 |
| AC145207.8 | 0.261047362 | 0.540269492 | 1.049367637 | 0.00323737 | 0.00503089 |
| LINC00689 | 0.029373569 | 0.014505408 | -1.017927705 | 1.97E-13 | 1.43E-12 |
| SP2-AS1 | 0.360194328 | 0.723919011 | 1.00705284 | 6.41E-10 | 2.76E-09 |
| AC092681.2 | 0.018063651 | 0.128875706 | 2.834818939 | 0.00015349 | 0.00029305 |
| AC026771.1 | 0.088650524 | 0.210999075 | 1.251035608 | 0.0274507 | 0.03621792 |
| AC108062.1 | 0.151895977 | 0.427450594 | 1.492674016 | 8.76E-11 | 4.31E-10 |
| AC026469.1 | 0.073777647 | 0.025431344 | -1.536576066 | 1.85E-18 | 3.11E-17 |
| AL358473.1 | 0.013522089 | 0.033038846 | 1.288845278 | 0.03749863 | 0.04835186 |
| AC108681.1 | 0.004116644 | 0.109623331 | 4.73494226 | 1.16E-11 | 6.55E-11 |
| AL138789.1 | 0.058243351 | 0.341316273 | 2.550943944 | 1.26E-05 | 2.86E-05 |
| LINC01258 | 0.006937646 | 0.020945675 | 1.59413421 | 0.0169497 | 0.02322863 |
| AC022217.2 | 0.064263692 | 0.027522939 | -1.223369321 | 2.65E-06 | 6.73E-06 |
| OSTN-AS1 | 0.427943677 | 0.067548749 | -2.663419983 | 1.59E-18 | 2.71E-17 |
| KCNQ1OT1 | 0.022093175 | 0.283769329 | 3.683046013 | 8.13E-16 | 8.69E-15 |
| AC021683.1 | 0.702554884 | 0.164489392 | -2.094616384 | 1.05E-14 | 9.19E-14 |
| AC027601.1 | 0.162787336 | 0.354535374 | 1.122941113 | 3.95E-09 | 1.50E-08 |
| AC099811.5 | 0.026097393 | 0.230144997 | 3.14056546 | 0.03583184 | 0.04636712 |
| AC011468.1 | 0.642827474 | 1.317307205 | 1.035088336 | 4.93E-06 | 1.20E-05 |
| LINC00514 | 0.066783523 | 0.209815652 | 1.651558196 | 4.32E-05 | 9.02E-05 |
| AC027228.2 | 0.409438548 | 0.844461681 | 1.044385021 | 3.85E-09 | 1.47E-08 |
| LINC01389 | 3.061055033 | 1.524652435 | -1.005548584 | 8.23E-12 | 4.70E-11 |
| AC096719.1 | 0.027043947 | 0.06771597 | 1.324190374 | 0.00907958 | 0.01303606 |
| AC239584.1 | 0.006549446 | 0.414776764 | 5.984818335 | 4.66E-12 | 2.78E-11 |
| LINC01121 | 0.007069952 | 0.034355269 | 2.280759005 | 0.00203836 | 0.00327592 |
| AC073323.1 | 0.003401813 | 0.116896123 | 5.102779222 | 3.49E-10 | 1.56E-09 |
| LINC02320 | 0.139761438 | 1.310260663 | 3.228815588 | 7.12E-20 | 1.63E-18 |
| AL365436.2 | 0.10298514 | 0.348219651 | 1.757561438 | 0.0004707 | 0.00084072 |
| AC105137.2 | 0.098736317 | 0.298583109 | 1.596479819 | 2.10E-11 | 1.13E-10 |
| LINC01014 | 0.103145513 | 0.019842567 | -2.378010482 | 1.13E-29 | 2.56E-27 |
| AL590723.1 | 0.206210917 | 0.682281096 | 1.726245531 | 0.00123051 | 0.00204759 |
| AP005329.1 | 0.230733425 | 0.507847293 | 1.138167738 | 6.30E-06 | 1.50E-05 |
| AL357153.1 | 0.004845587 | 0.018353266 | 1.921293349 | 0.02990551 | 0.03921951 |
| AC110813.1 | 0.453031859 | 0.215594344 | -1.071293183 | 1.50E-15 | 1.54E-14 |
| AC078795.1 | 0.067753216 | 0.239766898 | 1.823271169 | 3.00E-06 | 7.56E-06 |
| AC079089.1 | 0.055309355 | 0.252239635 | 2.189199572 | 4.00E-10 | 1.78E-09 |
| AC009084.1 | 0.169857721 | 0.043297287 | -1.971978281 | 3.88E-16 | 4.37E-15 |
| AC135050.4 | 0.049266779 | 0.154252118 | 1.646603239 | 1.30E-05 | 2.95E-05 |
| AL645608.2 | 0.009729133 | 0.078685479 | 3.0157142 | 1.94E-06 | 5.01E-06 |
| AC096637.2 | 0.007270801 | 0.034856678 | 2.261248948 | 0.00043281 | 0.00077686 |
| AL133410.1 | 0.362753262 | 0.929294263 | 1.357146914 | 3.99E-12 | 2.41E-11 |
| AL445426.1 | 0.409053809 | 0.08509017 | -2.265226254 | 2.55E-22 | 1.10E-20 |
| AC027243.1 | 0.179643281 | 0.062498384 | -1.52324418 | 5.84E-11 | 2.95E-10 |
| AC092667.1 | 0.039362033 | 0.006741423 | -2.545679571 | 3.29E-23 | 1.77E-21 |
| FLJ31104 | 0.186273929 | 0.073334897 | -1.344853978 | 2.07E-15 | 2.05E-14 |
| MACC1-AS1 | 0.014264832 | 0.608254034 | 5.414139279 | 0.00203815 | 0.00327592 |
| AL035458.2 | 0.146187005 | 0.742298685 | 2.344184744 | 2.65E-15 | 2.59E-14 |
| LINC02474 | 0.001570836 | 0.752076616 | 8.903203353 | 7.02E-11 | 3.50E-10 |
| AL356124.1 | 0.026560067 | 0.222213682 | 3.064616969 | 0.00218335 | 0.00348939 |
| LINC00865 | 0.597914544 | 0.283837426 | -1.074874472 | 4.37E-11 | 2.25E-10 |
| AC090709.1 | 0.035429442 | 0.628665554 | 4.149272064 | 1.52E-11 | 8.38E-11 |
| AC016924.1 | 0.099217508 | 0.034867608 | -1.508707332 | 7.39E-12 | 4.25E-11 |
| AC068620.2 | 0.165689271 | 0.342780124 | 1.048803269 | 0.00224522 | 0.00358198 |
| AC104051.2 | 0.042819376 | 0.007327768 | -2.546818029 | 2.90E-26 | 4.16E-24 |
| AL359880.1 | 0.037809808 | 0.499905517 | 3.724823027 | 0.00111671 | 0.00187079 |
| AC027807.2 | 0.214078429 | 0.049723688 | -2.106134232 | 7.45E-17 | 9.53E-16 |
| AC127024.6 | 0.194198256 | 0.441853349 | 1.186037376 | 9.51E-05 | 0.00018782 |
| MIR497HG | 0.409022988 | 0.088949585 | -2.201122142 | 1.13E-21 | 4.02E-20 |
| AC107959.2 | 0.003583604 | 0.035579557 | 3.311565425 | 8.93E-05 | 0.00017696 |
| ISPD-AS1 | 0.007935777 | 0.038193795 | 2.266894807 | 0.01520561 | 0.02102337 |
| AC121757.1 | 0.069607906 | 0.19398259 | 1.478604092 | 2.68E-05 | 5.77E-05 |
| MANCR | 0.660514282 | 0.30627943 | -1.108741279 | 4.35E-12 | 2.61E-11 |
| AC025034.1 | 0.181047274 | 0.404609767 | 1.160164689 | 0.02091243 | 0.02816404 |
| Z92544.1 | 0.009604737 | 0.050886912 | 2.405476677 | 3.67E-07 | 1.06E-06 |
| AL645608.7 | 0.185761868 | 0.459716973 | 1.307291548 | 3.43E-06 | 8.53E-06 |
| AC015813.1 | 0.944762331 | 2.887714762 | 1.611904896 | 1.54E-10 | 7.32E-10 |
| MHENCR | 2.25888012 | 8.461957944 | 1.905383807 | 4.84E-19 | 9.12E-18 |
| AC004870.2 | 0.156806605 | 0.074321799 | -1.077128991 | 7.45E-16 | 8.01E-15 |
| AL391095.1 | 0.076604528 | 0.332782027 | 2.119075941 | 4.71E-10 | 2.08E-09 |
| DDC-AS1 | 0.007921087 | 0.06757565 | 3.092733228 | 0.00011683 | 0.00022695 |
| AL136537.2 | 0.110566348 | 0.019314194 | -2.517178965 | 1.98E-19 | 4.08E-18 |
| AL158211.1 | 0.128740086 | 0.046617829 | -1.465507598 | 9.16E-15 | 8.15E-14 |
| AC005920.4 | 0.035291687 | 0.100335645 | 1.507433921 | 0.00350794 | 0.00541739 |
| ADAMTS9-AS1 | 0.606049155 | 0.112361528 | -2.431286663 | 6.79E-16 | 7.34E-15 |
| AL022157.1 | 0.165409948 | 0.41379911 | 1.322884541 | 0.00036089 | 0.00065598 |
| AL590133.1 | 0.087478756 | 0.187420567 | 1.099274663 | 0.00252358 | 0.00399798 |
| AC121761.1 | 0.268384403 | 1.003255556 | 1.902316407 | 4.20E-14 | 3.35E-13 |
| AC002116.2 | 0.426804926 | 1.015592886 | 1.250673462 | 2.15E-14 | 1.79E-13 |
| AC091057.1 | 0.485327232 | 1.034769916 | 1.092280298 | 1.55E-11 | 8.53E-11 |
| LINC01585 | 0.016833665 | 0.044846894 | 1.413658707 | 0.01432101 | 0.01991647 |
| AC135279.3 | 0.020251429 | 0.098350933 | 2.279914985 | 3.24E-09 | 1.26E-08 |
| AC135178.5 | 0.276919938 | 0.672440454 | 1.279937591 | 5.07E-13 | 3.54E-12 |
| AC018695.3 | 0.014279763 | 0.117979786 | 3.046495734 | 1.97E-05 | 4.35E-05 |
| C10orf91 | 0.201615107 | 0.464427665 | 1.20385017 | 1.41E-09 | 5.75E-09 |
| LINC00958 | 0.022392778 | 0.151628509 | 2.759435581 | 8.13E-07 | 2.22E-06 |
| AL360093.1 | 0.016089767 | 0.128987085 | 3.003011288 | 0.00479312 | 0.00723612 |
| LINC02289 | 0.068346904 | 0.024267362 | -1.493858683 | 4.98E-13 | 3.48E-12 |
| AC137932.2 | 0.02842364 | 0.206469892 | 2.860768215 | 1.48E-09 | 6.00E-09 |
| AL669942.1 | 0.002388377 | 0.000586498 | -2.025831675 | 4.24E-11 | 2.18E-10 |
| LINC02245 | 0.035000151 | 0.011478118 | -1.608475083 | 9.94E-12 | 5.64E-11 |
| LINC02588 | 0.516900635 | 0.211353714 | -1.290227514 | 5.52E-13 | 3.83E-12 |
| AC114811.2 | 0.027348477 | 0.118168221 | 2.111309701 | 4.42E-10 | 1.95E-09 |
| AL033543.1 | 0.002486889 | 0.049269735 | 4.308287545 | 1.20E-08 | 4.26E-08 |
| AL022322.2 | 0.006644806 | 0.019947785 | 1.58592953 | 0.00192798 | 0.00311407 |
| MIR31HG | 0.034024622 | 0.312879792 | 3.20095745 | 3.03E-10 | 1.38E-09 |
| LINC02081 | 0.038844251 | 0.432696364 | 3.477582001 | 6.11E-18 | 9.36E-17 |
| AC104794.3 | 0.001680615 | 0.12370254 | 6.20174242 | 3.54E-12 | 2.15E-11 |
| AC136475.5 | 0.059339661 | 0.260154884 | 2.132302198 | 1.09E-07 | 3.39E-07 |
| ZNF433-AS1 | 0.369007875 | 0.789245934 | 1.096823317 | 7.95E-15 | 7.17E-14 |
| AC112206.2 | 0.013212125 | 0.039320845 | 1.573431772 | 0.00333924 | 0.00517445 |
| FARP1-AS1 | 0.078254701 | 0.290204279 | 1.890819473 | 4.99E-05 | 0.00010305 |
| AC005776.2 | 0.082073546 | 0.192012559 | 1.226211478 | 3.85E-07 | 1.10E-06 |
| AC116345.1 | 0.059235055 | 0.777782965 | 3.71484453 | 7.47E-06 | 1.76E-05 |
| STAM-AS1 | 0.069725642 | 0.185553684 | 1.412075429 | 1.02E-09 | 4.26E-09 |
| AC018695.4 | 0.222748889 | 0.551842836 | 1.30883921 | 6.34E-07 | 1.77E-06 |
| AP002812.2 | 0.058623673 | 0.151072787 | 1.365688543 | 0.00742437 | 0.01082199 |
| RBMS3-AS3 | 0.311406062 | 0.075203018 | -2.049934572 | 9.80E-22 | 3.56E-20 |
| AJ011932.1 | 0.217365594 | 0.084949995 | -1.355437836 | 1.61E-09 | 6.50E-09 |
| AC137770.1 | 0.065610299 | 0.174230598 | 1.409003812 | 0.03516147 | 0.04556452 |
| TMEM147-AS1 | 0.626373706 | 2.220382677 | 1.825712786 | 1.45E-20 | 3.94E-19 |
| AC244517.7 | 0.110232019 | 0.516707704 | 2.228805049 | 0.00653564 | 0.00963692 |
| CRYZL2P-SEC16B | 0.094048888 | 0.247422834 | 1.395495862 | 3.10E-06 | 7.78E-06 |
| AC004943.3 | 0.040876107 | 0.117255607 | 1.520327222 | 0.02079822 | 0.02803775 |
| AC017002.1 | 0.01417467 | 0.303898935 | 4.422204576 | 0.00078792 | 0.00135751 |
| AC100821.2 | 0.055030969 | 0.214898696 | 1.965341076 | 0.00310524 | 0.00484351 |
| AC036108.3 | 1.221945565 | 0.188243278 | -2.698509765 | 1.94E-16 | 2.30E-15 |
| AC009404.1 | 0.177873479 | 0.567851233 | 1.674661602 | 3.29E-18 | 5.22E-17 |
| MALAT1 | 7.994962528 | 131.4480176 | 4.039257302 | 2.45E-08 | 8.30E-08 |
| AC245884.1 | 0.011478885 | 0.085799262 | 2.901982703 | 2.27E-05 | 4.99E-05 |
| AC020978.4 | 3.249120812 | 0.518077514 | -2.648809517 | 2.76E-23 | 1.52E-21 |
| AL080317.2 | 0.149475166 | 0.571773701 | 1.935538454 | 4.80E-10 | 2.11E-09 |
| LINC00844 | 0.100544829 | 0.015065574 | -2.738511371 | 3.98E-28 | 7.74E-26 |
| AC132192.1 | 0.05684945 | 0.378251217 | 2.73412643 | 8.60E-07 | 2.33E-06 |
| LINC01775 | 0.114988668 | 0.251955559 | 1.131677599 | 0.00034315 | 0.00062561 |
| DDX11-AS1 | 0.117390178 | 0.387110566 | 1.721433979 | 1.90E-19 | 3.95E-18 |
| AL606763.1 | 0.112502693 | 0.366521648 | 1.703938868 | 1.14E-06 | 3.06E-06 |
| WASHC5-AS1 | 0.094633266 | 0.38220527 | 2.013928343 | 1.72E-06 | 4.47E-06 |
| AC064836.2 | 0.023220821 | 0.117022652 | 2.333296914 | 4.77E-05 | 9.89E-05 |
| ALMS1-IT1 | 0.100580879 | 0.412883431 | 2.037378456 | 3.85E-13 | 2.71E-12 |
| AC009299.3 | 0.035531537 | 0.123433057 | 1.796556793 | 0.00073422 | 0.00127061 |
| AC000061.1 | 0.169340034 | 0.958237615 | 2.500460368 | 9.67E-05 | 0.00019067 |
| AL355385.1 | 0.177719659 | 0.474801819 | 1.417722184 | 8.50E-08 | 2.67E-07 |
| LINC01833 | 0.016378062 | 0.380731762 | 4.538938365 | 6.62E-08 | 2.11E-07 |
| AC026470.2 | 0.070265864 | 0.529400771 | 2.91346441 | 0.00554006 | 0.00827669 |
| AC021739.3 | 0.038584797 | 0.133458209 | 1.790283627 | 0.00046008 | 0.00082282 |
| AL158151.4 | 0.068222449 | 0.221361907 | 1.69808852 | 0.00014227 | 0.00027307 |
| KCNMB2-AS1 | 0.02995218 | 0.579925845 | 4.275135513 | 9.19E-09 | 3.32E-08 |
| AL512363.1 | 1.222350261 | 0.350314179 | -1.802936457 | 2.99E-16 | 3.42E-15 |
| SLCO4A1-AS1 | 0.558654385 | 7.537742377 | 3.754104556 | 2.63E-18 | 4.29E-17 |
| AC004908.2 | 0.401542808 | 0.881544898 | 1.134480249 | 5.22E-07 | 1.47E-06 |
| AC092745.1 | 0.011718576 | 0.061182381 | 2.384318942 | 9.63E-05 | 0.00019006 |
| AC007314.1 | 0.035100936 | 0.185549102 | 2.402219612 | 0.03812091 | 0.04911941 |
| AL117332.1 | 0.372344803 | 0.85294311 | 1.195810296 | 1.20E-11 | 6.72E-11 |
| AL583810.1 | 0.17652445 | 0.379986384 | 1.106079698 | 0.00017152 | 0.0003252 |
| AL355802.2 | 0.17001207 | 0.578479591 | 1.76662889 | 9.91E-13 | 6.61E-12 |
| AC004707.1 | 0.7837532 | 0.135455913 | -2.532576054 | 1.25E-22 | 5.62E-21 |
| AC083967.1 | 0.004198411 | 0.155380464 | 5.209817923 | 8.61E-08 | 2.70E-07 |
| AC079061.1 | 0.014104499 | 0.047846559 | 1.76225978 | 0.00513535 | 0.00771009 |
| AC004264.1 | 0.225997831 | 0.787966822 | 1.801825959 | 2.45E-15 | 2.40E-14 |
| AC145423.3 | 0.121354116 | 0.410832992 | 1.759329006 | 1.82E-09 | 7.29E-09 |
| LINC01484 | 0.016932656 | 0.090813245 | 2.423094424 | 6.18E-12 | 3.60E-11 |
| UBAC2-AS1 | 0.37625325 | 0.818731173 | 1.121685784 | 1.88E-11 | 1.02E-10 |
| AL591222.1 | 0.000750578 | 0.024309243 | 5.01735881 | 6.02E-07 | 1.68E-06 |
| TDRKH-AS1 | 0.314557618 | 0.667163996 | 1.084717134 | 1.91E-08 | 6.58E-08 |
| IBA57-DT | 0.132367718 | 0.320376603 | 1.275217478 | 1.68E-05 | 3.76E-05 |
| LINC01270 | 0.050874803 | 0.182359349 | 1.841760949 | 4.18E-14 | 3.34E-13 |
| AP001893.1 | 0.190437356 | 0.44842258 | 1.235542419 | 0.00177172 | 0.00288049 |
| AC015853.1 | 0.019128805 | 0.147121956 | 2.94319393 | 7.08E-06 | 1.68E-05 |
| AC084262.1 | 0.014844048 | 0.103098724 | 2.796069971 | 0.00050207 | 0.00089092 |
| AL121899.1 | 0.148316604 | 0.938443492 | 2.661589759 | 1.25E-13 | 9.30E-13 |
| AC009163.5 | 1.336239771 | 0.377221881 | -1.824693634 | 7.41E-13 | 5.03E-12 |
| NARF-AS1 | 0.037109084 | 0.106465014 | 1.520535101 | 0.00035671 | 0.00064902 |
| AC015922.2 | 8.15835586 | 2.566953494 | -1.668221279 | 1.66E-21 | 5.69E-20 |
| CTD-2270F17.1 | 0.084535793 | 0.014975931 | -2.49691666 | 9.11E-16 | 9.68E-15 |
| AC036108.2 | 0.075076366 | 0.025522126 | -1.556610342 | 1.24E-12 | 8.04E-12 |
| SNHG3 | 3.519218931 | 10.97030425 | 1.640276365 | 7.68E-20 | 1.73E-18 |
| AC005291.2 | 0.019097914 | 0.09751475 | 2.352205427 | 0.00071872 | 0.00124617 |
| MRGPRF-AS1 | 0.126779876 | 0.02758667 | -2.200282539 | 8.41E-14 | 6.48E-13 |
| AL138902.1 | 0.013619773 | 0.064788806 | 2.250041909 | 0.00019376 | 0.00036509 |
| AC011446.1 | 0.044996095 | 0.136753959 | 1.603710893 | 0.00035668 | 0.00064902 |
| AC098613.1 | 1.010058408 | 2.236642738 | 1.14689611 | 2.28E-07 | 6.74E-07 |
| LINC02397 | 0.049480429 | 0.016672188 | -1.569414575 | 1.10E-14 | 9.58E-14 |
| AP003419.3 | 0.242223945 | 0.557950215 | 1.203794908 | 2.76E-07 | 8.07E-07 |
| AL365277.1 | 0.167462896 | 0.585203386 | 1.805096635 | 0.00180046 | 0.00292199 |
| AC126177.8 | 0.018198787 | 0.009096878 | -1.000398939 | 1.27E-07 | 3.90E-07 |
| AC010536.2 | 0.256512043 | 0.861016999 | 1.747015161 | 0.01262209 | 0.01771185 |
| AC105383.1 | 0.011355525 | 0.004978294 | -1.189670923 | 1.78E-12 | 1.13E-11 |
| AC244021.1 | 0.091420557 | 0.030027343 | -1.606241792 | 2.91E-12 | 1.80E-11 |
| AC009630.2 | 0.002928367 | 0.026578567 | 3.18209492 | 1.28E-06 | 3.40E-06 |
| MGC12916 | 0.119947444 | 0.268677862 | 1.163475041 | 0.00709819 | 0.01037428 |
| AC093732.1 | 0.029839412 | 0.583537374 | 4.289534027 | 1.54E-13 | 1.13E-12 |
| AC004241.3 | 0.43579132 | 0.963319241 | 1.144376521 | 4.47E-08 | 1.46E-07 |
| AC008870.2 | 0.281877969 | 0.673659958 | 1.256949826 | 3.72E-09 | 1.43E-08 |
| SLC2A1-AS1 | 0.077205075 | 0.22122875 | 1.518771293 | 4.73E-07 | 1.34E-06 |
| AP001025.1 | 0.033727187 | 0.116467909 | 1.787948591 | 0.01526169 | 0.02109022 |
| AC013652.1 | 0.091722513 | 0.296901858 | 1.694638327 | 1.43E-05 | 3.24E-05 |
| AC110491.1 | 0.193693518 | 0.013289299 | -3.865438758 | 1.77E-23 | 1.06E-21 |
| SNHG17 | 2.753759518 | 12.94545672 | 2.232971383 | 6.43E-23 | 3.13E-21 |
| AP003064.2 | 0.079484693 | 0.025873853 | -1.619182175 | 1.62E-12 | 1.03E-11 |
| LINC01857 | 0.870698397 | 0.414779026 | -1.069830124 | 1.16E-05 | 2.65E-05 |
| AP000593.3 | 0.042410601 | 0.177883884 | 2.068438958 | 1.03E-06 | 2.77E-06 |
| AC027319.1 | 0.160348343 | 0.345641363 | 1.10806643 | 0.00190442 | 0.00307971 |
| AC018647.1 | 0.080220343 | 0.031955427 | -1.327907186 | 4.99E-10 | 2.19E-09 |
| AC005277.2 | 0.327762279 | 0.142435244 | -1.20234366 | 2.12E-08 | 7.25E-08 |
| AC007998.4 | 0.012032948 | 0.047934492 | 1.99407396 | 1.08E-06 | 2.89E-06 |
| LINC01952 | 0.036300365 | 0.129493365 | 1.834822213 | 8.37E-05 | 0.00016664 |
| GK-IT1 | 0.062842341 | 0.57121952 | 3.184236445 | 1.14E-05 | 2.61E-05 |
| LINC01186 | 0.117716523 | 0.572946681 | 2.283084053 | 6.20E-16 | 6.73E-15 |
| AP001178.3 | 0.017234931 | 0.069682222 | 2.015455078 | 1.48E-06 | 3.88E-06 |
| AL049795.1 | 0.072499556 | 0.192254378 | 1.406972385 | 3.90E-09 | 1.48E-08 |
| AC120498.8 | 0.045103396 | 0.111638977 | 1.307532846 | 0.00013114 | 0.00025322 |
| AC105105.1 | 0.042858132 | 0.007792448 | -2.459420365 | 5.86E-23 | 2.93E-21 |
| SNHG20 | 0.966465647 | 2.333083853 | 1.271447801 | 2.15E-18 | 3.55E-17 |
| AL592494.3 | 0.136098869 | 0.052083537 | -1.385755731 | 1.38E-13 | 1.02E-12 |
| AC135586.2 | 0.285666055 | 0.058952625 | -2.276701661 | 3.75E-20 | 9.26E-19 |
| LINC01545 | 0.048476117 | 0.153687116 | 1.664650173 | 1.85E-05 | 4.12E-05 |
| AL158837.1 | 1.16455664 | 0.502141042 | -1.213616258 | 9.28E-16 | 9.82E-15 |
| SAP30L-AS1 | 0.187374633 | 0.389229959 | 1.054697108 | 0.00719404 | 0.0105031 |
| AF165147.1 | 0.116660792 | 0.033802486 | -1.787118521 | 1.76E-19 | 3.68E-18 |
| LNCARSR | 0.007413948 | 0.002997743 | -1.306365242 | 1.70E-16 | 2.04E-15 |
| AL355974.2 | 0.058385011 | 0.015057169 | -1.955147447 | 1.05E-15 | 1.11E-14 |
| LINC02541 | 0.701609981 | 0.224345215 | -1.64494886 | 7.57E-09 | 2.79E-08 |
| AC093673.2 | 0.081905442 | 0.421504884 | 2.363518136 | 0.00251117 | 0.00397949 |
| LINC01594 | 0.035219261 | 0.801024244 | 4.507409374 | 3.30E-16 | 3.76E-15 |
| AL360004.1 | 0.051810419 | 0.017612912 | -1.556608755 | 0.00011455 | 0.00022293 |
| AC012213.3 | 0.045838349 | 0.280405947 | 2.612889974 | 5.32E-11 | 2.71E-10 |
| LINC00908 | 0.100075923 | 0.02217931 | -2.173808558 | 9.38E-22 | 3.43E-20 |
| AC112512.1 | 0.01095458 | 0.168419301 | 3.942451398 | 0.01154828 | 0.01628887 |
| AC067930.3 | 0.068104459 | 0.148818333 | 1.127731112 | 0.00417001 | 0.0063588 |
| AL356515.1 | 0.064540524 | 0.019161702 | -1.751979538 | 3.38E-09 | 1.31E-08 |
| AL031665.1 | 0.027743408 | 0.184462371 | 2.733109607 | 0.00061579 | 0.00107937 |
| AC006460.1 | 0.021016464 | 0.205919704 | 3.292490049 | 0.02361547 | 0.03155481 |
| LINC02043 | 0.008622747 | 0.055121952 | 2.676407533 | 3.01E-08 | 1.01E-07 |
| DNAH17-AS1 | 0.002719666 | 0.043792878 | 4.009195105 | 7.15E-13 | 4.87E-12 |
| AL391095.3 | 0.076692552 | 0.268287158 | 1.806619617 | 1.91E-07 | 5.72E-07 |
| AGAP2-AS1 | 2.281977581 | 5.309126737 | 1.218189963 | 1.25E-11 | 7.00E-11 |
| LINC01571 | 0.157006794 | 0.028816725 | -2.445848681 | 3.19E-28 | 6.43E-26 |
| LINC01485 | 0.026994005 | 0.314114136 | 3.540577911 | 6.86E-09 | 2.54E-08 |
| AC087379.2 | 0.484621304 | 0.044150408 | -3.456359157 | 1.96E-24 | 1.60E-22 |
| LINC01705 | 0.005419936 | 1.312204036 | 7.919500448 | 1.57E-20 | 4.23E-19 |
| LINC00323 | 0.042733362 | 0.107565999 | 1.331787397 | 0.00081461 | 0.00139907 |
| AC026904.2 | 0.063878523 | 0.024738949 | -1.368546735 | 9.55E-10 | 4.01E-09 |
| FTX | 0.19336806 | 1.238558217 | 2.679240263 | 3.08E-06 | 7.74E-06 |
| AC008514.1 | 0.025669966 | 0.254961674 | 3.312127115 | 0.00188907 | 0.00305671 |
| AC092683.1 | 0.021236635 | 0.050387712 | 1.24651675 | 0.0016859 | 0.00275494 |
| AL133467.1 | 0.400592506 | 0.146947056 | -1.446838973 | 1.77E-12 | 1.12E-11 |
| HCG15 | 0.18899161 | 0.420110629 | 1.152447094 | 6.72E-10 | 2.89E-09 |
| AC010422.2 | 0.073586277 | 0.230024291 | 1.644277576 | 5.04E-09 | 1.90E-08 |
| AP001160.1 | 0.203776837 | 0.755283076 | 1.890027386 | 9.62E-18 | 1.43E-16 |
| AP001029.2 | 0.024505594 | 0.08355262 | 1.769573978 | 1.41E-07 | 4.30E-07 |
| LINC02292 | 0.248004351 | 0.060726462 | -2.0299682 | 3.84E-18 | 6.07E-17 |
| AC004223.2 | 0.023611227 | 0.360507021 | 3.932482424 | 0.00052256 | 0.00092396 |
| AC010530.1 | 0.106973424 | 0.225847357 | 1.078095605 | 0.01768707 | 0.02414208 |
| LINC01788 | 0.013616967 | 0.006001204 | -1.182081635 | 2.03E-11 | 1.10E-10 |
| AC068831.6 | 0.038929129 | 0.098366598 | 1.337318445 | 0.00213869 | 0.00342404 |
| LINC00702 | 0.363789736 | 0.145832634 | -1.31879124 | 8.90E-15 | 7.95E-14 |
| AL512770.1 | 0.233358655 | 0.512957091 | 1.136289176 | 2.01E-10 | 9.35E-10 |
| AC009171.2 | 0.202566306 | 0.497444234 | 1.29614058 | 1.18E-06 | 3.15E-06 |
| LINC02407 | 0.016258529 | 0.079216216 | 2.284599062 | 6.69E-10 | 2.87E-09 |
| AL031716.1 | 0.318503885 | 0.9638441 | 1.597488843 | 3.25E-10 | 1.46E-09 |
| AL512652.1 | 0.069632939 | 0.379864361 | 2.447642539 | 8.99E-11 | 4.42E-10 |
| AL590369.1 | 0.083447569 | 0.171782889 | 1.041644404 | 0.00458591 | 0.00695024 |
| LINC01342 | 0.203407273 | 0.486516721 | 1.258118128 | 1.68E-06 | 4.37E-06 |
| AC084117.1 | 0.237641229 | 0.735883393 | 1.630692021 | 4.55E-05 | 9.49E-05 |
| AC008543.1 | 0.082871592 | 0.171103838 | 1.045922577 | 3.02E-06 | 7.59E-06 |
| AL049830.3 | 0.014381168 | 0.057479377 | 1.998863548 | 0.00778124 | 0.01131497 |
| ADAMTS9-AS2 | 0.192225281 | 0.048043838 | -2.000374774 | 7.25E-20 | 1.65E-18 |
| AC064805.1 | 0.028337027 | 0.113239914 | 1.998622248 | 0.00619454 | 0.00916906 |
| AC138430.1 | 0.013173361 | 0.004044806 | -1.703481257 | 0.00144965 | 0.0023884 |
| AC024581.1 | 0.007865912 | 0.071654238 | 3.187366129 | 1.34E-05 | 3.04E-05 |
| AL161630.1 | 0.077776017 | 0.381422463 | 2.293992546 | 0.00504233 | 0.0075746 |
| AL031673.1 | 0.40346328 | 1.256573525 | 1.638985806 | 2.81E-09 | 1.10E-08 |
| AC087222.1 | 0.12454095 | 0.580315872 | 2.220218196 | 3.70E-07 | 1.06E-06 |
| TTC3-AS1 | 0.039821708 | 0.157141302 | 1.980435425 | 0.00123375 | 0.00205237 |
| AC108727.1 | 0.100502871 | 0.610136916 | 2.601896307 | 0.01856167 | 0.02527261 |
| AC016575.1 | 0.072456663 | 0.180146735 | 1.313982238 | 0.00172219 | 0.00280835 |
| MIR4713HG | 0.118676797 | 0.519680926 | 2.130588216 | 5.26E-13 | 3.66E-12 |
| AC084200.1 | 0.211898552 | 0.027078336 | -2.96816271 | 4.80E-23 | 2.47E-21 |
| LINC02334 | 0.011830911 | 0.061573522 | 2.379748888 | 0.00100445 | 0.00169733 |
| AC023158.1 | 0.027594943 | 0.009969119 | -1.468865914 | 4.26E-20 | 1.04E-18 |
| AC026369.2 | 0.066979001 | 0.455839448 | 2.766745019 | 6.55E-21 | 1.96E-19 |
| AC099522.2 | 0.213931728 | 0.488572427 | 1.191421981 | 3.32E-10 | 1.49E-09 |
| AC019254.1 | 0.055412903 | 0.118419121 | 1.095608188 | 0.00162746 | 0.00266583 |
| AC105384.1 | 0.088850879 | 0.039326825 | -1.175872322 | 3.25E-13 | 2.31E-12 |
| AL161452.1 | 0.116553116 | 0.320201583 | 1.45799286 | 2.37E-11 | 1.27E-10 |
| AC027682.4 | 0.062718948 | 0.168369056 | 1.424653742 | 6.31E-05 | 0.00012834 |
| AC063948.1 | 0.286343694 | 0.628703241 | 1.134631368 | 5.96E-06 | 1.43E-05 |
| AC022915.2 | 0.040780776 | 0.012893081 | -1.661292201 | 3.76E-15 | 3.56E-14 |
| AL109955.1 | 0.286020841 | 0.123927951 | -1.206618659 | 3.70E-09 | 1.42E-08 |
| PTPRG-AS1 | 0.025663801 | 0.111819328 | 2.123362837 | 0.00305988 | 0.00477685 |
| Z97200.1 | 0.005938518 | 0.162809772 | 4.776940643 | 4.60E-09 | 1.74E-08 |
| ZNF667-AS1 | 1.299784974 | 0.414096801 | -1.650233011 | 6.32E-17 | 8.20E-16 |
| AL157395.1 | 0.035200211 | 0.18842525 | 2.420336313 | 0.0061627 | 0.00912689 |
| ANKRD10-IT1 | 4.238488978 | 11.51423393 | 1.441796488 | 4.93E-06 | 1.20E-05 |
| AC012676.3 | 0.221536516 | 0.460049282 | 1.054243894 | 0.0015728 | 0.00258249 |
| AC027279.1 | 0.022378698 | 0.147612476 | 2.721616648 | 0.01538456 | 0.02123849 |
| AL033504.1 | 0.028136253 | 0.226791585 | 3.010864987 | 0.0046294 | 0.00701031 |
| SNHG1 | 4.042356098 | 15.38706754 | 1.928449988 | 1.86E-23 | 1.10E-21 |
| C9orf163 | 0.074318162 | 0.286800382 | 1.948260216 | 5.92E-17 | 7.71E-16 |
| LINC02544 | 0.019671283 | 0.33949202 | 4.109214675 | 6.23E-08 | 1.99E-07 |
| AL355987.4 | 0.351978097 | 1.292089214 | 1.876148126 | 1.03E-11 | 5.82E-11 |
| AC010976.1 | 0.11200013 | 0.331055984 | 1.563574804 | 1.21E-06 | 3.21E-06 |
| RSF1-IT1 | 0.029110274 | 0.149490252 | 2.360451092 | 0.03570592 | 0.04622611 |
| AC103563.7 | 0.062781133 | 0.019109191 | -1.716064377 | 8.61E-14 | 6.60E-13 |
| AL135902.1 | 0.037484489 | 0.125059818 | 1.738252663 | 0.00105525 | 0.00177766 |
| AC104825.1 | 0.981272861 | 0.441865141 | -1.151048241 | 4.21E-16 | 4.72E-15 |
| N4BP2L2-IT2 | 0.258799524 | 0.91708898 | 1.825226754 | 5.21E-05 | 0.0001074 |
| LINC01799 | 0.067265351 | 0.014463157 | -2.217481074 | 9.66E-16 | 1.02E-14 |
| AC009159.3 | 0.338797104 | 0.125544104 | -1.432227273 | 6.97E-14 | 5.45E-13 |
| ZFY-AS1 | 0.197497415 | 0.082518966 | -1.259036129 | 0.00603498 | 0.00895719 |
| AC097717.1 | 0.00953985 | 0.029825017 | 1.644484539 | 0.00315993 | 0.00492317 |
| AL731563.3 | 0.095101207 | 0.219942039 | 1.20958782 | 1.72E-08 | 5.96E-08 |
| AL121906.2 | 0.177626805 | 0.462595083 | 1.38090062 | 1.04E-08 | 3.74E-08 |
| AC084855.1 | 0.03967314 | 0.017734665 | -1.1615905 | 5.43E-07 | 1.52E-06 |
| AL360091.1 | 0.076026572 | 0.242421788 | 1.672943728 | 0.0187704 | 0.02552493 |
| LINC01532 | 0.01227622 | 0.006077115 | -1.014407857 | 3.89E-15 | 3.68E-14 |
| AL121821.1 | 0.090339926 | 0.043168089 | -1.065398495 | 3.00E-09 | 1.17E-08 |
| TTLL10-AS1 | 0.014672425 | 0.045574162 | 1.635108819 | 0.03100086 | 0.04053901 |
| LINC00892 | 0.297946318 | 0.128372359 | -1.214717824 | 4.98E-12 | 2.96E-11 |
| LINC00592 | 0.001831114 | 0.042872516 | 4.549259203 | 6.58E-09 | 2.44E-08 |
| LINC00940 | 0.689360277 | 0.126728515 | -2.443516994 | 1.93E-19 | 3.99E-18 |
| TERC | 0.019706126 | 0.260629952 | 3.725286788 | 0.00118735 | 0.00198062 |
| AC019080.5 | 0.369386415 | 0.870964149 | 1.237482524 | 1.58E-07 | 4.78E-07 |
| AC002563.1 | 0.03315759 | 0.143764129 | 2.116292688 | 0.00100501 | 0.00169775 |
| AC008649.2 | 0.051843225 | 1.102149513 | 4.410020679 | 0.00101419 | 0.00171219 |
| AC022784.6 | 0.023313522 | 0.200487075 | 3.104270357 | 6.22E-09 | 2.32E-08 |
| AC073592.1 | 0.03063445 | 0.190209301 | 2.634360928 | 4.83E-05 | 0.00010014 |
| LINC00862 | 0.008179991 | 0.069279016 | 3.082247335 | 9.34E-08 | 2.92E-07 |
| AC007823.1 | 0.074630789 | 0.177660151 | 1.251277274 | 1.47E-06 | 3.86E-06 |
| AC129102.1 | 0.071887238 | 0.024522719 | -1.551616717 | 2.15E-20 | 5.63E-19 |
| AC073335.2 | 0.817551365 | 2.316278872 | 1.502427679 | 6.66E-13 | 4.55E-12 |
| AC108463.2 | 0.075571293 | 0.316015807 | 2.064086522 | 1.76E-08 | 6.10E-08 |
| AL021578.1 | 0.141787581 | 0.881351551 | 2.635986417 | 3.68E-12 | 2.23E-11 |
| AC244035.1 | 0.040854005 | 0.006313891 | -2.693876218 | 5.57E-21 | 1.70E-19 |
| AP002812.3 | 0.05519002 | 0.19934628 | 1.852797375 | 0.00817961 | 0.011828 |
| EPN2-AS1 | 0.018286035 | 0.157509683 | 3.106626304 | 0.00657184 | 0.00968285 |
| AC091182.1 | 0.218193014 | 0.102017977 | -1.096781517 | 1.14E-11 | 6.43E-11 |
| AP002852.1 | 0.060769158 | 0.155887225 | 1.359091485 | 0.0080001 | 0.01158686 |
| AL161756.1 | 0.034473894 | 0.094101733 | 1.448717025 | 2.38E-10 | 1.10E-09 |
| AP001542.3 | 4.456813868 | 1.942612386 | -1.198014645 | 3.45E-19 | 6.75E-18 |
| AP001107.4 | 0.229828624 | 0.468111353 | 1.026293264 | 0.00304452 | 0.00475968 |
| AC114763.1 | 0.128797157 | 0.33356493 | 1.372866869 | 1.05E-08 | 3.75E-08 |
| LINC01976 | 0.08030919 | 0.019677595 | -2.029011204 | 7.78E-24 | 4.99E-22 |
| AC092112.1 | 0.03402533 | 0.636646886 | 4.225812371 | 7.68E-05 | 0.00015377 |
| AC100774.1 | 0.008934646 | 0.14882938 | 4.058104955 | 0.01911626 | 0.02592414 |
| AC005519.1 | 0.235718152 | 0.528554114 | 1.164988324 | 1.35E-06 | 3.58E-06 |
| AC118754.1 | 0.041602709 | 0.359347791 | 3.110631443 | 1.32E-09 | 5.41E-09 |
| AP001257.1 | 0.06434186 | 0.025961473 | -1.309385402 | 1.81E-06 | 4.69E-06 |
| LINC02345 | 0.013441354 | 0.201384392 | 3.905201497 | 1.71E-14 | 1.46E-13 |
| AFAP1-AS1 | 0.031715885 | 2.150645287 | 6.083420175 | 1.11E-06 | 2.99E-06 |
| ID2-AS1 | 1.016899973 | 0.43243766 | -1.2336137 | 4.53E-14 | 3.60E-13 |
| ATP2A1-AS1 | 0.380253363 | 1.407054008 | 1.887644793 | 8.98E-15 | 8.00E-14 |
| TMEM220-AS1 | 1.5010779 | 0.259908434 | -2.529923493 | 4.14E-25 | 3.96E-23 |
| AC004854.2 | 0.128947771 | 0.290792859 | 1.173205006 | 4.84E-05 | 0.00010024 |
| DLEU1 | 0.532370821 | 1.099434982 | 1.046258884 | 1.46E-08 | 5.14E-08 |
| AC025031.4 | 0.044590334 | 0.236969641 | 2.40989934 | 8.03E-08 | 2.53E-07 |
| AC078820.1 | 0.009678639 | 0.192056192 | 4.310580404 | 3.86E-10 | 1.72E-09 |
| AC131025.1 | 0.04947507 | 0.119166973 | 1.268210809 | 0.00268503 | 0.0042353 |
| AL450344.3 | 0.0571066 | 0.330260323 | 2.531874259 | 0.03392133 | 0.04406218 |
| AL035416.1 | 0.032434269 | 0.230466005 | 2.828963136 | 0.00063724 | 0.00111339 |
| AC009549.1 | 0.255673766 | 0.089656573 | -1.511822876 | 1.34E-07 | 4.11E-07 |
| AL133243.2 | 0.404950484 | 1.318029727 | 1.702565494 | 1.42E-05 | 3.21E-05 |
| AL606537.1 | 0.008121175 | 0.106448861 | 3.71232819 | 0.00023157 | 0.00043111 |
| AL158825.2 | 0.151538777 | 0.414359872 | 1.451197282 | 0.0008002 | 0.00137563 |
| AC006960.3 | 0.034532827 | 0.116741817 | 1.757281077 | 4.01E-05 | 8.42E-05 |
| AC011676.1 | 0.087524561 | 0.655641551 | 2.90514747 | 3.54E-16 | 4.02E-15 |
| AC103691.1 | 0.534035044 | 1.346062264 | 1.333738824 | 1.38E-06 | 3.63E-06 |
| LINC00507 | 0.205659806 | 0.013921614 | -3.884861448 | 1.12E-28 | 2.36E-26 |
| ARAP1-AS2 | 0.066480492 | 0.268108268 | 2.01181275 | 0.00028326 | 0.00052148 |
| AC027373.1 | 0.113449082 | 0.304235156 | 1.423141934 | 1.94E-08 | 6.68E-08 |
| AL592211.1 | 0.125055825 | 0.31659486 | 1.340065572 | 1.27E-06 | 3.38E-06 |
| AC024560.3 | 0.222100405 | 0.519519221 | 1.2259651 | 0.00085408 | 0.00146134 |
| AL020994.1 | 0.177073132 | 0.034928764 | -2.341857834 | 2.11E-19 | 4.33E-18 |
| LINC00896 | 0.118361313 | 0.556870712 | 2.234144816 | 8.01E-14 | 6.21E-13 |
| LINC01914 | 0.055968347 | 0.172741901 | 1.62593502 | 0.00290836 | 0.00455727 |
| AL121796.1 | 0.122965331 | 0.03940508 | -1.641798072 | 2.79E-06 | 7.05E-06 |
| AL139274.2 | 0.038194749 | 0.081616167 | 1.095480638 | 5.94E-05 | 0.00012123 |
| FILNC1 | 0.024599299 | 0.010842447 | -1.181926729 | 5.98E-10 | 2.59E-09 |
| AL592166.1 | 0.170695448 | 0.379815509 | 1.153874232 | 1.39E-10 | 6.67E-10 |
| GATA2-AS1 | 0.254310126 | 1.896411185 | 2.898610995 | 8.28E-14 | 6.40E-13 |
| LINC01081 | 0.411996005 | 0.138525555 | -1.572478195 | 2.03E-18 | 3.39E-17 |
| AC024337.2 | 0.076485371 | 0.02379494 | -1.684528998 | 1.22E-16 | 1.49E-15 |
| BX664727.3 | 0.085154596 | 0.028066056 | -1.601258058 | 9.56E-15 | 8.48E-14 |
| AL445363.2 | 0.080279501 | 0.004250422 | -4.2393537 | 1.13E-38 | 1.54E-35 |
| AC120498.4 | 0.052297744 | 0.547568755 | 3.388219504 | 2.91E-10 | 1.33E-09 |
| MIR1-1HG | 0.068116146 | 0.012897746 | -2.400877879 | 1.61E-17 | 2.28E-16 |
| AC011365.2 | 0.164694657 | 0.042252964 | -1.962669315 | 8.02E-19 | 1.43E-17 |
| LINC00853 | 0.381458931 | 0.910865074 | 1.255709623 | 1.04E-10 | 5.03E-10 |
| AP003170.4 | 0.03829227 | 0.098648673 | 1.36524645 | 0.02629008 | 0.03476232 |
| AC103563.2 | 0.026840836 | 0.009740846 | -1.462310657 | 2.79E-13 | 1.99E-12 |
| AC002511.2 | 1.176472402 | 0.393538162 | -1.579892031 | 1.70E-14 | 1.45E-13 |
| AC005180.1 | 1.008373278 | 0.133998437 | -2.911741712 | 5.68E-22 | 2.18E-20 |
| AC104695.3 | 0.254618022 | 1.916977646 | 2.91242707 | 3.90E-05 | 8.20E-05 |
| AC026401.3 | 3.868348933 | 10.1750567 | 1.395246994 | 1.50E-22 | 6.65E-21 |
| AC016205.1 | 0.233813238 | 0.510945905 | 1.127813942 | 2.42E-05 | 5.27E-05 |
| AC011933.2 | 0.005784138 | 0.236132476 | 5.351350792 | 0.03364467 | 0.04373406 |
| AL158835.2 | 0.131245685 | 0.274234878 | 1.063142081 | 0.00244359 | 0.00387803 |
| VSTM2A-OT1 | 0.051285374 | 0.004694495 | -3.449505505 | 2.24E-47 | 1.22E-43 |
| LINC00955 | 0.544086848 | 0.057720272 | -3.23668695 | 1.59E-11 | 8.71E-11 |
| CATIP-AS1 | 0.079378383 | 0.191389889 | 1.269696542 | 1.47E-05 | 3.31E-05 |
| LINC00891 | 0.061513209 | 0.007447839 | -3.046002555 | 4.73E-25 | 4.45E-23 |
| LINC01411 | 0.006545102 | 0.828644389 | 6.984193609 | 1.19E-16 | 1.46E-15 |
| AC100823.1 | 0.031292534 | 0.267056538 | 3.093254813 | 0.00088209 | 0.00150548 |
| LINC00659 | 0.034970559 | 2.920882332 | 6.384119561 | 5.28E-24 | 3.55E-22 |
| AC011247.1 | 0.211639814 | 0.051701713 | -2.033327054 | 3.89E-09 | 1.48E-08 |
| AC021242.2 | 0.090104592 | 0.019942458 | -2.175757408 | 1.18E-10 | 5.69E-10 |
| AP001469.2 | 0.200292582 | 0.512954249 | 1.356721162 | 4.85E-08 | 1.57E-07 |
| AL022324.3 | 0.028224921 | 0.112977651 | 2.000995978 | 0.00361967 | 0.00557572 |
| SNHG11 | 2.004840793 | 5.111233887 | 1.350183935 | 5.07E-18 | 7.85E-17 |
| AC138393.3 | 0.122667517 | 0.434249309 | 1.82377029 | 0.0038808 | 0.0059494 |
| LINC01840 | 0.07145215 | 0.353596432 | 2.3070544 | 0.00266687 | 0.00420911 |
| AC091729.3 | 0.98822247 | 2.031096951 | 1.039351342 | 6.40E-11 | 3.21E-10 |
| LRP1-AS | 0.059054709 | 0.171954303 | 1.541901221 | 3.64E-06 | 9.04E-06 |
| MRTFA-AS1 | 0.014414479 | 0.26436402 | 4.196935203 | 0.01202011 | 0.01692374 |
| ZNF337-AS1 | 0.144989649 | 0.37453208 | 1.36913939 | 6.15E-12 | 3.59E-11 |
| AL035665.1 | 0.012520478 | 0.032020794 | 1.354719403 | 0.0185914 | 0.02530045 |
| ASMTL-AS1 | 0.876924921 | 2.439482066 | 1.476049642 | 1.72E-09 | 6.91E-09 |
| AF064860.1 | 0.140303806 | 0.052153696 | -1.427712743 | 1.32E-08 | 4.68E-08 |
| LINC01704 | 0.001924875 | 0.04823204 | 4.64715515 | 3.54E-10 | 1.58E-09 |
| AP001363.2 | 0.198895388 | 0.098459473 | -1.0144079 | 1.56E-07 | 4.72E-07 |
| CERS6-AS1 | 0.012706186 | 0.036674475 | 1.529245278 | 2.43E-07 | 7.15E-07 |
| AC011511.3 | 0.031943512 | 0.44701333 | 3.806723021 | 9.38E-11 | 4.59E-10 |
| AC110995.1 | 0.590310327 | 0.089365261 | -2.723687553 | 7.88E-23 | 3.80E-21 |
| AC245884.10 | 0.045458812 | 0.393405967 | 3.113386951 | 0.0010364 | 0.00174698 |
| AP002761.4 | 3.599258861 | 1.432495168 | -1.329169593 | 3.10E-15 | 2.98E-14 |
| AC116049.2 | 0.019760011 | 0.15117822 | 2.935594645 | 5.30E-05 | 0.00010915 |
| AL138999.1 | 0.114583819 | 0.374822621 | 1.709804692 | 0.0003589 | 0.00065257 |
| AL355607.1 | 0.721298039 | 0.107327848 | -2.748571051 | 1.09E-05 | 2.51E-05 |
| TRHDE-AS1 | 0.039363041 | 0.012268001 | -1.68194149 | 7.45E-20 | 1.69E-18 |
| AC105219.1 | 0.032392188 | 0.827396475 | 4.674860987 | 1.19E-17 | 1.74E-16 |
| AL161457.1 | 0.111452142 | 0.034324158 | -1.699128103 | 6.25E-24 | 4.11E-22 |
| SNHG4 | 0.915584946 | 3.578926002 | 1.966761068 | 7.46E-19 | 1.34E-17 |
| AL359853.1 | 0.329522114 | 0.100414166 | -1.714412468 | 7.05E-14 | 5.50E-13 |
| AL096828.1 | 0.025299356 | 0.089765964 | 1.827067872 | 0.02426354 | 0.0323336 |
| AL731569.1 | 0.145440629 | 0.34578482 | 1.249444189 | 8.62E-12 | 4.91E-11 |
| AC106895.2 | 0.017858911 | 0.006096065 | -1.550693808 | 2.25E-10 | 1.04E-09 |
| AL356489.2 | 0.311387523 | 0.045734494 | -2.767356548 | 9.39E-21 | 2.71E-19 |
| LINC02182 | 0.017620445 | 0.120848648 | 2.777879049 | 0.00109419 | 0.00183758 |
| AC124283.2 | 0.322698325 | 0.868960872 | 1.429105125 | 1.67E-11 | 9.11E-11 |
| AL355483.1 | 0.005973746 | 0.208689564 | 5.126578661 | 1.44E-07 | 4.40E-07 |
| AGAP11 | 0.03138161 | 0.014653242 | -1.098699447 | 7.20E-08 | 2.28E-07 |
| CHL1-AS2 | 0.246536314 | 0.0386359 | -2.673786244 | 3.36E-22 | 1.42E-20 |
| AL359881.1 | 0.041211281 | 0.870477458 | 4.400695735 | 3.86E-13 | 2.72E-12 |
| AC245128.3 | 0.115915397 | 0.46671259 | 2.009462177 | 0.00175375 | 0.00285554 |
| LINC02154 | 0.019198579 | 0.134919695 | 2.813029497 | 1.12E-08 | 3.99E-08 |
| AC134407.1 | 0.03349783 | 0.191350698 | 2.514079596 | 0.00048978 | 0.00087082 |
| AL031710.2 | 0.061502226 | 0.361880342 | 2.556802218 | 2.03E-16 | 2.40E-15 |
| GCC2-AS1 | 0.141640547 | 0.323804323 | 1.19288793 | 1.68E-11 | 9.17E-11 |
| WNT5A-AS1 | 0.341277509 | 0.70080695 | 1.038071739 | 0.00581611 | 0.00866064 |
| AC005281.1 | 0.115818785 | 0.056118348 | -1.045324819 | 5.90E-08 | 1.89E-07 |
| LINC01561 | 0.039218721 | 0.124538151 | 1.666973384 | 0.00012486 | 0.0002417 |
| FLJ31356 | 0.086190623 | 0.180439864 | 1.065915283 | 9.70E-05 | 0.00019125 |
| AL645568.1 | 0.641974858 | 0.256328623 | -1.324522211 | 4.42E-19 | 8.43E-18 |
| TARID | 0.113908734 | 0.030568223 | -1.89777378 | 5.50E-22 | 2.13E-20 |
| HOTAIR | 0.023649954 | 0.290550312 | 3.618878694 | 0.00166881 | 0.00273191 |
| AL591178.1 | 0.516353845 | 1.133111032 | 1.133857281 | 0.00065402 | 0.00114014 |
| AL161935.3 | 0.390327285 | 0.177556615 | -1.136405204 | 8.10E-09 | 2.96E-08 |
| AC114730.1 | 0.040099106 | 0.097955225 | 1.288552389 | 0.00180153 | 0.00292286 |
| AP001029.1 | 0.0943905 | 0.376546846 | 1.996115792 | 1.65E-05 | 3.70E-05 |
| MYOSLID | 0.029751432 | 0.111251624 | 1.902795395 | 7.79E-08 | 2.46E-07 |
| AL583722.1 | 0.133417315 | 0.285027226 | 1.095153824 | 4.62E-07 | 1.32E-06 |
| AC106900.2 | 0.293228433 | 1.752170498 | 2.579046258 | 1.73E-19 | 3.65E-18 |
| AC008243.1 | 0.021200095 | 0.200876881 | 3.244168875 | 0.00106552 | 0.0017922 |
| AC007406.3 | 0.044732367 | 0.294344925 | 2.71811675 | 9.31E-18 | 1.39E-16 |
| MAL2-AS1 | 0.069133471 | 0.43857428 | 2.665364937 | 0.00022922 | 0.00042718 |
| GAS1RR | 0.140933413 | 0.034128263 | -2.045974778 | 1.29E-13 | 9.63E-13 |
| AC244453.3 | 0.060751648 | 0.02021993 | -1.587145527 | 5.10E-10 | 2.23E-09 |
| AL354919.2 | 0.02314131 | 0.107447698 | 2.215092111 | 7.01E-05 | 0.00014129 |
| AL390195.1 | 0.162491674 | 0.441463638 | 1.441928814 | 5.42E-10 | 2.37E-09 |
| AL139397.1 | 0.047992658 | 0.158579567 | 1.724321266 | 5.02E-05 | 0.00010365 |
| MDS2 | 0.042491848 | 0.099227718 | 1.223557091 | 6.58E-05 | 0.00013326 |
| AL136309.2 | 0.170878063 | 0.055366676 | -1.625877389 | 1.55E-17 | 2.21E-16 |
| AC113143.1 | 0.062150966 | 0.596735864 | 3.26324377 | 0.0301633 | 0.03951958 |
| LRRC3-DT | 0.092219309 | 0.045406247 | -1.022178069 | 0.00996047 | 0.01418873 |
| AC020558.1 | 0.060010208 | 0.133190559 | 1.150211984 | 0.003947 | 0.006039 |
| AP006545.1 | 0.100743945 | 0.293417352 | 1.542261066 | 1.50E-07 | 4.57E-07 |
| AC026250.1 | 0.168855033 | 0.340795854 | 1.013122606 | 1.95E-06 | 5.03E-06 |
| AC018926.2 | 0.153483277 | 0.526616266 | 1.778670612 | 2.47E-09 | 9.74E-09 |
| AP001347.1 | 0.19004374 | 0.092197746 | -1.043528123 | 6.31E-05 | 0.00012834 |
| AL163953.1 | 0.072160286 | 1.078761929 | 3.902027644 | 2.36E-19 | 4.76E-18 |
| LINC01655 | 0.001925543 | 0.265050499 | 7.104857797 | 4.08E-10 | 1.81E-09 |
| PCAT6 | 1.075000534 | 2.434612086 | 1.179354545 | 2.62E-12 | 1.62E-11 |
| LINC00571 | 0.097476347 | 0.232548185 | 1.254405595 | 0.00036816 | 0.00066786 |
| AC104561.1 | 0.196735798 | 0.085125968 | -1.208588282 | 2.37E-10 | 1.10E-09 |
| AC099066.2 | 0.026777783 | 0.082965592 | 1.631476636 | 0.00192187 | 0.00310518 |
| AL008718.3 | 0.088401456 | 0.312169619 | 1.820188105 | 0.00495425 | 0.00745256 |
| SLC9A3-AS1 | 2.293390483 | 7.769877633 | 1.760409862 | 2.97E-08 | 9.94E-08 |
| SFTA1P | 0.912431989 | 0.187735398 | -2.28101633 | 1.33E-18 | 2.28E-17 |
| MRPL23-AS1 | 0.01043689 | 0.152291126 | 3.867068071 | 0.00011939 | 0.00023159 |
| LINC01094 | 0.23452278 | 0.514097067 | 1.132312716 | 0.00533006 | 0.00798046 |
| AP003774.3 | 0.090821139 | 0.02628511 | -1.788782362 | 5.19E-14 | 4.11E-13 |
| AC010864.1 | 0.095069414 | 0.236790272 | 1.31655663 | 1.98E-11 | 1.07E-10 |
| MATN1-AS1 | 0.200205659 | 0.406745925 | 1.022645138 | 3.88E-11 | 2.01E-10 |
| AP001330.1 | 0.087976371 | 0.009097423 | -3.273586313 | 1.32E-30 | 3.78E-28 |
| AC048341.2 | 0.565233392 | 1.445494258 | 1.354644275 | 6.06E-10 | 2.62E-09 |
| AL024507.2 | 0.111309102 | 0.27040168 | 1.280532548 | 6.05E-16 | 6.61E-15 |
| AL590822.2 | 0.01925189 | 0.058999582 | 1.615704654 | 0.03800096 | 0.04897642 |
| AL353796.1 | 0.267909664 | 0.703111421 | 1.392006707 | 1.09E-14 | 9.48E-14 |
| AC083906.3 | 0.009949563 | 0.111605107 | 3.487626072 | 1.09E-05 | 2.51E-05 |
| AC109479.1 | 0.039368384 | 0.189024264 | 2.263462032 | 2.07E-08 | 7.11E-08 |
| AC020922.3 | 0.093093215 | 0.19132085 | 1.039246175 | 0.01143115 | 0.01614035 |
| AL031985.3 | 0.455716381 | 1.220688368 | 1.421486804 | 9.53E-18 | 1.42E-16 |
| MYB-AS1 | 0.093654657 | 0.437697672 | 2.224512073 | 0.00386977 | 0.00593416 |
| PCAT18 | 0.174303205 | 0.027385197 | -2.670130959 | 3.02E-25 | 3.11E-23 |
| LINC01138 | 0.406821766 | 0.876734361 | 1.107742921 | 6.14E-13 | 4.20E-12 |
| LINC01749 | 0.003424772 | 0.031163088 | 3.18575841 | 0.00025027 | 0.00046373 |
| AC100814.1 | 0.763822502 | 1.547545969 | 1.018672937 | 4.58E-06 | 1.12E-05 |
| DUXAP8 | 0.012408747 | 0.190620571 | 3.941274418 | 1.32E-15 | 1.37E-14 |
| AC092614.1 | 0.126494288 | 0.47455846 | 1.90751358 | 1.27E-16 | 1.55E-15 |
| AC005096.1 | 0.019687163 | 0.286371723 | 3.862561925 | 1.31E-06 | 3.48E-06 |
| AC126177.4 | 0.038795698 | 0.015070962 | -1.364125148 | 5.70E-12 | 3.34E-11 |
| AC020656.2 | 0.03527375 | 0.612516164 | 4.118081044 | 1.29E-16 | 1.57E-15 |
| AL121987.1 | 0.191842609 | 0.051879645 | -1.88668267 | 5.21E-15 | 4.81E-14 |
| AC144450.1 | 0.009705198 | 0.197239268 | 4.345045297 | 7.95E-09 | 2.91E-08 |
| AL445222.1 | 0.183977636 | 0.692916806 | 1.913151747 | 1.22E-12 | 7.94E-12 |
| AL121845.4 | 0.160652924 | 0.471351735 | 1.552856797 | 0.00404473 | 0.00618159 |
| AC012065.3 | 0.183916484 | 0.431149894 | 1.229138736 | 0.00179497 | 0.00291395 |
| AC107032.2 | 0.099146601 | 0.314734812 | 1.666501537 | 6.99E-10 | 3.00E-09 |
| AC023510.2 | 0.254519431 | 0.112382766 | -1.179354986 | 1.83E-12 | 1.16E-11 |
| AF001548.1 | 1.496371379 | 0.188335476 | -2.990091588 | 4.39E-14 | 3.50E-13 |
| LINC01600 | 0.01027928 | 0.027073552 | 1.397144935 | 0.0065865 | 0.00969921 |
| AC010247.2 | 0.206702065 | 0.076278721 | -1.438200245 | 1.05E-14 | 9.25E-14 |
| LINC02166 | 0.295594476 | 0.618775989 | 1.065797907 | 1.08E-09 | 4.46E-09 |
| CADM3-AS1 | 0.181013114 | 0.048321404 | -1.905359952 | 1.52E-17 | 2.17E-16 |
| MIR9-3HG | 0.096663266 | 0.048084654 | -1.007391213 | 1.10E-13 | 8.28E-13 |
| AC127024.5 | 0.672906227 | 2.231333376 | 1.729428701 | 1.04E-12 | 6.91E-12 |
| LINC01502 | 0.072267333 | 0.033730514 | -1.099289345 | 0.00618171 | 0.00915255 |
| AC087289.2 | 0.129630913 | 0.329038622 | 1.343847133 | 1.84E-12 | 1.17E-11 |
| LINC00877 | 0.111656615 | 0.045914416 | -1.282049629 | 1.40E-13 | 1.03E-12 |
| LINC01978 | 0.130730887 | 1.724009037 | 3.721095392 | 4.22E-20 | 1.04E-18 |
| LINC01269 | 0.043240894 | 0.368422286 | 3.090892093 | 1.04E-10 | 5.03E-10 |
| IRAIN | 0.10889076 | 0.262313105 | 1.268408349 | 0.00035268 | 0.00064213 |
| AL355312.3 | 0.217393327 | 5.5105239 | 4.663809924 | 1.15E-21 | 4.03E-20 |
| AC007349.2 | 0.051912959 | 0.014632176 | -1.826950394 | 1.78E-14 | 1.51E-13 |
| AC004594.1 | 0.01802622 | 0.146986102 | 3.027510955 | 0.00227109 | 0.00362218 |
| AC073352.1 | 0.090723012 | 0.239619682 | 1.401205967 | 1.15E-05 | 2.62E-05 |
| AC129492.1 | 0.1836076 | 0.458184916 | 1.319304189 | 0.00913805 | 0.01309932 |
| AL034417.2 | 0.024873055 | 0.085412101 | 1.779856786 | 2.35E-08 | 8.00E-08 |
| AC254562.2 | 0.032221927 | 0.131725967 | 2.031425069 | 1.74E-06 | 4.51E-06 |
| B4GALT1-AS1 | 3.44281881 | 1.137858258 | -1.597269402 | 6.82E-22 | 2.58E-20 |
| LINC00163 | 0.035910907 | 0.0138409 | -1.375484363 | 2.60E-12 | 1.61E-11 |
| LINC01747 | 0.002677244 | 0.02117591 | 2.983603401 | 8.06E-09 | 2.95E-08 |
| AC005699.1 | 0.145180924 | 0.058594803 | -1.309007296 | 1.61E-12 | 1.02E-11 |
| AC104211.1 | 0.088819833 | 0.018794733 | -2.240553438 | 1.12E-22 | 5.16E-21 |
| SNHG12 | 1.838034185 | 5.001035136 | 1.444063142 | 4.01E-19 | 7.72E-18 |
| ATXN2-AS | 0.257017761 | 0.552850374 | 1.105021014 | 3.66E-10 | 1.63E-09 |
| AL049780.1 | 0.157027875 | 0.333188033 | 1.085315907 | 0.00056834 | 0.00100116 |
| AC100778.3 | 0.090177984 | 0.192518022 | 1.094146335 | 0.00177468 | 0.00288445 |
| C5orf66-AS1 | 0.888336219 | 0.30708279 | -1.532478153 | 1.58E-15 | 1.60E-14 |
| LINC00893 | 0.209482986 | 0.446001636 | 1.090215928 | 2.21E-05 | 4.87E-05 |
| LINC02097 | 0.02301545 | 0.083943717 | 1.866819705 | 0.00090017 | 0.00153298 |
| DCUN1D2-AS | 0.137058446 | 0.363344034 | 1.406544987 | 0.00120953 | 0.00201391 |
| AC091057.4 | 0.135480178 | 0.758106395 | 2.484318548 | 8.59E-11 | 4.24E-10 |
| OGFRP1 | 0.40204818 | 0.831098438 | 1.047650966 | 2.68E-14 | 2.21E-13 |
| AL158198.1 | 0.163834905 | 0.048611206 | -1.752881921 | 1.97E-16 | 2.33E-15 |
| LINC02577 | 0.004603297 | 0.576222351 | 6.967814438 | 4.07E-25 | 3.96E-23 |
| AC090877.2 | 0.035943103 | 0.148726181 | 2.048871755 | 0.00052366 | 0.00092531 |
| AC138932.5 | 0.225476097 | 0.563903702 | 1.322474312 | 0.00702413 | 0.01027429 |
| AC018552.3 | 0.015273575 | 0.254663073 | 4.059480128 | 9.75E-05 | 0.00019197 |
| CCDC144NL-AS1 | 0.037481395 | 0.109796182 | 1.550581332 | 2.09E-05 | 4.60E-05 |
| POC1B-AS1 | 0.185273444 | 0.511938956 | 1.466315686 | 2.21E-16 | 2.59E-15 |
| AC090578.2 | 0.035086906 | 0.225795856 | 2.686014369 | 0.00259032 | 0.00409658 |
| AC020978.3 | 0.139708916 | 0.570501276 | 2.029806016 | 0.00089227 | 0.00152049 |
| AL121895.2 | 0.343230974 | 0.686794675 | 1.000699103 | 0.01219328 | 0.017141 |
| AL031283.2 | 0.017442634 | 0.004308601 | -2.017326419 | 7.35E-07 | 2.02E-06 |
| HORMAD2-AS1 | 0.081211297 | 0.025625265 | -1.664113482 | 2.76E-17 | 3.77E-16 |
| AC010967.1 | 0.001143657 | 0.160274444 | 7.130745948 | 5.40E-11 | 2.74E-10 |
| SMIM25 | 0.658907642 | 1.782329778 | 1.435616134 | 1.18E-12 | 7.75E-12 |
| AC005736.1 | 0.20324892 | 0.099123915 | -1.035942615 | 3.48E-10 | 1.56E-09 |
| AL034550.1 | 0.38183191 | 0.800712627 | 1.068346881 | 0.00173168 | 0.00282213 |
| AC022819.1 | 0.022306811 | 0.052100047 | 1.223800416 | 0.00332788 | 0.00515978 |
| AC092834.1 | 2.058388184 | 0.229781397 | -3.16318117 | 1.70E-23 | 1.03E-21 |
| AC079313.2 | 0.576363317 | 0.059297389 | -3.280938028 | 5.11E-10 | 2.24E-09 |
| AC025154.2 | 0.005856411 | 0.420977556 | 6.167582724 | 1.16E-19 | 2.53E-18 |
| LINC02033 | 0.230028977 | 0.057378823 | -2.003225328 | 1.01E-19 | 2.21E-18 |
| LINC02542 | 0.655820144 | 1.695284026 | 1.37015488 | 0.00057302 | 0.00100728 |
| AC116337.3 | 0.039689763 | 0.013406478 | -1.565836666 | 6.26E-10 | 2.70E-09 |
| AC103740.1 | 0.358111994 | 0.109476033 | -1.709795777 | 3.73E-18 | 5.91E-17 |
| AC245884.9 | 0.033819094 | 0.131862071 | 1.963119743 | 0.02617794 | 0.03464765 |
| AC243654.1 | 0.03158814 | 0.092206773 | 1.545489759 | 0.00660509 | 0.00972133 |
| AC006042.1 | 1.647802138 | 3.766569159 | 1.192708 | 6.02E-07 | 1.68E-06 |
| AC007342.5 | 0.534850718 | 1.337549659 | 1.322384272 | 1.70E-08 | 5.93E-08 |
| AL137244.1 | 0.106504511 | 0.477298795 | 2.163978151 | 8.38E-11 | 4.14E-10 |
| AC005532.1 | 0.096285103 | 0.20765355 | 1.10879403 | 0.01428847 | 0.01987629 |
| AC024560.1 | 0.088491933 | 0.178720219 | 1.01408501 | 0.00432409 | 0.00657904 |
| AC008734.1 | 0.035171764 | 0.112151131 | 1.672954574 | 0.00013929 | 0.00026784 |
| LINC01614 | 0.015179692 | 0.985324679 | 6.020384806 | 1.77E-17 | 2.48E-16 |
| ZMIZ1-AS1 | 0.099148629 | 0.231889758 | 1.225774366 | 1.07E-08 | 3.84E-08 |
| SPATA3-AS1 | 0.049542151 | 0.257664622 | 2.37876604 | 1.19E-17 | 1.74E-16 |
| AC103923.1 | 0.344919372 | 0.134683172 | -1.35668955 | 2.33E-18 | 3.83E-17 |
| AC004691.1 | 0.029502412 | 0.206662605 | 2.80837257 | 0.02196343 | 0.0295137 |
| HCG14 | 0.086388513 | 0.17900637 | 1.051099528 | 0.01558477 | 0.02149311 |
| AC006160.1 | 0.072285957 | 0.190912297 | 1.401122736 | 0.01579752 | 0.02175351 |
| AL121895.1 | 0.029503556 | 0.253324363 | 3.102025091 | 1.09E-06 | 2.92E-06 |
| AC092809.2 | 0.152441038 | 0.063611727 | -1.260886669 | 8.03E-10 | 3.41E-09 |
| AC138028.6 | 0.149643943 | 0.307776669 | 1.040349989 | 1.33E-08 | 4.71E-08 |
| LINC01555 | 0.040999286 | 0.345282353 | 3.074105924 | 1.25E-12 | 8.12E-12 |
| CERS3-AS1 | 0.054529443 | 0.011900614 | -2.195999433 | 3.15E-15 | 3.02E-14 |
| AC243967.2 | 0.093709091 | 0.638051093 | 2.767411039 | 6.26E-07 | 1.74E-06 |
| LINC01191 | 0.026589416 | 0.072252528 | 1.442195965 | 0.00095168 | 0.00161416 |
| AC116351.2 | 0.080430463 | 0.360173548 | 2.162878298 | 1.10E-10 | 5.33E-10 |
| AP000786.1 | 0.206335639 | 0.649539694 | 1.654424667 | 0.01963132 | 0.02655658 |
| AL136162.1 | 0.189925697 | 0.431474435 | 1.183839964 | 2.40E-08 | 8.15E-08 |
| AC079766.1 | 0.109428605 | 0.228109685 | 1.059737791 | 0.00129613 | 0.00214956 |
| AL162424.1 | 0.982105925 | 0.278916407 | -1.816045831 | 4.44E-17 | 5.85E-16 |
| AC123768.2 | 0.063154834 | 0.138192253 | 1.129711665 | 0.00033131 | 0.00060524 |
| AC090739.1 | 0.357680271 | 1.215732848 | 1.765083789 | 0.00015234 | 0.00029134 |
| CARS-AS1 | 0.007633457 | 0.071174872 | 3.220959596 | 5.06E-06 | 1.23E-05 |
| AC008759.3 | 0.095996904 | 0.040184389 | -1.256352734 | 1.27E-07 | 3.91E-07 |
| AC016355.1 | 1.507963536 | 0.688209896 | -1.131681001 | 3.29E-18 | 5.22E-17 |
| AC017002.3 | 0.07660015 | 0.243982762 | 1.671360105 | 5.59E-06 | 1.35E-05 |
| AC016394.1 | 0.473160277 | 1.638365452 | 1.791856333 | 4.39E-17 | 5.80E-16 |
| AC244093.4 | 0.04735709 | 0.289502163 | 2.6119218 | 0.00941442 | 0.01346717 |
| AC091435.2 | 0.091827753 | 0.022679761 | -2.017524784 | 6.42E-20 | 1.49E-18 |
| STX18-AS1 | 0.219853152 | 0.455842397 | 1.051994894 | 7.05E-15 | 6.38E-14 |
| LINC02100 | 0.119168874 | 0.482965862 | 2.018913753 | 2.04E-06 | 5.25E-06 |
| MIR100HG | 1.459197919 | 0.71398626 | -1.031207361 | 1.45E-06 | 3.82E-06 |
| NARF-IT1 | 0.189100761 | 0.401815202 | 1.087376979 | 0.00026229 | 0.00048451 |
| AL391421.1 | 0.006703023 | 0.111750922 | 4.059330938 | 1.86E-05 | 4.13E-05 |
| CCAT2 | 0.004971478 | 0.225422448 | 5.502812478 | 1.39E-09 | 5.69E-09 |
| AL162727.1 | 0.003875583 | 0.026481109 | 2.772478428 | 6.56E-09 | 2.44E-08 |
| LINC01311 | 0.249070231 | 0.831852344 | 1.739774868 | 1.36E-17 | 1.97E-16 |
| AC004832.4 | 0.083746606 | 0.343964312 | 2.038156263 | 0.02006425 | 0.02710189 |
| AC109322.1 | 0.627479295 | 1.285052205 | 1.034187209 | 5.32E-13 | 3.69E-12 |
| AP002358.1 | 0.09427842 | 0.016895684 | -2.480272834 | 5.12E-19 | 9.59E-18 |
| AC093895.1 | 0.001748253 | 0.090872652 | 5.699860273 | 7.83E-08 | 2.47E-07 |
| AC104971.3 | 0.400704217 | 0.084858063 | -2.239414036 | 2.25E-17 | 3.12E-16 |
| AL133330.1 | 0.125802169 | 0.7331031 | 2.542859314 | 5.09E-13 | 3.55E-12 |
| AC104819.3 | 0.823193132 | 0.310535984 | -1.406470491 | 2.94E-15 | 2.86E-14 |
| AL132780.2 | 0.120777299 | 0.282051685 | 1.22361024 | 1.37E-09 | 5.59E-09 |
| AC023355.1 | 0.048109214 | 0.098973429 | 1.040728042 | 1.72E-05 | 3.85E-05 |
| LINC01569 | 0.568519192 | 1.545912435 | 1.443177646 | 9.50E-17 | 1.19E-15 |
| AP002907.1 | 0.200089713 | 0.68913778 | 1.784145455 | 8.89E-08 | 2.78E-07 |
| LINC02147 | 0.080645327 | 0.012678106 | -2.669251681 | 3.70E-30 | 9.18E-28 |
| AF127936.1 | 0.469062613 | 0.158712328 | -1.56336632 | 2.88E-15 | 2.81E-14 |
| AC010542.5 | 1.054460006 | 2.677765167 | 1.344525068 | 1.86E-12 | 1.17E-11 |
| C12orf77 | 0.016387893 | 0.005953055 | -1.46092824 | 1.36E-08 | 4.82E-08 |
| LINC02323 | 0.370520831 | 0.812800446 | 1.133346538 | 8.49E-07 | 2.31E-06 |
| AL139035.1 | 0.166206717 | 0.403161819 | 1.278380332 | 1.41E-09 | 5.73E-09 |
| AC022211.1 | 0.413967639 | 0.902391935 | 1.124236182 | 0.00047691 | 0.00085079 |
| RERG-IT1 | 0.184204976 | 0.060672619 | -1.60219455 | 1.51E-11 | 8.34E-11 |
| LINC00836 | 0.019041589 | 0.002581669 | -2.882778045 | 3.29E-31 | 9.98E-29 |
| BX284668.2 | 0.161105396 | 0.05320996 | -1.598236599 | 5.11E-10 | 2.24E-09 |
| LINC01550 | 0.221157057 | 0.0889138 | -1.314592026 | 1.49E-16 | 1.80E-15 |
| AL109936.2 | 1.833307775 | 0.811131329 | -1.176441582 | 3.38E-15 | 3.23E-14 |
| AL031670.1 | 0.355364825 | 0.83976812 | 1.240690132 | 0.00082141 | 0.0014103 |
| HDAC2-AS2 | 0.064863698 | 0.147055873 | 1.180881223 | 1.67E-10 | 7.89E-10 |
| AL121832.2 | 1.137982249 | 3.291242913 | 1.532154456 | 1.28E-15 | 1.33E-14 |
| AC120049.1 | 0.399026797 | 0.148615677 | -1.424899327 | 6.17E-19 | 1.14E-17 |
| AC148477.4 | 0.017605248 | 0.148786538 | 3.079166524 | 0.00318397 | 0.00495638 |
| AC048382.1 | 0.029499346 | 0.088937891 | 1.592115222 | 0.00046921 | 0.00083833 |
| UNC5B-AS1 | 0.172395701 | 0.821859406 | 2.253167821 | 0.00189086 | 0.00305869 |
| AL121790.2 | 3.915036583 | 0.324193963 | -3.594096657 | 3.12E-24 | 2.36E-22 |
| MPRIP-AS1 | 0.01124515 | 0.185533378 | 4.044303913 | 0.00436943 | 0.00664061 |
| AC145285.6 | 0.207065738 | 0.600973773 | 1.537213172 | 1.54E-12 | 9.84E-12 |
| AC090044.1 | 0.146115915 | 0.042066025 | -1.796385909 | 2.36E-06 | 6.02E-06 |
| AC087379.1 | 9.416378902 | 0.383645309 | -4.617327355 | 2.13E-23 | 1.25E-21 |
| LINC01230 | 0.063114016 | 0.030700719 | -1.039687984 | 3.57E-16 | 4.05E-15 |
| AP005271.1 | 0.029482264 | 0.651164914 | 4.465103755 | 2.04E-09 | 8.13E-09 |
| AL662797.1 | 0.232944653 | 0.48810019 | 1.067190096 | 7.92E-08 | 2.50E-07 |
| Z84485.1 | 0.125286283 | 0.405695098 | 1.695167406 | 3.04E-11 | 1.60E-10 |
| LINC00184 | 0.005527844 | 0.027112614 | 2.294175518 | 0.00010203 | 0.00020063 |
| AC012085.2 | 1.49469724 | 0.222506365 | -2.747934774 | 8.21E-15 | 7.37E-14 |
| AL161431.1 | 0.037989587 | 2.568214087 | 6.079017647 | 1.42E-18 | 2.43E-17 |
| LINC01022 | 0.098262698 | 0.034840799 | -1.495866138 | 4.04E-11 | 2.09E-10 |
| SND1-IT1 | 0.004778906 | 0.304888176 | 5.995455921 | 0.0079474 | 0.01152277 |
| AL031770.1 | 0.014050467 | 0.169474531 | 3.592378472 | 5.18E-06 | 1.25E-05 |
| ARHGAP27P1-BPTFP1-KPNA2P3 | 0.70975153 | 1.508839106 | 1.088053014 | 2.09E-07 | 6.20E-07 |
| AC005865.1 | 0.01296292 | 0.055314516 | 2.093267417 | 9.77E-06 | 2.26E-05 |
| AC005632.2 | 0.181297233 | 0.480074131 | 1.404900287 | 0.01573682 | 0.02169183 |
| AC004982.1 | 0.19223431 | 0.389971483 | 1.020502776 | 6.72E-08 | 2.14E-07 |
| MIR4435-2HG | 0.635678642 | 1.978756248 | 1.638224384 | 5.17E-22 | 2.03E-20 |
| AP002360.3 | 0.448083892 | 1.304715084 | 1.541894024 | 2.85E-09 | 1.11E-08 |
| AC016737.1 | 0.147152507 | 0.502280893 | 1.771182278 | 3.09E-11 | 1.62E-10 |
| AL121832.3 | 0.227277314 | 0.799286987 | 1.814259917 | 4.93E-15 | 4.59E-14 |
| AC011773.4 | 0.018354995 | 0.069973157 | 1.930628887 | 5.92E-06 | 1.42E-05 |
| LENG8-AS1 | 0.99799773 | 2.369775942 | 1.247642223 | 1.54E-14 | 1.32E-13 |
| MIR22HG | 11.86316575 | 3.898286603 | -1.605576985 | 3.33E-22 | 1.42E-20 |
| AC120498.10 | 1.209566117 | 0.539265992 | -1.165420672 | 3.44E-13 | 2.44E-12 |
| LINC00412 | 0.053335106 | 0.291413862 | 2.449912161 | 1.94E-05 | 4.29E-05 |
| AL031005.1 | 0.016160598 | 0.159222238 | 3.300489354 | 0.0172046 | 0.02355428 |
| AC139722.1 | 0.033198722 | 0.014772445 | -1.168219101 | 1.08E-10 | 5.23E-10 |
| AC092652.2 | 0.351512377 | 0.044652146 | -2.976774058 | 6.06E-33 | 2.36E-30 |
| AC016949.1 | 0.114092995 | 0.498428577 | 2.127176572 | 0.00236588 | 0.00376236 |
| AC107081.2 | 0.118880041 | 0.445774904 | 1.906808883 | 5.16E-14 | 4.09E-13 |
| AC112496.1 | 0.205385716 | 1.018120789 | 2.309500977 | 0.00023457 | 0.00043626 |
| AP007216.2 | 0.03139948 | 0.112847 | 1.845555492 | 0.00099802 | 0.00168698 |
| AC002401.1 | 0.022176238 | 0.08790238 | 1.986887603 | 0.00447801 | 0.00679791 |
| LINC02126 | 0.021037254 | 0.068950559 | 1.712615853 | 0.00178057 | 0.0028923 |
| SERTAD4-AS1 | 2.838923877 | 1.034099484 | -1.45696918 | 1.18E-08 | 4.22E-08 |
| LINC02132 | 0.130300285 | 0.039944969 | -1.705754538 | 1.57E-15 | 1.60E-14 |
| LINC00941 | 0.023853111 | 0.631167164 | 4.725772821 | 2.59E-15 | 2.54E-14 |
| AL078604.2 | 0.033002821 | 0.102429992 | 1.633976953 | 0.00026848 | 0.00049561 |
| LINC01915 | 0.380286559 | 0.16752486 | -1.182711743 | 8.61E-14 | 6.60E-13 |
| LINC01351 | 0.6988804 | 0.107002107 | -2.707406385 | 2.14E-29 | 4.66E-27 |
| AC131934.1 | 0.04976213 | 0.200076598 | 2.007432273 | 0.01517611 | 0.02098791 |
| AC127164.1 | 0.010696779 | 0.11479305 | 3.423786952 | 5.88E-09 | 2.20E-08 |
| AC093520.1 | 0.008483613 | 0.076414375 | 3.171093289 | 6.39E-08 | 2.04E-07 |
| AP000873.3 | 0.019981482 | 0.054585931 | 1.449865594 | 0.03422386 | 0.0444234 |
| COL4A2-AS2 | 0.005393967 | 0.078926519 | 3.871091541 | 4.57E-05 | 9.51E-05 |
| AC068580.2 | 0.003572555 | 0.42295971 | 6.887420318 | 6.33E-10 | 2.73E-09 |
| AC005479.2 | 0.082123599 | 0.278534615 | 1.761987862 | 1.26E-06 | 3.35E-06 |
| AC007966.1 | 0.038636037 | 0.23767165 | 2.620950791 | 2.26E-06 | 5.79E-06 |
| AC156455.1 | 0.511762989 | 1.475646 | 1.527798949 | 9.45E-06 | 2.19E-05 |
| AC020900.1 | 0.049899786 | 0.345626683 | 2.792109076 | 0.00545162 | 0.00815351 |
| AC084125.4 | 0.397897523 | 1.037090719 | 1.382073277 | 1.09E-06 | 2.93E-06 |
| AC005775.1 | 0.075045442 | 0.188002078 | 1.324912257 | 2.31E-08 | 7.86E-08 |
| AC007014.2 | 0.056872511 | 0.330808802 | 2.540194226 | 1.84E-07 | 5.52E-07 |
| AC037487.4 | 0.024444999 | 0.101088316 | 2.048004991 | 8.85E-05 | 0.00017565 |
| AC004982.2 | 0.623006892 | 1.362872409 | 1.129330477 | 8.27E-13 | 5.55E-12 |
| LINC01091 | 0.0865578 | 0.311390205 | 1.846987829 | 6.96E-12 | 4.02E-11 |
| AC011731.1 | 0.078207796 | 0.165705348 | 1.083235839 | 0.00096565 | 0.0016343 |
| AC025259.3 | 0.543163838 | 1.126140865 | 1.051927963 | 0.02742189 | 0.03618867 |
| AC010761.4 | 0.176073173 | 0.412990664 | 1.229934056 | 1.38E-06 | 3.64E-06 |
| BX640514.2 | 0.05310147 | 0.325221424 | 2.614598587 | 2.47E-08 | 8.37E-08 |
| AC022390.1 | 0.028491065 | 0.084260347 | 1.564344316 | 0.02760707 | 0.03641542 |
| AL020997.2 | 0.083226744 | 0.456020788 | 2.453980496 | 0.00368163 | 0.00566637 |
| AL137145.2 | 0.04797074 | 0.249484793 | 2.378725267 | 5.59E-09 | 2.09E-08 |
| CASC19 | 0.126995687 | 4.318680885 | 5.087739312 | 5.91E-25 | 5.37E-23 |
| AC004080.1 | 0.0153192 | 0.744339471 | 5.602547868 | 2.01E-10 | 9.37E-10 |
| AC012615.1 | 0.915481903 | 1.876508588 | 1.03544762 | 5.76E-13 | 3.97E-12 |
| AC145124.1 | 0.27190887 | 0.123667372 | -1.136658304 | 3.12E-12 | 1.91E-11 |
| LINC02099 | 0.010664284 | 0.029359772 | 1.461053679 | 4.90E-06 | 1.19E-05 |
| AP000866.6 | 0.142414481 | 0.554145034 | 1.960167771 | 1.55E-06 | 4.06E-06 |
| AC109460.1 | 0.121141395 | 0.286203634 | 1.240350067 | 2.09E-09 | 8.32E-09 |
| AL354696.1 | 0.571796787 | 1.316014764 | 1.202601256 | 1.54E-10 | 7.32E-10 |
| INHBA-AS1 | 0.004925325 | 0.055840759 | 3.503027836 | 2.00E-10 | 9.31E-10 |
| AL662890.1 | 0.025583728 | 0.245109237 | 3.260126447 | 5.27E-13 | 3.66E-12 |
| C2orf48 | 0.082118372 | 0.531582983 | 2.694517996 | 3.95E-18 | 6.22E-17 |
| AC104667.2 | 0.320342725 | 0.786346887 | 1.29554965 | 1.21E-09 | 4.98E-09 |
| IL6R-AS1 | 0.157169493 | 0.044706454 | -1.813766206 | 8.68E-21 | 2.54E-19 |
| AC012173.1 | 0.102046173 | 0.262212286 | 1.361513204 | 8.39E-06 | 1.96E-05 |
| HID1-AS1 | 0.189283302 | 0.07818148 | -1.275648345 | 4.01E-12 | 2.42E-11 |
| FMR1-IT1 | 0.358469437 | 0.808231983 | 1.172919321 | 0.00667695 | 0.00981386 |
| AL158212.2 | 0.142294484 | 0.321204331 | 1.174611606 | 0.01860391 | 0.02531116 |
| AP000802.1 | 0.050564231 | 0.010729898 | -2.236480873 | 2.27E-19 | 4.61E-18 |
| AC103706.1 | 0.157795905 | 0.809893685 | 2.359672767 | 1.25E-13 | 9.30E-13 |
| AC011462.4 | 0.34153104 | 0.855497175 | 1.324746386 | 3.82E-10 | 1.70E-09 |
| AC005091.1 | 0.021220679 | 0.066589801 | 1.649830415 | 0.00048557 | 0.00086417 |
| AP001554.1 | 3.969323966 | 0.443715175 | -3.161187517 | 5.03E-24 | 3.43E-22 |
| LINC00658 | 0.004754978 | 0.067642913 | 3.830428147 | 2.42E-05 | 5.28E-05 |
| AL022318.1 | 0.546563002 | 0.081562618 | -2.744407812 | 2.09E-20 | 5.52E-19 |
| AC025287.2 | 0.044731956 | 0.131001496 | 1.550205542 | 8.65E-07 | 2.35E-06 |
| AL354993.1 | 0.037839029 | 0.147297319 | 1.960784218 | 0.00721378 | 0.01052911 |
| AL589765.6 | 0.099503395 | 0.231067383 | 1.215495973 | 0.00012178 | 0.00023599 |
| AC120498.2 | 0.48485586 | 0.14087647 | -1.783125257 | 4.37E-08 | 1.43E-07 |
| LINC01970 | 0.037586868 | 0.07999246 | 1.089635315 | 0.00015273 | 0.00029192 |
| AL355488.1 | 0.740985289 | 1.656777593 | 1.160863141 | 4.99E-06 | 1.21E-05 |
| AC023906.2 | 0.079054211 | 0.012897031 | -2.615803359 | 8.06E-22 | 2.97E-20 |
| AC010776.3 | 0.098701247 | 0.009535045 | -3.371756645 | 3.32E-27 | 5.49E-25 |
| AL137145.1 | 0.049195479 | 0.201854946 | 2.036721308 | 0.0005559 | 0.00098069 |
| AC016027.1 | 3.470991817 | 0.706858609 | -2.295854394 | 4.58E-23 | 2.40E-21 |
| AL356215.1 | 0.059396598 | 0.176008923 | 1.567196353 | 2.27E-08 | 7.74E-08 |
| C5orf66 | 0.11688086 | 0.317633256 | 1.442323278 | 8.74E-19 | 1.52E-17 |
| AL121782.1 | 0.060506873 | 0.202310283 | 1.741398722 | 2.59E-05 | 5.61E-05 |
| LINC02148 | 0.01440047 | 0.006138248 | -1.230217029 | 2.14E-12 | 1.33E-11 |
| MED4-AS1 | 0.125778414 | 0.349396112 | 1.473979203 | 3.19E-06 | 7.99E-06 |
| AC099811.1 | 0.016077525 | 0.26872387 | 4.063007204 | 0.00053479 | 0.00094376 |
| AC087045.2 | 0.055876636 | 0.022643916 | -1.303121692 | 2.70E-09 | 1.06E-08 |
| PAN3-AS1 | 0.483230489 | 1.079128755 | 1.15908362 | 1.56E-08 | 5.46E-08 |
| AC108865.1 | 0.935892261 | 5.100003241 | 2.446083802 | 0.00568939 | 0.0084812 |
| RTCA-AS1 | 0.987244764 | 0.468985372 | -1.073864888 | 5.51E-14 | 4.35E-13 |
| AC006252.1 | 0.113401802 | 0.277708616 | 1.292128376 | 1.96E-10 | 9.18E-10 |
| AC010273.2 | 0.03648186 | 0.168237243 | 2.205245933 | 1.88E-05 | 4.18E-05 |
| AC211476.2 | 0.049806255 | 0.364343269 | 2.870899511 | 5.05E-08 | 1.64E-07 |
| AC004943.1 | 0.095252764 | 0.313057617 | 1.716595336 | 2.01E-07 | 5.99E-07 |
| DKFZP434K028 | 0.015864084 | 0.106629526 | 2.748770853 | 3.48E-07 | 1.01E-06 |
| LINC00365 | 0.150854666 | 0.435549906 | 1.529678715 | 2.24E-08 | 7.65E-08 |
| AL157400.4 | 0.052098511 | 0.121997809 | 1.2275412 | 0.00963548 | 0.01376124 |
| AL157400.2 | 0.041040001 | 0.116925292 | 1.510484379 | 1.38E-05 | 3.13E-05 |
| USP30-AS1 | 5.382795307 | 2.197533865 | -1.292470167 | 7.97E-11 | 3.95E-10 |
| AC039056.2 | 0.165562818 | 0.409138491 | 1.305210558 | 4.85E-06 | 1.18E-05 |
| PGM5P3-AS1 | 0.026454939 | 0.009526899 | -1.473458495 | 1.57E-13 | 1.15E-12 |
| AL356299.2 | 0.105481173 | 0.815907798 | 2.951420606 | 3.22E-19 | 6.33E-18 |
| AC104785.1 | 0.150571861 | 0.478543554 | 1.668198054 | 1.22E-12 | 7.95E-12 |
| AC005757.1 | 0.019985113 | 0.155897921 | 2.963604024 | 0.00664039 | 0.00976802 |
| LINC01276 | 0.009191739 | 0.070950325 | 2.948399511 | 0.00010555 | 0.00020682 |
| LINC02308 | 0.098441001 | 0.046702818 | -1.075749744 | 5.23E-09 | 1.96E-08 |
| AC104407.1 | 0.239153534 | 0.01537255 | -3.959508673 | 3.51E-30 | 9.10E-28 |
| AC008635.1 | 0.029926023 | 0.153390643 | 2.357738028 | 6.85E-07 | 1.90E-06 |
| UBXN10-AS1 | 4.344908721 | 0.688945145 | -2.656864847 | 2.58E-10 | 1.18E-09 |
| AC105339.3 | 0.049784984 | 0.170067233 | 1.772322637 | 0.00977381 | 0.01393375 |
| LINC00930 | 0.204290642 | 0.09170821 | -1.155500318 | 3.06E-15 | 2.95E-14 |
| MIR503HG | 0.09886459 | 0.927702573 | 3.230136548 | 1.21E-19 | 2.61E-18 |
| AL133520.1 | 0.779760323 | 1.569913801 | 1.009582696 | 5.16E-07 | 1.46E-06 |
| AP000697.1 | 0.001356572 | 0.365124499 | 8.072279456 | 1.77E-16 | 2.12E-15 |
| LINC00460 | 0.012473994 | 1.834157059 | 7.200049871 | 4.18E-24 | 2.96E-22 |
| DPP10-AS1 | 3.621792639 | 0.747899901 | -2.275786851 | 4.38E-19 | 8.37E-18 |
| AC073257.2 | 0.024986253 | 0.148463066 | 2.570897589 | 2.00E-07 | 5.97E-07 |
| AC084859.1 | 0.123983961 | 0.416041389 | 1.746573562 | 4.28E-06 | 1.05E-05 |
| AC116345.3 | 0.250569403 | 0.028120413 | -3.155520558 | 4.26E-23 | 2.28E-21 |
| AC093904.2 | 0.02964202 | 0.208575763 | 2.814855826 | 6.14E-09 | 2.29E-08 |
| LINC00513 | 0.656762188 | 2.245088337 | 1.773329237 | 1.12E-08 | 4.01E-08 |
| AL049794.1 | 0.17509398 | 0.370562374 | 1.08158692 | 0.0001393 | 0.00026784 |
| AL355472.4 | 0.08069953 | 0.257591068 | 1.6744504 | 0.00945424 | 0.01352058 |
| AC138466.1 | 0.044216946 | 0.16486181 | 1.89858595 | 0.0135203 | 0.01888479 |
| AC245123.1 | 0.013332358 | 0.005067852 | -1.395485532 | 5.59E-15 | 5.12E-14 |
| LINC02285 | 0.198984428 | 0.09644207 | -1.044921009 | 8.28E-08 | 2.60E-07 |
| AC018766.1 | 0.094925131 | 0.389267373 | 2.035899433 | 2.13E-08 | 7.31E-08 |
| AL391832.3 | 0.053033296 | 0.113042595 | 1.091896175 | 0.01036548 | 0.01471952 |
| AC068189.1 | 0.491754379 | 0.095237148 | -2.368341576 | 5.76E-16 | 6.32E-15 |
| AC068722.2 | 0.702897627 | 0.254309087 | -1.466731568 | 1.93E-12 | 1.21E-11 |
| LINC01985 | 0.296109349 | 0.029477569 | -3.328440598 | 1.99E-32 | 6.78E-30 |
| LINC01055 | 0.245240095 | 0.050772038 | -2.272088807 | 1.61E-16 | 1.93E-15 |
| AL445490.1 | 0.054408446 | 0.142212049 | 1.386141163 | 0.0091104 | 0.01307342 |
| AC124319.2 | 0.136791219 | 0.297892079 | 1.12281414 | 0.0074094 | 0.01080595 |
| AC083805.1 | 0.022232282 | 0.049211008 | 1.146325042 | 0.01342313 | 0.01875386 |
| AC022144.1 | 0.14643793 | 0.729083583 | 2.315794928 | 5.69E-14 | 4.47E-13 |
| AL512306.2 | 0.280439739 | 0.139681691 | -1.005547866 | 5.10E-12 | 3.02E-11 |
| AC007938.2 | 0.022268998 | 0.100471172 | 2.173673072 | 1.64E-08 | 5.72E-08 |
| AL691482.3 | 1.632393327 | 5.142922618 | 1.655599728 | 9.38E-12 | 5.33E-11 |
| AC244205.1 | 0.358430037 | 0.115231196 | -1.637160194 | 1.94E-13 | 1.42E-12 |
| AC036176.3 | 0.006805679 | 0.307069933 | 5.495684341 | 2.81E-08 | 9.44E-08 |
| LINC00337 | 0.089153151 | 0.185323885 | 1.055691139 | 3.72E-06 | 9.21E-06 |
| AL645608.1 | 0.038261325 | 0.122282664 | 1.676261164 | 0.00056817 | 0.00100116 |
| AC002398.2 | 0.464968272 | 0.042540942 | -3.450208396 | 1.65E-17 | 2.33E-16 |
| AC092143.3 | 0.070072277 | 0.257646778 | 1.878478865 | 6.22E-09 | 2.32E-08 |
| AL008727.1 | 0.011314583 | 0.03574776 | 1.65966949 | 6.23E-05 | 0.0001269 |
| FOXP4-AS1 | 0.499237377 | 2.823778232 | 2.499828933 | 4.31E-22 | 1.73E-20 |
| KLF7-IT1 | 0.060076177 | 0.438490142 | 2.867679495 | 0.00211278 | 0.00338753 |
| AL137002.1 | 0.157303246 | 0.457622259 | 1.540608783 | 0.00026394 | 0.00048739 |
| AC012676.4 | 0.08613065 | 0.241567385 | 1.487827056 | 2.24E-05 | 4.92E-05 |
| AC004477.1 | 0.073567048 | 0.18396022 | 1.322262213 | 5.23E-08 | 1.69E-07 |
| AP000695.1 | 0.073680943 | 0.311641793 | 2.08052529 | 1.24E-10 | 5.99E-10 |
| AC006064.3 | 0.096945433 | 0.220215874 | 1.183673629 | 0.00067537 | 0.00117473 |
| AC026150.1 | 0.026782278 | 0.012237854 | -1.129928107 | 3.01E-06 | 7.58E-06 |
| AL139351.1 | 0.019712417 | 0.10082071 | 2.354615459 | 7.04E-05 | 0.00014166 |
| AP006545.2 | 0.110216164 | 0.226466849 | 1.038964058 | 0.02624334 | 0.03470893 |
| AC239803.3 | 0.045591656 | 0.162032382 | 1.829440453 | 9.45E-06 | 2.19E-05 |
| LINC01564 | 0.061311468 | 0.284264348 | 2.213004324 | 0.00014008 | 0.00026906 |
| SLC7A11-AS1 | 0.008132829 | 0.146897751 | 4.174911182 | 7.37E-09 | 2.71E-08 |
| AL161719.1 | 0.513007171 | 0.213256962 | -1.266386154 | 8.21E-15 | 7.37E-14 |
| GAS6-AS1 | 0.210021594 | 2.529554432 | 3.59027371 | 2.14E-19 | 4.37E-18 |
| C15orf54 | 0.003159495 | 0.071454799 | 4.499265031 | 1.76E-07 | 5.30E-07 |
| LEF1-AS1 | 0.012908213 | 0.07302696 | 2.500139941 | 6.56E-18 | 1.00E-16 |
| Z97192.1 | 0.005973987 | 0.056539128 | 3.24248358 | 0.00446812 | 0.00678492 |
| AP004609.1 | 0.081923703 | 0.020176452 | -2.021608452 | 5.74E-10 | 2.50E-09 |
| AC079209.1 | 0.187283836 | 0.092076939 | -1.024314609 | 2.04E-12 | 1.28E-11 |
| AC113361.1 | 0.03465498 | 0.113815801 | 1.715566256 | 0.00774957 | 0.01128395 |
| AL136988.2 | 0.080162225 | 0.290269137 | 1.856396723 | 0.00384395 | 0.00590117 |
| AP001453.2 | 0.75441089 | 3.241697104 | 2.103326889 | 3.29E-21 | 1.06E-19 |
| AC130371.2 | 1.812508625 | 0.486109377 | -1.89863499 | 2.03E-21 | 6.80E-20 |
| AP000812.1 | 0.087905649 | 0.037058701 | -1.246143569 | 4.45E-06 | 1.09E-05 |
| AL356417.2 | 0.06143484 | 0.209705761 | 1.771237544 | 2.72E-05 | 5.84E-05 |
| HMMR-AS1 | 0.010513253 | 0.043401464 | 2.045534603 | 0.00323083 | 0.00502215 |
| AL121583.1 | 0.226584197 | 0.490866575 | 1.11528369 | 0.0001136 | 0.00022139 |
| AC136475.9 | 0.070612987 | 0.379862153 | 2.427470535 | 0.00010213 | 0.00020076 |
| AC084125.2 | 0.192996196 | 0.434845833 | 1.171931599 | 2.18E-08 | 7.47E-08 |
| LINC02156 | 0.021751253 | 0.17723864 | 3.026522715 | 3.24E-07 | 9.38E-07 |
| AC090192.2 | 0.121730717 | 0.259827597 | 1.093861418 | 0.03697513 | 0.04773328 |
| AC096733.2 | 0.486156316 | 0.173564281 | -1.485950185 | 2.12E-17 | 2.95E-16 |
| AC018410.2 | 0.103947547 | 0.39736319 | 1.934602519 | 0.00158483 | 0.00260147 |
| AL133370.1 | 15.57638095 | 7.551256511 | -1.044571443 | 6.39E-09 | 2.38E-08 |
| MPPED2-AS1 | 0.064631423 | 0.011924378 | -2.438321681 | 6.68E-13 | 4.56E-12 |
| AL109804.1 | 0.062641799 | 0.183484004 | 1.550456736 | 4.96E-05 | 0.0001025 |
| LINC01285 | 0.023354844 | 0.074480447 | 1.673139952 | 1.11E-05 | 2.54E-05 |
| LINC01781 | 0.58491121 | 0.137419488 | -2.089631022 | 9.33E-17 | 1.17E-15 |
| TM4SF19-AS1 | 0.061192871 | 0.138334627 | 1.176726825 | 7.79E-07 | 2.13E-06 |
| AL110292.1 | 0.040637177 | 0.016422151 | -1.307157072 | 8.55E-19 | 1.50E-17 |
| AP000553.2 | 0.042459804 | 0.32708641 | 2.945502209 | 3.89E-11 | 2.02E-10 |
| AC073288.2 | 0.041938597 | 0.110844318 | 1.402184295 | 0.00119809 | 0.00199669 |
| AC079834.2 | 0.214700065 | 0.077629814 | -1.467639882 | 5.86E-16 | 6.42E-15 |
| AC011352.3 | 0.001809957 | 0.220995 | 6.931914768 | 2.52E-08 | 8.51E-08 |
| AC011944.1 | 0.039198466 | 0.089450143 | 1.190286603 | 0.00790713 | 0.01148271 |
| AL356310.1 | 0.033114724 | 0.13323725 | 2.00845275 | 9.34E-06 | 2.17E-05 |
| AL109615.3 | 0.033762375 | 0.905990757 | 4.74600804 | 1.31E-25 | 1.52E-23 |
| LINC02256 | 0.290724703 | 0.123283345 | -1.237675754 | 1.54E-12 | 9.85E-12 |
| AC010336.2 | 0.265886097 | 0.073034298 | -1.864162311 | 2.20E-16 | 2.59E-15 |
| LINC01954 | 1.131258328 | 0.162266387 | -2.801492329 | 2.55E-23 | 1.46E-21 |
| AC007991.4 | 0.028473074 | 0.184148464 | 2.693199188 | 0.00020524 | 0.000385 |
| AL158152.2 | 0.433436639 | 0.992092611 | 1.194653691 | 2.19E-07 | 6.48E-07 |
| ASAP1-IT2 | 0.01509229 | 0.132663197 | 3.135884531 | 0.00825962 | 0.01193737 |
| AC018697.1 | 0.081937639 | 0.01963992 | -2.060737259 | 6.72E-17 | 8.66E-16 |
| SNHG16 | 2.101056117 | 6.051035477 | 1.526067348 | 7.34E-25 | 6.57E-23 |
| AL162412.1 | 0.078114147 | 0.036270578 | -1.106784101 | 1.43E-10 | 6.86E-10 |
| LINC00920 | 0.603793272 | 2.457995728 | 2.025355823 | 1.02E-10 | 4.97E-10 |
| SPRY4-AS1 | 0.023688147 | 0.246570251 | 3.379761518 | 5.27E-23 | 2.69E-21 |
| AC073333.1 | 0.13043367 | 0.524276933 | 2.007012736 | 3.42E-11 | 1.79E-10 |
| AC007216.4 | 0.170604843 | 0.421335419 | 1.304310597 | 8.73E-05 | 0.00017345 |
| KCNIP2-AS1 | 0.141876679 | 0.339073325 | 1.256959825 | 2.97E-11 | 1.56E-10 |
| MKX-AS1 | 0.050043495 | 0.015958829 | -1.648827806 | 3.53E-08 | 1.17E-07 |
| AL162171.1 | 0.819782306 | 0.326026713 | -1.330250674 | 2.06E-15 | 2.05E-14 |
| LINC00996 | 0.994852418 | 0.358287979 | -1.473362885 | 1.83E-15 | 1.84E-14 |
| AC098864.1 | 0.012058403 | 0.064385239 | 2.416691069 | 1.12E-05 | 2.56E-05 |
| DSG1-AS1 | 0.013186961 | 0.097670688 | 2.888813496 | 2.14E-07 | 6.33E-07 |
| AL391244.3 | 0.693113198 | 1.820372771 | 1.393071017 | 1.63E-18 | 2.77E-17 |
| AC020663.3 | 0.075601721 | 0.276439273 | 1.870471611 | 1.13E-06 | 3.03E-06 |
| LINC02012 | 1.487818572 | 0.659983882 | -1.172695916 | 1.88E-10 | 8.84E-10 |
| AC082651.3 | 0.042453845 | 0.104110372 | 1.294146694 | 0.00906158 | 0.01301364 |
| AC005324.5 | 0.063550051 | 0.141683037 | 1.156701851 | 0.0006504 | 0.0011342 |
| CFAP44-AS1 | 0.00969594 | 0.111450626 | 3.522880192 | 0.00385606 | 0.00591479 |
| CLMAT3 | 0.011202145 | 0.190835931 | 4.090485963 | 3.12E-08 | 1.04E-07 |
| AC073111.1 | 0.131906126 | 0.409905918 | 1.635781251 | 6.12E-06 | 1.46E-05 |
| ELFN1-AS1 | 0.283364139 | 9.465950154 | 5.062018228 | 3.70E-24 | 2.65E-22 |
| LINC01394 | 0.239117022 | 0.068194459 | -1.809990399 | 1.58E-15 | 1.60E-14 |
| AL118511.1 | 0.037828272 | 0.154579892 | 2.030815886 | 1.48E-10 | 7.05E-10 |
| LINC00265 | 1.019268432 | 2.198457378 | 1.108957517 | 2.50E-10 | 1.15E-09 |
| AC007750.1 | 0.00663318 | 0.084592251 | 3.672752941 | 3.73E-12 | 2.26E-11 |
| AC025857.2 | 1.269421689 | 4.860813913 | 1.937026507 | 4.74E-19 | 9.01E-18 |
| AC021321.1 | 0.116578473 | 0.239749481 | 1.040226288 | 0.0010168 | 0.00171553 |
| AC137767.1 | 0.231790198 | 0.585743956 | 1.337450608 | 1.00E-13 | 7.59E-13 |
| AP000866.2 | 1.962505422 | 0.547254848 | -1.842411905 | 4.69E-22 | 1.85E-20 |
| AP000350.7 | 0.006856661 | 0.024274348 | 1.823854456 | 9.38E-08 | 2.93E-07 |
| LINC01558 | 0.576888283 | 1.560622012 | 1.435757288 | 1.24E-05 | 2.83E-05 |
| PVT1 | 0.387063956 | 3.002800625 | 2.955664816 | 3.96E-26 | 5.40E-24 |
| LINC01124 | 0.258915568 | 1.55964617 | 2.590665148 | 4.11E-15 | 3.86E-14 |
| LINC02031 | 0.171255675 | 0.025424874 | -2.751839247 | 1.68E-24 | 1.41E-22 |
| LINC02525 | 0.008853351 | 0.120787716 | 3.770106302 | 0.00497058 | 0.007473 |
| LINC01641 | 0.005998689 | 0.044372651 | 2.886951622 | 0.01201291 | 0.01691798 |
| SLC12A5-AS1 | 0.027492076 | 0.141446918 | 2.363172985 | 2.38E-09 | 9.42E-09 |
| AP000679.1 | 0.013972903 | 0.038975388 | 1.479931578 | 0.00797327 | 0.01155722 |
| LINC02244 | 0.043631911 | 0.357165187 | 3.033135898 | 5.30E-08 | 1.71E-07 |
| LINC01140 | 0.175189432 | 0.055017759 | -1.670946456 | 3.13E-20 | 7.97E-19 |
| AC099329.2 | 0.056863858 | 0.027165983 | -1.06571074 | 8.51E-14 | 6.54E-13 |
| AC004076.2 | 0.098967027 | 0.267872474 | 1.436526499 | 1.18E-09 | 4.84E-09 |
| AC010998.3 | 1.37798224 | 0.427539315 | -1.688428299 | 2.35E-17 | 3.23E-16 |
| AP003032.1 | 0.132813695 | 0.493273483 | 1.892983812 | 1.88E-08 | 6.47E-08 |
| AC092718.2 | 0.064460985 | 0.184611092 | 1.517991089 | 2.09E-13 | 1.51E-12 |
| AC106820.3 | 0.221844491 | 0.590993593 | 1.413593763 | 9.33E-09 | 3.37E-08 |
| AC108463.3 | 0.017468199 | 0.15698164 | 3.167793051 | 1.67E-08 | 5.82E-08 |
| LINC01424 | 0.068592486 | 0.213389238 | 1.637364978 | 4.73E-10 | 2.08E-09 |
| AC097478.1 | 0.003946165 | 0.425365985 | 6.752109739 | 3.70E-07 | 1.06E-06 |
| AP005230.1 | 0.007427761 | 0.143379094 | 4.270763512 | 1.91E-15 | 1.91E-14 |
| LINC01748 | 0.076141913 | 1.333741441 | 4.13064439 | 2.53E-14 | 2.09E-13 |
| LINC01315 | 1.030850969 | 4.118504891 | 1.998284926 | 8.42E-17 | 1.07E-15 |
| LINC00402 | 0.118865774 | 0.03696831 | -1.684972356 | 2.59E-18 | 4.25E-17 |
| LINC01126 | 0.122566824 | 0.253801781 | 1.050133662 | 0.00048475 | 0.00086299 |
| LINC00974 | 0.686640287 | 0.027912121 | -4.620590834 | 9.88E-30 | 2.34E-27 |
| LINC00471 | 0.121776881 | 0.294656474 | 1.2747937 | 1.16E-07 | 3.57E-07 |
| CASC16 | 0.254684211 | 0.097550086 | -1.38449448 | 3.29E-13 | 2.33E-12 |
| AC073283.2 | 1.370169333 | 0.101999106 | -3.747725794 | 1.66E-26 | 2.51E-24 |
| NCAM1-AS1 | 0.050497712 | 0.015955123 | -1.662198291 | 6.12E-13 | 4.19E-12 |
| MIR3945HG | 0.043588939 | 0.169266119 | 1.957259245 | 0.00517813 | 0.00776578 |
| AL391056.1 | 0.124325296 | 1.203677319 | 3.275256917 | 5.08E-19 | 9.55E-18 |
| AC040169.3 | 0.189817467 | 0.504656237 | 1.410688227 | 1.00E-07 | 3.12E-07 |
| DEPDC1-AS1 | 0.027958648 | 0.069620292 | 1.316213267 | 0.00058699 | 0.00103021 |
| AC002546.1 | 0.172169133 | 0.065664437 | -1.39064237 | 8.72E-19 | 1.52E-17 |
| AL360181.1 | 0.185746108 | 0.520401652 | 1.486293563 | 0.0025626 | 0.00405392 |
| AL391244.2 | 0.2014301 | 0.087271332 | -1.206699559 | 4.90E-08 | 1.59E-07 |
| AL163952.1 | 0.021695968 | 0.062334623 | 1.522606773 | 0.0016698 | 0.00273271 |
| AL109976.1 | 0.018057997 | 0.075001356 | 2.054278848 | 8.66E-09 | 3.15E-08 |
| AC133528.1 | 0.068824714 | 0.26846758 | 1.963749263 | 2.11E-10 | 9.78E-10 |
| AC007038.1 | 0.112096962 | 0.414516495 | 1.886682336 | 1.39E-06 | 3.67E-06 |
| AC009414.2 | 3.329465424 | 1.526510467 | -1.125053076 | 3.17E-20 | 8.04E-19 |
| LINC02261 | 0.02613368 | 0.007858059 | -1.733665452 | 1.30E-21 | 4.47E-20 |
| AC003991.1 | 0.213962151 | 0.102466156 | -1.062208145 | 1.02E-13 | 7.73E-13 |
| AL365226.1 | 0.024228395 | 0.093243256 | 1.944300543 | 0.00062025 | 0.00108649 |
| AL365226.2 | 2.44482857 | 35.34067247 | 3.853524279 | 3.69E-05 | 7.79E-05 |
| AF127936.2 | 0.087945555 | 0.043510145 | -1.015258818 | 1.67E-11 | 9.11E-11 |
| AC080129.2 | 0.420682897 | 1.15274272 | 1.454265483 | 1.62E-07 | 4.89E-07 |
| AL132642.1 | 0.891597505 | 0.134907881 | -2.724417949 | 4.45E-24 | 3.11E-22 |
| LINC00640 | 0.058294384 | 0.020870275 | -1.48190731 | 1.84E-14 | 1.55E-13 |
| AC107308.1 | 0.027607475 | 0.299890031 | 3.441302735 | 1.84E-06 | 4.77E-06 |
| AC090092.1 | 0.014651632 | 0.048654039 | 1.731498259 | 0.00384532 | 0.00590117 |
| AC011498.6 | 0.187924019 | 0.486050505 | 1.370956759 | 1.74E-07 | 5.25E-07 |
| AC253536.6 | 0.421981214 | 0.866079386 | 1.037320498 | 7.13E-07 | 1.97E-06 |
| AC109309.1 | 0.212271864 | 0.042744972 | -2.312086519 | 1.73E-20 | 4.62E-19 |
| AC005387.2 | 0.126751137 | 0.325656765 | 1.36135351 | 2.21E-07 | 6.53E-07 |
| AC005041.3 | 0.32663892 | 0.765614367 | 1.2289212 | 9.63E-09 | 3.47E-08 |
| LURAP1L-AS1 | 0.032200825 | 0.177209129 | 2.46028339 | 0.00021915 | 0.00040967 |
| AC008440.3 | 0.049256232 | 0.265046518 | 2.427867405 | 1.23E-05 | 2.81E-05 |
| AC008403.3 | 0.018073199 | 0.198417375 | 3.456614599 | 1.56E-14 | 1.34E-13 |
| AC020907.1 | 0.017043885 | 0.211348636 | 3.632298639 | 0.00014695 | 0.00028176 |
| AC010776.2 | 0.422828047 | 0.029533352 | -3.839654048 | 1.74E-25 | 1.94E-23 |
| FSIP2-AS1 | 0.098321388 | 0.381063629 | 1.954454731 | 3.21E-06 | 8.04E-06 |
| AC015660.2 | 0.101741136 | 0.454047617 | 2.157940492 | 0.00011057 | 0.00021595 |
| AC073957.3 | 0.719799897 | 1.489485501 | 1.049146279 | 1.88E-05 | 4.18E-05 |
| LINC01016 | 0.061365969 | 0.018402029 | -1.737573978 | 4.66E-18 | 7.30E-17 |
| Z99289.2 | 0.00514838 | 0.113357556 | 4.460618251 | 2.82E-06 | 7.13E-06 |
| AC008105.1 | 0.09957143 | 0.244366154 | 1.295240724 | 2.00E-07 | 5.97E-07 |
| AC007622.2 | 0.158089133 | 0.369747517 | 1.225802256 | 0.00555 | 0.00828928 |
| INO80-AS1 | 0.025432447 | 0.122873229 | 2.272428464 | 0.00440743 | 0.00669649 |
| AL008723.1 | 0.045877194 | 0.346453613 | 2.916813148 | 7.23E-06 | 1.71E-05 |
| LINC02511 | 0.021577286 | 0.006655601 | -1.696872549 | 8.50E-19 | 1.50E-17 |
| AP003031.1 | 0.011404929 | 0.096642795 | 3.083004677 | 3.85E-09 | 1.47E-08 |
| AC023154.1 | 0.004319643 | 0.041966275 | 3.280246397 | 0.0034261 | 0.00529699 |
| AC005358.2 | 0.283729322 | 0.019589726 | -3.856346115 | 2.55E-24 | 1.99E-22 |
| AL023803.2 | 0.304839866 | 0.796792428 | 1.386152351 | 1.57E-12 | 1.01E-11 |
| AL161729.2 | 0.039448233 | 0.299997299 | 2.92691694 | 0.0004006 | 0.0007231 |
| AC254629.1 | 8.38899045 | 3.972507271 | -1.078447344 | 3.29E-12 | 2.01E-11 |
| AC010834.3 | 0.467155979 | 1.177721681 | 1.334022402 | 4.99E-06 | 1.21E-05 |
| AC064807.2 | 0.046742985 | 0.670353782 | 3.842100923 | 1.24E-13 | 9.28E-13 |
| UBE2Q1-AS1 | 0.162493274 | 0.431690016 | 1.409615725 | 3.83E-07 | 1.10E-06 |
| AL442067.2 | 0.072142662 | 0.430563533 | 2.57730158 | 2.54E-05 | 5.50E-05 |
| AC112722.1 | 0.090431096 | 0.200754962 | 1.150544783 | 0.00480651 | 0.00725031 |
| AC092910.3 | 0.26569607 | 0.57451679 | 1.112572169 | 1.62E-09 | 6.52E-09 |
| AC011405.1 | 0.018150664 | 0.164174804 | 3.17713847 | 1.84E-05 | 4.08E-05 |
| SLC5A4-AS1 | 0.020166181 | 0.151302961 | 2.907430396 | 1.43E-06 | 3.77E-06 |
| AC010735.2 | 0.130242371 | 0.28416715 | 1.125540913 | 0.0025401 | 0.00402182 |
| AC083900.1 | 1.120205675 | 0.392352855 | -1.513540039 | 1.24E-14 | 1.07E-13 |
| AC020779.2 | 1.242040043 | 0.165042822 | -2.911799385 | 1.89E-21 | 6.35E-20 |
| LINC01871 | 5.778569758 | 2.631195395 | -1.13499407 | 6.73E-11 | 3.37E-10 |
| EPB41L4A-DT | 0.620820269 | 0.236192808 | -1.394210625 | 1.08E-17 | 1.59E-16 |
| SNAP47-AS1 | 0.0041174 | 0.036345009 | 3.141951841 | 5.12E-05 | 0.00010563 |
| AC026356.2 | 0.070837902 | 0.309589649 | 2.127763842 | 2.30E-14 | 1.91E-13 |
| AC021218.1 | 1.481695417 | 8.857034387 | 2.579574808 | 7.37E-20 | 1.67E-18 |
| BX255923.1 | 0.060603713 | 0.019808061 | -1.613318544 | 3.32E-08 | 1.10E-07 |
| AP003352.1 | 0.829257378 | 2.413769443 | 1.541396032 | 8.48E-19 | 1.50E-17 |
| AC108718.1 | 0.081275835 | 0.026498171 | -1.616933709 | 5.40E-11 | 2.74E-10 |
| AC125603.2 | 0.075207039 | 0.793453608 | 3.399206274 | 2.94E-05 | 6.29E-05 |
| AC108751.4 | 0.020347177 | 0.131130228 | 2.688099764 | 1.65E-09 | 6.65E-09 |
| MYO16-AS1 | 0.005699319 | 0.197041537 | 5.111566319 | 1.12E-06 | 2.99E-06 |
| RUSC1-AS1 | 0.815245735 | 2.304706092 | 1.499275888 | 7.43E-14 | 5.79E-13 |
| LINC01429 | 0.002354929 | 0.060358163 | 4.679793294 | 1.18E-06 | 3.15E-06 |
| LINC00618 | 0.051475586 | 0.326232151 | 2.663938729 | 6.65E-12 | 3.86E-11 |
| LINC02351 | 0.089646826 | 0.036200077 | -1.308259731 | 1.85E-06 | 4.78E-06 |
| LUCAT1 | 0.022093948 | 0.399257437 | 4.175596165 | 2.72E-15 | 2.66E-14 |
| AC010168.1 | 0.061728679 | 0.259206218 | 2.070087504 | 0.0025982 | 0.00410785 |
| AL118508.2 | 0.030100616 | 0.012682134 | -1.246995524 | 3.19E-08 | 1.06E-07 |
| Z95331.1 | 0.015993122 | 0.076987103 | 2.26716517 | 1.53E-10 | 7.28E-10 |
| AC020661.3 | 0.020540056 | 0.253384153 | 3.624814311 | 0.00196529 | 0.00316782 |
| LINC01778 | 0.287087989 | 0.096251274 | -1.576615431 | 0.00013242 | 0.00025543 |
| AC131009.3 | 1.156831951 | 3.634835273 | 1.65171068 | 4.13E-19 | 7.92E-18 |
| AC093249.2 | 0.19919237 | 0.402188001 | 1.013707655 | 2.50E-05 | 5.41E-05 |
| AL031666.2 | 0.053584943 | 0.200786862 | 1.905765287 | 0.00095495 | 0.00161819 |
| LACTB2-AS1 | 0.025619053 | 0.095759292 | 1.902195357 | 5.55E-07 | 1.56E-06 |
| AL359922.2 | 0.050217778 | 0.454922385 | 3.179350332 | 3.20E-15 | 3.07E-14 |
| AL138689.1 | 0.304048911 | 1.221805149 | 2.0066389 | 2.54E-06 | 6.47E-06 |
| AC053503.4 | 3.351621369 | 1.430300824 | -1.228540568 | 7.66E-10 | 3.26E-09 |
| AP002409.1 | 0.011071894 | 0.002365107 | -2.226924496 | 5.29E-21 | 1.63E-19 |
| AC105942.1 | 5.829246804 | 2.819702348 | -1.047766608 | 1.06E-15 | 1.11E-14 |
| AC090164.3 | 0.078492551 | 0.02959279 | -1.407310015 | 3.32E-14 | 2.70E-13 |
| AL133215.2 | 0.542482582 | 1.248158023 | 1.202151875 | 1.52E-12 | 9.78E-12 |
| AC132938.2 | 0.054505789 | 0.124236613 | 1.18860903 | 0.00891833 | 0.01281804 |
| AC084346.2 | 0.01175648 | 0.085060842 | 2.855038954 | 9.70E-10 | 4.07E-09 |
| SNHG6 | 22.30177387 | 61.43249115 | 1.461843421 | 6.96E-22 | 2.60E-20 |
| C3orf35 | 0.106450321 | 0.266325959 | 1.323012757 | 1.92E-09 | 7.65E-09 |
| AP001619.1 | 0.065750158 | 0.223843198 | 1.76742221 | 2.38E-05 | 5.20E-05 |
| PURPL | 0.016851763 | 0.525475868 | 4.962653072 | 8.50E-06 | 1.98E-05 |
| AL136418.1 | 0.116106148 | 0.677082308 | 2.543886859 | 0.00016743 | 0.00031812 |
| AC011700.1 | 0.028619688 | 0.159455664 | 2.478075475 | 1.97E-12 | 1.24E-11 |
| AC092376.2 | 0.157770917 | 0.077771439 | -1.020518947 | 1.70E-11 | 9.26E-11 |
| AL158063.1 | 0.10983452 | 0.407175534 | 1.89031932 | 6.86E-07 | 1.90E-06 |
| PKP4-AS1 | 0.021193232 | 0.113674837 | 2.423237425 | 1.21E-10 | 5.85E-10 |
| PROSER2-AS1 | 0.029266066 | 0.063690586 | 1.121851305 | 0.01028581 | 0.0146178 |
| DIO3OS | 1.430920772 | 2.903983302 | 1.021089363 | 0.03619623 | 0.04680532 |
| AC008808.2 | 0.190260879 | 0.041636875 | -2.192045249 | 7.73E-15 | 6.98E-14 |
| LINC02362 | 1.450980143 | 0.49892403 | -1.540135714 | 4.14E-13 | 2.91E-12 |
| MCM3AP-AS1 | 0.326483344 | 0.673430551 | 1.044519781 | 2.11E-13 | 1.52E-12 |
| C17orf77 | 0.062471777 | 1.022661797 | 4.032980743 | 1.20E-06 | 3.19E-06 |
| AC123023.1 | 0.022421425 | 1.986487915 | 6.469198266 | 5.20E-20 | 1.24E-18 |
| AP001107.6 | 0.098180734 | 0.221429785 | 1.173337437 | 1.58E-05 | 3.55E-05 |
| LINC01433 | 0.011286272 | 0.148428502 | 3.717127288 | 4.36E-15 | 4.09E-14 |
| AL390961.2 | 0.024133332 | 0.139030053 | 2.526297774 | 0.00851169 | 0.01226916 |
| LINC00488 | 0.10711291 | 0.023318647 | -2.199576396 | 5.59E-13 | 3.87E-12 |
| AC092718.4 | 2.827330398 | 7.893462836 | 1.48121786 | 1.13E-20 | 3.19E-19 |
| AC010997.3 | 0.10105918 | 0.22322576 | 1.14330314 | 8.98E-05 | 0.00017778 |
| AC010463.3 | 0.19172037 | 0.446514728 | 1.219704134 | 2.99E-07 | 8.69E-07 |
| DLGAP1-AS5 | 0.006985026 | 0.420529418 | 5.911797447 | 0.01018288 | 0.01448937 |
| AC106739.1 | 1.025641781 | 0.496593861 | -1.046388607 | 3.43E-14 | 2.78E-13 |
| AC145207.1 | 0.011891625 | 0.162845027 | 3.775481872 | 1.28E-10 | 6.16E-10 |
| C15orf56 | 0.050457073 | 0.127490986 | 1.337266819 | 1.98E-07 | 5.91E-07 |
| AL442125.2 | 0.192763646 | 0.555285757 | 1.526397399 | 0.00270451 | 0.0042611 |
| AL133383.1 | 0.42821086 | 0.128003859 | -1.742134081 | 1.73E-12 | 1.10E-11 |
| AC092919.1 | 0.050685571 | 0.192588854 | 1.925877206 | 0.01444553 | 0.02006406 |
| AC134312.4 | 0.102700952 | 0.041829071 | -1.295871686 | 1.52E-14 | 1.30E-13 |
| ZNF582-AS1 | 0.44592466 | 0.14704558 | -1.600536568 | 6.80E-19 | 1.24E-17 |
| LINC01836 | 0.101695138 | 0.701888625 | 2.786991418 | 5.79E-18 | 8.90E-17 |
| AL031667.3 | 0.07624512 | 0.377492202 | 2.307729929 | 7.84E-09 | 2.88E-08 |
| PWRN1 | 0.024451955 | 0.01033818 | -1.241967498 | 6.65E-18 | 1.01E-16 |
| LINC01910 | 0.009623465 | 0.034017069 | 1.821630503 | 0.00070965 | 0.00123161 |
| PCAT14 | 0.008214939 | 0.558520817 | 6.087217365 | 6.36E-05 | 0.0001292 |
| AL359711.2 | 0.989900346 | 0.345678661 | -1.517851747 | 2.19E-14 | 1.82E-13 |
| LINC00630 | 0.208361252 | 0.500659745 | 1.264743455 | 2.87E-13 | 2.04E-12 |
| AL035420.3 | 0.071339301 | 0.218864378 | 1.617268182 | 0.00011382 | 0.00022175 |
| AL161785.1 | 0.772412612 | 0.299721509 | -1.365749099 | 6.32E-15 | 5.75E-14 |
| MCCC1-AS1 | 0.195725072 | 0.504682971 | 1.366548834 | 1.39E-07 | 4.23E-07 |
| LINC01783 | 0.087113009 | 0.023884242 | -1.866829063 | 1.08E-11 | 6.12E-11 |
| AL390860.1 | 0.044681009 | 0.01185339 | -1.91436211 | 9.98E-20 | 2.20E-18 |
| AF064860.2 | 0.19469543 | 0.046707955 | -2.059478823 | 2.10E-13 | 1.51E-12 |
| LINC02268 | 0.148520313 | 0.013822427 | -3.425577421 | 4.70E-23 | 2.44E-21 |
| AL359232.1 | 0.079025183 | 0.301590477 | 1.932206492 | 2.06E-18 | 3.43E-17 |
| GABPB1-AS1 | 0.524732031 | 1.243380659 | 1.24461528 | 3.85E-07 | 1.10E-06 |
| LINC01301 | 0.090070756 | 0.338233079 | 1.908887083 | 1.86E-08 | 6.44E-08 |
| TLX1NB | 0.013724013 | 0.061040205 | 2.153057422 | 9.52E-05 | 0.00018803 |
| AC013452.2 | 0.030982052 | 0.094778223 | 1.613122937 | 5.69E-05 | 0.00011652 |
| AC004080.5 | 0.21948189 | 0.098279662 | -1.159137105 | 2.04E-07 | 6.08E-07 |
| AL355312.2 | 0.103604527 | 0.285500235 | 1.46240489 | 4.76E-07 | 1.35E-06 |
| LINC00092 | 0.418047269 | 0.076408993 | -2.451851723 | 3.12E-24 | 2.36E-22 |
| LINC01169 | 0.006267295 | 0.544923464 | 6.442066838 | 2.59E-10 | 1.19E-09 |
| YEATS2-AS1 | 0.114253386 | 0.319231305 | 1.48236521 | 3.52E-11 | 1.84E-10 |
| LINC01798 | 0.133017011 | 0.046909461 | -1.503659919 | 8.03E-13 | 5.43E-12 |
| AC079684.1 | 0.28336659 | 1.00058909 | 1.820108053 | 2.46E-11 | 1.31E-10 |
| AC003965.2 | 0.118237279 | 1.29757054 | 3.456056089 | 5.53E-18 | 8.53E-17 |
| AC022165.1 | 0.031863795 | 0.134488977 | 2.077497906 | 0.00071561 | 0.00124117 |
| AC120042.1 | 0.024495615 | 0.071637896 | 1.548199455 | 0.03143926 | 0.04106307 |
| AP006621.2 | 0.651923641 | 1.538627443 | 1.238869047 | 5.05E-05 | 0.0001042 |
| HS1BP3-IT1 | 0.137283255 | 0.320677648 | 1.223968129 | 0.00024844 | 0.00046082 |
| AC012676.1 | 0.167584289 | 0.509783764 | 1.604998519 | 4.23E-17 | 5.62E-16 |
| AC114296.1 | 0.211726436 | 0.617268306 | 1.543696301 | 0.02397071 | 0.03199028 |
| IFNG-AS1 | 0.191933105 | 0.091579373 | -1.067508975 | 2.29E-08 | 7.80E-08 |
| AP000944.1 | 0.351282758 | 0.060066671 | -2.547996162 | 1.24E-16 | 1.52E-15 |
| AC100791.2 | 0.019027157 | 0.433801647 | 4.5109036 | 1.90E-16 | 2.26E-15 |
| AC112491.1 | 1.348253997 | 2.935302493 | 1.122416875 | 1.68E-05 | 3.76E-05 |
| TH2LCRR | 0.094861036 | 0.260933868 | 1.459796676 | 0.02640255 | 0.03490258 |
| AC127024.4 | 0.405270857 | 1.447973365 | 1.837076726 | 2.60E-08 | 8.78E-08 |
| AL008721.1 | 0.020698149 | 0.116174114 | 2.488715011 | 7.19E-05 | 0.00014449 |
| AP000253.1 | 0.025096247 | 0.051461706 | 1.036027634 | 0.01879428 | 0.02554467 |
| PIK3IP1-AS1 | 0.209620329 | 0.520682766 | 1.312626021 | 3.62E-09 | 1.40E-08 |
| AC073842.2 | 0.18095647 | 0.503926915 | 1.477571825 | 9.10E-10 | 3.84E-09 |
| AC004923.4 | 0.213038023 | 0.508323875 | 1.254637044 | 8.39E-10 | 3.55E-09 |
| LINC01273 | 0.155942887 | 0.519557968 | 1.736266974 | 5.88E-10 | 2.55E-09 |
| EVX1-AS | 0.016957574 | 0.483299457 | 4.832915704 | 7.10E-11 | 3.54E-10 |
| AC016738.2 | 0.008526933 | 0.070365591 | 3.044771287 | 0.00059181 | 0.001038 |
| NKAIN3-IT1 | 0.180674985 | 0.027757364 | -2.702454283 | 2.87E-20 | 7.41E-19 |
| Z98885.3 | 0.057354766 | 0.168649237 | 1.556040503 | 0.02804203 | 0.03697127 |
| FIRRE | 0.010701573 | 0.683801863 | 5.997683572 | 2.96E-20 | 7.62E-19 |
| ZNF790-AS1 | 0.368838975 | 0.180708596 | -1.029325975 | 1.74E-15 | 1.76E-14 |
| UBE2R2-AS1 | 0.176368024 | 0.629722994 | 1.836128332 | 2.73E-06 | 6.93E-06 |
| MIR181A2HG | 0.136287802 | 0.670286077 | 2.298120523 | 6.95E-09 | 2.57E-08 |
| AC007405.3 | 0.528042326 | 2.248989737 | 2.090551596 | 3.90E-15 | 3.68E-14 |
| AC123912.4 | 0.801703257 | 0.079754027 | -3.329439073 | 6.42E-21 | 1.93E-19 |
| LINC00922 | 0.008981864 | 0.065448942 | 2.865283023 | 2.87E-10 | 1.31E-09 |
| LINC00939 | 0.007574088 | 0.038117235 | 2.331299458 | 0.00652761 | 0.0096281 |
| AP002761.1 | 0.109231748 | 0.278158144 | 1.348513118 | 0.00275664 | 0.00433448 |
| AC093893.1 | 0.052014548 | 0.015110534 | -1.783360577 | 3.27E-18 | 5.22E-17 |
| LINC02036 | 0.008952084 | 0.046329649 | 2.371640203 | 0.00050846 | 0.00090108 |
| AC009065.5 | 7.188301615 | 16.10788324 | 1.164044071 | 5.52E-15 | 5.07E-14 |
| AC092718.1 | 0.01337704 | 0.059596058 | 2.155457937 | 0.00024854 | 0.00046082 |
| LINC02408 | 0.133916042 | 0.043207269 | -1.63198284 | 3.72E-15 | 3.53E-14 |
| STPG3-AS1 | 0.106814668 | 0.259420056 | 1.280180249 | 0.00015988 | 0.00030462 |
| AC002451.1 | 0.27244645 | 0.123535104 | -1.141051635 | 6.84E-12 | 3.96E-11 |
| AC025165.1 | 0.18008809 | 0.081725491 | -1.139844733 | 4.10E-08 | 1.35E-07 |
| AC108136.1 | 0.004615182 | 0.120173515 | 4.702587673 | 7.99E-07 | 2.19E-06 |
| FOXD3-AS1 | 0.419971686 | 0.085322475 | -2.29929434 | 1.05E-18 | 1.81E-17 |
| AC118758.3 | 0.001105732 | 0.003207021 | 1.536231504 | 0.02945135 | 0.03867972 |
| AC115102.1 | 0.049598005 | 0.112089111 | 1.176292132 | 0.0339373 | 0.04407242 |
| CSNK1G2-AS1 | 0.03372898 | 0.132441201 | 1.973291425 | 0.00033407 | 0.00061008 |
| AC079466.1 | 0.00089295 | 0.257581001 | 8.172230957 | 2.59E-10 | 1.19E-09 |
| PTGES2-AS1 | 0.049931674 | 0.462534689 | 3.211534393 | 2.40E-21 | 7.89E-20 |
| MIR137HG | 0.009047518 | 0.003846719 | -1.233893823 | 1.37E-17 | 1.98E-16 |
| AL161729.3 | 0.086694447 | 0.246949362 | 1.510203747 | 5.02E-05 | 0.00010365 |
| FMR1-AS1 | 0.009687602 | 0.040464678 | 2.062451566 | 1.46E-05 | 3.30E-05 |
| SH3PXD2A-AS1 | 0.280022452 | 2.346439515 | 3.066858861 | 3.38E-17 | 4.55E-16 |
| AC087521.2 | 0.036343326 | 0.223345831 | 2.619516964 | 0.00090817 | 0.00154517 |
| AC116914.1 | 0.071211752 | 0.18653339 | 1.38924664 | 0.00048833 | 0.00086852 |
| AC090152.1 | 2.196002766 | 0.923415999 | -1.249827237 | 4.78E-16 | 5.29E-15 |
| PGM5-AS1 | 4.039962097 | 0.185989334 | -4.441049966 | 8.34E-26 | 1.06E-23 |
| AC010336.5 | 0.076720649 | 0.01895359 | -2.017143808 | 6.16E-06 | 1.47E-05 |
| AL807752.4 | 0.01968859 | 0.128821739 | 2.709944379 | 2.72E-08 | 9.15E-08 |
| LINC02139 | 0.064841032 | 0.030603879 | -1.083192527 | 1.92E-11 | 1.04E-10 |
| SLC12A9-AS1 | 0.64315367 | 3.011727251 | 2.227355733 | 1.01E-19 | 2.21E-18 |
| AC048337.1 | 0.027707119 | 0.077120303 | 1.476853996 | 0.00019577 | 0.00036851 |
| AC073130.1 | 0.005146624 | 0.021120806 | 2.036966667 | 0.00549737 | 0.00821518 |
| AL157938.3 | 0.016120541 | 0.153552205 | 3.25175715 | 0.00234126 | 0.00372429 |
| DDN-AS1 | 0.058230067 | 0.555367585 | 3.2536068 | 2.65E-23 | 1.50E-21 |
| ITPK1-AS1 | 0.006820866 | 0.118569175 | 4.119630257 | 0.0343205 | 0.04452765 |
| LINC01891 | 0.052329936 | 0.023371069 | -1.162912778 | 7.58E-06 | 1.79E-05 |
| AC131953.1 | 0.061876953 | 0.203481323 | 1.717422328 | 0.00310918 | 0.00484826 |
| DENND5B-AS1 | 0.025075314 | 0.104004777 | 2.052310105 | 0.03380803 | 0.04392546 |
| LINC02163 | 0.002926396 | 1.325400866 | 8.823088212 | 3.09E-21 | 1.01E-19 |
| LINC01807 | 0.041047925 | 0.635565561 | 3.952659739 | 0.00074453 | 0.00128724 |
| AC078993.1 | 0.128733099 | 3.688647689 | 4.840637056 | 2.67E-05 | 5.75E-05 |
| LINC01012 | 0.128422952 | 0.333709267 | 1.377688688 | 2.53E-16 | 2.94E-15 |
| AC134312.5 | 0.065263454 | 0.287988342 | 2.141663173 | 1.35E-09 | 5.54E-09 |
| AL390719.2 | 5.757310166 | 14.80202695 | 1.362327906 | 1.04E-09 | 4.34E-09 |
| AC090409.1 | 0.039224214 | 0.114212757 | 1.541907357 | 9.93E-09 | 3.57E-08 |
| CLLU1 | 0.001198189 | 0.023895239 | 4.317796217 | 1.04E-05 | 2.39E-05 |
| AL353807.2 | 0.066996605 | 0.221025359 | 1.722051999 | 6.66E-05 | 0.00013467 |
| MIR34AHG | 0.058354531 | 0.247705829 | 2.085711243 | 2.34E-07 | 6.91E-07 |
| PCAT1 | 0.025277554 | 0.143161409 | 2.501713874 | 7.17E-16 | 7.75E-15 |
| AC093904.4 | 0.034227951 | 0.203318799 | 2.570496799 | 4.59E-11 | 2.35E-10 |
| FARSA-AS1 | 0.048314011 | 0.252686179 | 2.38683323 | 3.97E-05 | 8.35E-05 |
| AC104109.4 | 0.050065767 | 0.135998338 | 1.44169263 | 5.50E-06 | 1.33E-05 |
| AC078962.1 | 0.096238094 | 0.476508576 | 2.3078222 | 0.00461157 | 0.00698719 |
| LINC01869 | 0.071389607 | 0.173791263 | 1.283569584 | 8.61E-06 | 2.01E-05 |
| AC091806.1 | 0.017713579 | 0.064875435 | 1.872816529 | 1.01E-10 | 4.95E-10 |
| AL132765.2 | 0.063068971 | 0.157887046 | 1.32389051 | 0.0208493 | 0.02809272 |
| Z69666.1 | 0.036860307 | 0.182558236 | 2.30821678 | 1.64E-05 | 3.68E-05 |
| AJ239328.1 | 0.016746916 | 0.064530402 | 1.946083577 | 0.00295292 | 0.00462444 |
| LINC01982 | 0.141648245 | 0.036314369 | -1.963700306 | 5.09E-14 | 4.04E-13 |
| LINC01634 | 0.091926637 | 0.013640994 | -2.752534154 | 8.80E-18 | 1.32E-16 |
| LINC01694 | 0.031173058 | 0.255199236 | 3.033252431 | 3.13E-07 | 9.08E-07 |
| AC104024.2 | 0.004538316 | 0.111812614 | 4.622781941 | 6.86E-19 | 1.24E-17 |
| LINC01829 | 0.135077285 | 0.032954744 | -2.035227009 | 2.43E-05 | 5.29E-05 |
| DLG1-AS1 | 0.262644096 | 0.116491149 | -1.172888808 | 1.84E-14 | 1.55E-13 |
| AL591846.2 | 0.059176543 | 0.15405126 | 1.380313142 | 0.00132763 | 0.00219781 |
| AC008686.1 | 0.010657837 | 0.034045611 | 1.675554176 | 0.01509709 | 0.02088922 |
| AC007728.3 | 0.046566864 | 0.214588951 | 2.204200174 | 0.00016386 | 0.00031155 |
| MCF2L-AS1 | 2.470693045 | 7.291302721 | 1.561260817 | 2.09E-13 | 1.51E-12 |
| AC108206.1 | 0.037477927 | 0.015579155 | -1.266424195 | 5.64E-07 | 1.58E-06 |
| AC124067.4 | 3.331048344 | 9.70080099 | 1.542127583 | 2.88E-11 | 1.52E-10 |
| SEMA6A-AS2 | 0.465238839 | 0.084076438 | -2.468198087 | 2.73E-23 | 1.52E-21 |
| AC004466.2 | 0.046417938 | 0.170081871 | 1.873475034 | 0.02092955 | 0.02817997 |
| LRRC2-AS1 | 0.011379655 | 0.054417094 | 2.257603161 | 8.27E-09 | 3.01E-08 |
| AC016597.1 | 0.02353555 | 0.10776303 | 2.194948877 | 0.00057054 | 0.00100424 |
| DLGAP4-AS1 | 0.071620523 | 0.284822816 | 1.991619757 | 5.57E-11 | 2.82E-10 |
| AC004471.1 | 0.141843128 | 0.449115609 | 1.662790612 | 2.04E-12 | 1.28E-11 |
| AC007099.1 | 0.002087477 | 0.800627689 | 8.583227634 | 6.52E-22 | 2.49E-20 |
| AC010491.1 | 0.052238457 | 0.124792173 | 1.256343272 | 1.93E-09 | 7.69E-09 |
| AL353801.3 | 0.073192641 | 0.208787587 | 1.512265435 | 3.56E-05 | 7.53E-05 |
| AC021028.1 | 0.168797391 | 0.380480183 | 1.172528707 | 0.00212897 | 0.00341149 |
| AC019118.1 | 0.015472041 | 0.109484898 | 2.822996447 | 3.16E-05 | 6.73E-05 |
| AC021851.1 | 0.137140903 | 0.45227013 | 1.721525792 | 1.31E-13 | 9.70E-13 |
| FRMD6-AS2 | 0.234077843 | 0.03757846 | -2.639010552 | 1.29E-25 | 1.52E-23 |
| AL049836.1 | 0.201131804 | 2.283579817 | 3.505084086 | 4.05E-21 | 1.27E-19 |
| AC092168.2 | 0.038407785 | 0.821549944 | 4.418877603 | 5.24E-07 | 1.47E-06 |
| PLS3-AS1 | 0.027423779 | 0.099679923 | 1.861875568 | 3.00E-06 | 7.56E-06 |
| AC004988.1 | 0.009795667 | 0.194052573 | 4.308160089 | 1.05E-12 | 6.93E-12 |
| VAC14-AS1 | 0.014592059 | 0.169365263 | 3.536882632 | 6.02E-23 | 2.96E-21 |
| ACTN1-AS1 | 0.047340518 | 0.094758392 | 1.001178224 | 0.0001839 | 0.000347 |
| AP003086.2 | 0.025605211 | 0.18498144 | 2.852871194 | 1.94E-06 | 5.01E-06 |
| AC005993.1 | 0.310899703 | 0.885974 | 1.510815123 | 0.01152228 | 0.0162564 |
| LINC00346 | 0.151724864 | 0.698740933 | 2.203300129 | 6.57E-19 | 1.20E-17 |
| AL049840.1 | 0.920708793 | 1.879774694 | 1.029742923 | 0.0018177 | 0.00294734 |
| C1orf147 | 0.076907697 | 0.200545076 | 1.382726642 | 9.48E-07 | 2.56E-06 |
| FAM201A | 0.476872318 | 1.094666295 | 1.198816195 | 4.95E-11 | 2.52E-10 |
| RNF139-AS1 | 0.125952508 | 0.36529529 | 1.536183301 | 1.45E-13 | 1.07E-12 |
| AC022034.1 | 1.414076617 | 0.30775986 | -2.199983308 | 6.43E-17 | 8.33E-16 |
| AC087855.2 | 0.110376302 | 0.053939235 | -1.033023498 | 1.92E-12 | 1.21E-11 |
| AL139147.1 | 0.00689774 | 0.218593323 | 4.98598187 | 0.00022597 | 0.00042142 |
| ACBD3-AS1 | 0.330695494 | 0.883811034 | 1.418234555 | 2.46E-05 | 5.36E-05 |
| AC007608.2 | 0.001369514 | 0.627810201 | 8.840520877 | 1.39E-07 | 4.25E-07 |
| LINC01136 | 0.020872153 | 0.043992215 | 1.075668815 | 0.00088334 | 0.00150714 |
| AC092301.1 | 0.150945678 | 0.370521849 | 1.295529166 | 0.00019728 | 0.00037083 |
| AC079467.1 | 0.110824926 | 0.016474013 | -2.750018472 | 1.29E-34 | 5.86E-32 |
| AC010336.1 | 0.007310612 | 0.027585271 | 1.915834089 | 9.61E-11 | 4.69E-10 |
| LINC01010 | 0.012270623 | 0.051091906 | 2.057886228 | 0.00523355 | 0.00784242 |
| AC015922.3 | 6.034200771 | 2.2584217 | -1.417847803 | 7.25E-19 | 1.30E-17 |
| AL031600.1 | 0.264144738 | 0.884868982 | 1.744135187 | 2.50E-11 | 1.33E-10 |
| LINC02321 | 0.098735454 | 0.401838341 | 2.024975097 | 2.68E-07 | 7.85E-07 |
| LINC01979 | 0.049141191 | 0.448720169 | 3.190811308 | 5.96E-20 | 1.40E-18 |
| MESTIT1 | 0.073356401 | 0.169215252 | 1.205864835 | 3.43E-06 | 8.53E-06 |
| AC078883.2 | 0.03154029 | 0.368445787 | 3.546184533 | 0.00173872 | 0.00283191 |
| AL031275.1 | 0.08781433 | 0.61185426 | 2.800659767 | 3.04E-05 | 6.48E-05 |
| AC011611.3 | 0.017773202 | 0.229305148 | 3.689493199 | 2.49E-14 | 2.06E-13 |
| AL645608.6 | 0.026187873 | 0.174761842 | 2.73841944 | 0.00313992 | 0.0048948 |
| AL590438.1 | 0.137943695 | 0.494338631 | 1.841420139 | 8.68E-09 | 3.15E-08 |
| LINC01412 | 0.119762135 | 0.022490862 | -2.412761012 | 6.02E-24 | 4.00E-22 |
| AC023202.1 | 0.10061522 | 0.048839809 | -1.042719099 | 4.54E-06 | 1.11E-05 |
| AC133644.2 | 0.998000281 | 0.374661415 | -1.413452816 | 3.47E-14 | 2.80E-13 |
| AC010997.4 | 0.046076357 | 0.209985227 | 2.188189277 | 1.96E-06 | 5.06E-06 |
| LINC01762 | 0.041851825 | 0.105116541 | 1.328627283 | 0.00031691 | 0.0005801 |
| AC022079.1 | 0.036535764 | 0.22414756 | 2.617067496 | 0.00793508 | 0.01151104 |
| AC124067.3 | 0.109940413 | 0.441286906 | 2.004995138 | 4.72E-07 | 1.34E-06 |
| LINC02188 | 0.00307384 | 0.065908769 | 4.42235633 | 3.73E-07 | 1.07E-06 |
| AL354733.2 | 0.009680693 | 0.054526768 | 2.493782381 | 0.00583882 | 0.00869208 |
| LINC02519 | 0.017099959 | 0.06033269 | 1.818947065 | 3.11E-05 | 6.62E-05 |
| AL139349.1 | 0.324389466 | 1.049890098 | 1.694439441 | 1.56E-08 | 5.46E-08 |
| AL022322.1 | 0.724793147 | 1.689926054 | 1.221318901 | 9.91E-08 | 3.09E-07 |
| CR559946.2 | 0.032703737 | 0.066782542 | 1.0300155 | 0.03051875 | 0.03995648 |
| LINC01990 | 0.044359421 | 0.016021827 | -1.469201849 | 8.07E-08 | 2.54E-07 |
| AC106881.1 | 0.14590567 | 0.04840365 | -1.5918482 | 1.24E-20 | 3.42E-19 |
| AC007637.1 | 1.11773295 | 0.496414299 | -1.170958963 | 2.74E-18 | 4.45E-17 |
| FAM225B | 0.012993799 | 0.029977478 | 1.206055735 | 0.00182747 | 0.0029623 |
| LINC02585 | 0.489785498 | 1.119724258 | 1.192921536 | 9.70E-08 | 3.03E-07 |
| AC126696.3 | 0.038992329 | 0.124555589 | 1.675527519 | 1.31E-06 | 3.46E-06 |
| AC007163.1 | 0.00930882 | 0.059533684 | 2.677035965 | 0.00029403 | 0.00054021 |
| FO393418.1 | 0.357746832 | 0.132817978 | -1.429488549 | 3.55E-14 | 2.86E-13 |
| AC026704.1 | 0.125083224 | 0.637651053 | 2.349878837 | 3.04E-18 | 4.92E-17 |
| AC079160.1 | 0.041841058 | 0.122133521 | 1.545467965 | 0.00279118 | 0.00438498 |
| AC012055.1 | 0.033390878 | 0.003925153 | -3.088633178 | 2.97E-23 | 1.62E-21 |
| AC026979.1 | 0.013497579 | 0.078249754 | 2.535385561 | 2.28E-05 | 4.99E-05 |
| AC007216.2 | 0.103023329 | 0.378380521 | 1.876866754 | 2.12E-05 | 4.67E-05 |
| AOAH-IT1 | 0.055957639 | 0.323389145 | 2.530864242 | 0.00574048 | 0.00855269 |
| DKFZp779M0652 | 0.328274303 | 0.088637627 | -1.888910651 | 1.52E-17 | 2.18E-16 |
| AC010536.1 | 0.094674353 | 0.236095147 | 1.318322834 | 6.13E-11 | 3.08E-10 |
| AC112484.3 | 0.126701249 | 0.446667572 | 1.817770774 | 0.00082759 | 0.00142004 |
| LINC01426 | 0.054975547 | 0.229963543 | 2.064543202 | 8.68E-09 | 3.15E-08 |
| LINC02029 | 0.002765883 | 0.034499618 | 3.640768485 | 4.62E-09 | 1.74E-08 |
| AC126178.1 | 0.532384077 | 0.090314817 | -2.55943282 | 5.45E-19 | 1.01E-17 |
| AC091078.1 | 0.01753041 | 0.006705196 | -1.386508401 | 5.21E-16 | 5.73E-15 |
| AC012360.3 | 0.282228517 | 0.628925455 | 1.156025258 | 1.64E-10 | 7.79E-10 |
| AC108676.1 | 0.044761162 | 0.247234507 | 2.465560717 | 0.02238147 | 0.03000889 |
| MIR222HG | 0.637629317 | 1.648395524 | 1.370272584 | 3.49E-08 | 1.16E-07 |
| AL136526.1 | 0.022666086 | 0.074237311 | 1.711609153 | 0.01904888 | 0.02584562 |
| AC079015.1 | 0.207563537 | 0.082099452 | -1.338108531 | 5.14E-12 | 3.04E-11 |
| CDKN2B-AS1 | 10.56358258 | 0.363516873 | -4.860933059 | 1.01E-25 | 1.25E-23 |
| AL137246.1 | 0.014445918 | 0.07327821 | 2.342722339 | 0.00629952 | 0.00931686 |
| AC007773.1 | 0.162727149 | 0.589761004 | 1.857675464 | 3.70E-15 | 3.51E-14 |
| ABALON | 0.181470039 | 0.788611395 | 2.119583183 | 4.34E-23 | 2.30E-21 |
| AL121601.1 | 0.218963385 | 0.575035074 | 1.392960316 | 4.82E-06 | 1.18E-05 |
| MANEA-DT | 0.085529704 | 0.199215598 | 1.219833152 | 0.00143248 | 0.00236349 |
| AC124319.1 | 0.11919605 | 1.816816728 | 3.930004556 | 1.33E-18 | 2.28E-17 |
| AL117382.1 | 2.649522241 | 10.89373585 | 2.039694647 | 1.00E-08 | 3.59E-08 |
| AC010186.4 | 0.039448936 | 0.011912189 | -1.727547873 | 1.96E-08 | 6.75E-08 |
| AL162741.1 | 0.055701531 | 0.12107922 | 1.120162389 | 2.15E-06 | 5.52E-06 |
| AC132872.2 | 0.030752973 | 0.109462284 | 1.831636084 | 4.15E-11 | 2.14E-10 |
| LINC01675 | 0.025661815 | 0.086999458 | 1.761383219 | 0.00052366 | 0.00092531 |
| ARNTL2-AS1 | 0.002306754 | 0.111894354 | 5.600129009 | 1.13E-07 | 3.48E-07 |
| AC024451.4 | 0.051826941 | 0.206539751 | 1.994645317 | 0.00272114 | 0.00428483 |
| AL160291.1 | 0.019144621 | 0.044681107 | 1.222725838 | 0.02357671 | 0.03151074 |
| AC010422.4 | 0.137538051 | 0.385147411 | 1.485579919 | 0.00278593 | 0.00437801 |
| PRC1-AS1 | 0.066578005 | 0.172054219 | 1.369745719 | 9.46E-05 | 0.00018697 |
| AC128709.3 | 0.070802163 | 0.029424849 | -1.266758429 | 1.79E-05 | 3.97E-05 |
| AC022893.2 | 0.06083623 | 0.019835539 | -1.616843157 | 9.71E-21 | 2.79E-19 |
| LRP4-AS1 | 0.182780034 | 0.519675116 | 1.507501497 | 0.00316923 | 0.00493625 |
| AC046195.1 | 0.014728302 | 0.00520744 | -1.499944995 | 8.30E-20 | 1.85E-18 |
| AL355388.2 | 0.16854642 | 0.350498744 | 1.05626329 | 1.97E-06 | 5.08E-06 |
| AC008264.2 | 0.011324534 | 0.05955641 | 2.39480504 | 2.47E-08 | 8.37E-08 |
| LINCR-0001 | 0.096695766 | 0.575073088 | 2.572220704 | 2.48E-05 | 5.38E-05 |
| AL669970.3 | 0.100209045 | 0.027328279 | -1.874546237 | 5.25E-18 | 8.11E-17 |
| AC083805.2 | 0.093818735 | 0.391059849 | 2.059441462 | 0.00041617 | 0.00074847 |
| AC062037.2 | 0.397532227 | 0.802916889 | 1.01417884 | 0.01916945 | 0.0259898 |
| CPNE8-AS1 | 0.569155809 | 0.225321825 | -1.336836588 | 2.83E-16 | 3.26E-15 |
| AL645940.1 | 0.100593217 | 0.275744832 | 1.454800826 | 1.28E-06 | 3.40E-06 |
| AL390729.1 | 0.163858273 | 1.186897471 | 2.856674891 | 3.83E-17 | 5.10E-16 |
| AC011815.2 | 0.03813829 | 0.098644783 | 1.371002588 | 0.01285113 | 0.01799928 |
| LINC02257 | 0.002565944 | 0.336836817 | 7.03641637 | 8.12E-17 | 1.03E-15 |
| AL391335.1 | 0.338625774 | 0.159867681 | -1.082813477 | 4.69E-07 | 1.33E-06 |
| AL138820.1 | 0.052069941 | 0.170038272 | 1.707336831 | 0.00658072 | 0.00969332 |
| AL513123.1 | 0.027704836 | 0.343589014 | 3.632474173 | 2.43E-13 | 1.74E-12 |
| AF127577.5 | 0.093237378 | 0.04545522 | -1.03646247 | 5.28E-08 | 1.71E-07 |
| AC091153.3 | 0.188444208 | 0.494453257 | 1.391696692 | 2.47E-08 | 8.37E-08 |
| TRPC7-AS1 | 1.702692316 | 0.448699652 | -1.923995786 | 7.47E-21 | 2.21E-19 |
| AL080312.2 | 0.014081055 | 0.089579857 | 2.669418948 | 0.00083694 | 0.00143438 |
| TSPEAR-AS1 | 0.056366751 | 0.279858877 | 2.311783187 | 2.56E-08 | 8.65E-08 |
| SRD5A3-AS1 | 0.066400846 | 0.13308524 | 1.00307705 | 5.28E-06 | 1.28E-05 |
| LINC00449 | 0.068915197 | 0.196248358 | 1.509786513 | 2.53E-09 | 9.95E-09 |
| AF124730.1 | 0.04029548 | 0.215497237 | 2.418979463 | 0.00028074 | 0.00051719 |
| HCG21 | 0.046543914 | 0.016733354 | -1.475865888 | 1.38E-08 | 4.88E-08 |
| AL021392.1 | 0.02489887 | 0.112046141 | 2.169940754 | 9.91E-14 | 7.52E-13 |
| AC089999.2 | 0.2123924 | 0.465639097 | 1.132480057 | 0.03881382 | 0.04992965 |
| AL139819.1 | 0.07094577 | 0.03213254 | -1.142681656 | 4.00E-06 | 9.88E-06 |
| AC026391.1 | 0.290794337 | 0.016265056 | -4.160151448 | 4.01E-43 | 1.09E-39 |
| AC024361.3 | 0.141275982 | 0.382659089 | 1.437543448 | 1.87E-06 | 4.84E-06 |
| AL133153.2 | 0.09747691 | 0.281565011 | 1.530335641 | 2.46E-06 | 6.28E-06 |
| MIR1-1HG-AS1 | 0.732478663 | 0.090335394 | -3.019423477 | 4.19E-21 | 1.31E-19 |
| AL442067.1 | 0.115027233 | 0.793537785 | 2.786323451 | 5.17E-08 | 1.68E-07 |
| LINC01664 | 0.017055827 | 0.035335525 | 1.050854591 | 0.02408525 | 0.03212741 |
| AC008735.2 | 1.351688507 | 4.689868684 | 1.794784804 | 3.27E-10 | 1.47E-09 |
| AP000266.1 | 0.029228381 | 0.144562294 | 2.306249461 | 0.01917649 | 0.0259929 |
| AC025171.4 | 0.170197833 | 0.350272839 | 1.041266455 | 0.00086463 | 0.00147847 |
| LINC02014 | 0.634519515 | 1.387471125 | 1.128721306 | 9.18E-11 | 4.50E-10 |
| AC092720.2 | 0.014636958 | 0.00378017 | -1.953092633 | 7.06E-24 | 4.58E-22 |
| AC074131.1 | 0.099386575 | 0.028678414 | -1.793085739 | 2.21E-15 | 2.18E-14 |
| AC093607.1 | 0.475429973 | 0.05890472 | -3.01277771 | 1.92E-25 | 2.10E-23 |
| AP001628.1 | 0.211334444 | 0.82732522 | 1.968926638 | 5.51E-09 | 2.06E-08 |
| AC010980.2 | 0.247429143 | 0.093326799 | -1.40665211 | 2.76E-08 | 9.27E-08 |
| UCA1 | 0.743773222 | 12.99922909 | 4.12741945 | 7.23E-15 | 6.54E-14 |
| C11orf72 | 0.009852745 | 0.023806681 | 1.272768867 | 0.01603943 | 0.02206433 |
| AC099329.1 | 0.020695072 | 0.008153768 | -1.343748408 | 4.91E-12 | 2.92E-11 |
| LINC02561 | 0.048784465 | 0.173065219 | 1.826822104 | 5.91E-05 | 0.00012073 |
| AP000439.2 | 14.19173278 | 5.545353869 | -1.355699318 | 4.33E-16 | 4.84E-15 |
| RFPL1S | 0.04530532 | 0.018391278 | -1.300658686 | 9.57E-15 | 8.48E-14 |
| AC144652.1 | 0.429383456 | 1.067163689 | 1.313442974 | 1.16E-15 | 1.21E-14 |
| AC133540.1 | 0.025395376 | 0.252016536 | 3.310880629 | 3.72E-06 | 9.21E-06 |
| ISM1-AS1 | 0.019644893 | 0.103137898 | 2.392348317 | 0.00190959 | 0.00308716 |
| KIAA1614-AS1 | 0.030395805 | 0.061670657 | 1.020712018 | 0.0101936 | 0.01449809 |
| LINC01605 | 0.185751216 | 1.808682125 | 3.283495315 | 1.23E-22 | 5.61E-21 |
| AC092652.1 | 0.312126064 | 0.060008546 | -2.37888895 | 5.67E-35 | 2.81E-32 |
| AC145423.2 | 0.112818524 | 0.602597888 | 2.417191644 | 9.14E-14 | 6.98E-13 |
| GAPLINC | 0.213895969 | 0.661280088 | 1.628352166 | 9.02E-13 | 6.05E-12 |
| AC020931.1 | 0.043518978 | 0.146394725 | 1.75014699 | 3.71E-09 | 1.43E-08 |
| AC113139.1 | 0.154887132 | 0.4081186 | 1.397771173 | 0.00048678 | 0.00086603 |
| LINC02568 | 3.123320072 | 0.481473646 | -2.697551684 | 3.57E-21 | 1.14E-19 |
| AC124017.1 | 0.013262785 | 0.006560145 | -1.015584158 | 3.65E-09 | 1.41E-08 |
| AC034229.4 | 0.130074505 | 0.34596228 | 1.411276536 | 0.00305862 | 0.00477625 |
| AL110115.1 | 0.043257143 | 0.422185599 | 3.286867091 | 2.53E-05 | 5.47E-05 |
| AC137834.2 | 0.011579538 | 0.067238165 | 2.537702608 | 4.25E-06 | 1.05E-05 |
| AC073320.1 | 0.005176352 | 0.025634123 | 2.308057814 | 0.00010788 | 0.00021092 |
| AC211433.2 | 0.044098115 | 0.175402694 | 1.991882025 | 7.14E-08 | 2.27E-07 |
| LINC01376 | 0.104170702 | 0.260852556 | 1.324284998 | 8.11E-06 | 1.90E-05 |
| BX284668.6 | 0.046734101 | 0.136303465 | 1.544274684 | 0.00122098 | 0.00203236 |
| LINC00216 | 0.092829475 | 0.491052702 | 2.403223004 | 0.00874966 | 0.01259554 |
| BLACAT1 | 0.045403916 | 2.118730792 | 5.544239756 | 1.20E-24 | 1.02E-22 |
| AC106869.1 | 1.451494103 | 0.289661316 | -2.325099779 | 9.72E-23 | 4.57E-21 |
| AC008551.1 | 0.031156098 | 0.336985037 | 3.435098065 | 0.00036411 | 0.00066095 |
| AC061992.1 | 0.109149606 | 0.225527771 | 1.046998173 | 1.90E-06 | 4.90E-06 |
| LINC00562 | 0.066813712 | 0.194584862 | 1.542183348 | 1.68E-06 | 4.37E-06 |
| AC016773.1 | 0.332564728 | 0.919138123 | 1.466646516 | 1.09E-12 | 7.20E-12 |
| AC023158.2 | 0.04168525 | 0.012929787 | -1.688838461 | 1.71E-19 | 3.62E-18 |
| AL445423.1 | 0.173900863 | 0.058111145 | -1.581378292 | 2.04E-14 | 1.70E-13 |
| AC005180.2 | 1.439836072 | 0.207579896 | -2.794165935 | 9.76E-21 | 2.79E-19 |
| AC137630.3 | 0.237785036 | 0.608554327 | 1.355728132 | 0.00360471 | 0.00555582 |
| AC005072.1 | 0.009870996 | 0.195113747 | 4.304975896 | 0.02203775 | 0.02959406 |
| AC008026.3 | 0.016959227 | 0.13214921 | 2.962025453 | 0.00285467 | 0.00447958 |
| AC073529.1 | 0.065243317 | 0.20136629 | 1.625920159 | 1.98E-14 | 1.66E-13 |
| AC096751.2 | 1.033198024 | 0.49456583 | -1.062882319 | 9.26E-10 | 3.90E-09 |
| LINC02269 | 0.068491638 | 0.015562474 | -2.137856428 | 1.70E-08 | 5.91E-08 |
| LINC02086 | 6.619474396 | 13.60896016 | 1.039768264 | 1.44E-05 | 3.26E-05 |
| AC007336.1 | 0.032999528 | 0.16024602 | 2.279771234 | 0.00019888 | 0.0003737 |
| LINC01235 | 0.23604095 | 0.635877099 | 1.429710782 | 1.79E-06 | 4.64E-06 |
| AF064858.1 | 0.427910199 | 1.38941534 | 1.699097961 | 0.00245665 | 0.00389763 |
| AC009269.5 | 0.094286209 | 0.570440808 | 2.596958513 | 0.00092791 | 0.00157531 |
