# Supplementary material for: A novel necroptosis-related lncRNA signature for predicting prognosis and immune response of colon cancer
Source: Front Genet. 2022 Aug 25;13:984696. doi: 10.3389/fgene.2022.984696 (PMC9453677; doi:10.3389/fgene.2022.984696)
Supplement: Supplementary file 3 [file Table3.DOCX]

| **Table S 3. Differentially expressed Necroptosis-related lncRNAs** | | | | |
| --- | --- | --- | --- | --- |
| **Necroptosis- related gene** | **lncRNA** | **Correlation**  **coefficients** | ***P* value** | **Regulation** |
| TRAF5 | AL138963.1 | 0.430959791 | 8.19E-23 | positive |
| TRAF5 | AC020907.4 | 0.517265926 | 1.01E-33 | positive |
| TRAF5 | AC008115.3 | 0.481621168 | 7.72E-29 | positive |
| TRAF5 | Z68871.1 | 0.447525498 | 1.13E-24 | positive |
| TRAF5 | AL591895.1 | 0.497243704 | 6.56E-31 | positive |
| TRAF5 | THUMPD3-AS1 | 0.619095975 | 2.18E-51 | positive |
| TRAF5 | PRR7-AS1 | 0.424586663 | 4.00E-22 | positive |
| TRAF5 | AC058791.1 | 0.37136261 | 6.49E-17 | positive |
| TRAF5 | AC079907.1 | 0.455974234 | 1.16E-25 | positive |
| TRAF5 | MIR3142HG | 0.333721348 | 9.09E-14 | positive |
| TRAF5 | AC087277.2 | 0.450926928 | 4.55E-25 | positive |
| TRAF5 | AC245884.8 | 0.478451672 | 1.97E-28 | positive |
| TRAF5 | EIF1AX-AS1 | 0.408980879 | 1.69E-20 | positive |
| TRAF5 | AP000692.1 | 0.345710853 | 1.00E-14 | positive |
| TRAF5 | HM13-IT1 | 0.390084939 | 1.22E-18 | positive |
| TRAF5 | NEAT1 | 0.34286345 | 1.71E-14 | positive |
| TRAF5 | AC099518.2 | 0.364404884 | 2.67E-16 | positive |
| TRAF5 | AC103591.3 | 0.432440376 | 5.64E-23 | positive |
| TRAF5 | GK-AS1 | 0.307198204 | 8.54E-12 | positive |
| TRAF5 | ARHGEF38-IT1 | 0.330987911 | 1.48E-13 | positive |
| TRAF5 | AC073487.1 | 0.37340836 | 4.26E-17 | positive |
| TRAF5 | SCARNA9 | 0.446765138 | 1.38E-24 | positive |
| TRAF5 | AL928654.2 | 0.318604638 | 1.28E-12 | positive |
| TRAF5 | AP002336.2 | 0.455480584 | 1.33E-25 | positive |
| TRAF5 | CD44-AS1 | 0.398359089 | 1.94E-19 | positive |
| TRAF5 | AC092535.4 | 0.311937333 | 3.92E-12 | positive |
| TRAF5 | AP000873.2 | 0.482951122 | 5.19E-29 | positive |
| TRAF5 | LINC01811 | 0.444715523 | 2.37E-24 | positive |
| TRAF5 | ZDHHC20-IT1 | 0.303781728 | 1.48E-11 | positive |
| TRAF5 | AC002128.2 | 0.387322936 | 2.23E-18 | positive |
| TRAF5 | AC087588.2 | 0.336959678 | 5.06E-14 | positive |
| TRAF5 | AC015849.3 | 0.475655686 | 4.47E-28 | positive |
| TRAF5 | LINC01355 | 0.55622305 | 9.08E-40 | positive |
| TRAF5 | AC008760.1 | 0.472470884 | 1.13E-27 | positive |
| TRAF5 | AL353804.2 | 0.44907756 | 7.47E-25 | positive |
| TRAF5 | AL354993.2 | 0.402734882 | 7.17E-20 | positive |
| TRAF5 | AC078860.1 | 0.548987705 | 1.38E-38 | positive |
| TRAF5 | AC132192.2 | 0.492216819 | 3.13E-30 | positive |
| TRAF5 | AC092119.2 | 0.501326966 | 1.81E-31 | positive |
| TRAF5 | PSPC1-AS2 | 0.487531422 | 1.31E-29 | positive |
| TRAF5 | AL109614.1 | 0.482496937 | 5.95E-29 | positive |
| TRAF5 | AL161891.1 | 0.480980591 | 9.34E-29 | positive |
| TRAF5 | AL078587.1 | 0.431977297 | 6.34E-23 | positive |
| TRAF5 | AC010761.1 | 0.421459294 | 8.61E-22 | positive |
| TRAF5 | AC116407.2 | 0.348718006 | 5.69E-15 | positive |
| TRAF5 | AL357079.1 | 0.42431735 | 4.28E-22 | positive |
| TRAF5 | AC005261.1 | 0.527047359 | 3.61E-35 | positive |
| TRAF5 | AC009032.1 | 0.478573383 | 1.90E-28 | positive |
| TRAF5 | AC018653.3 | 0.379854132 | 1.10E-17 | positive |
| TRAF5 | TSPOAP1-AS1 | 0.567006804 | 1.38E-41 | positive |
| TRAF5 | AC131971.1 | 0.428312316 | 1.59E-22 | positive |
| TRAF5 | AC078778.1 | 0.488471028 | 9.84E-30 | positive |
| TRAF5 | AL035071.1 | 0.507218967 | 2.74E-32 | positive |
| TRAF5 | AC000123.1 | 0.546060535 | 4.09E-38 | positive |
| TRAF5 | AC020915.2 | 0.358337349 | 8.89E-16 | positive |
| TRAF5 | AC026356.1 | 0.398517083 | 1.87E-19 | positive |
| TRAF5 | AL049539.1 | 0.451008774 | 4.45E-25 | positive |
| TRAF5 | AC020659.1 | 0.349980124 | 4.48E-15 | positive |
| TRAF5 | AC004253.1 | 0.49534682 | 1.19E-30 | positive |
| TRAF5 | AC108058.1 | 0.370344718 | 8.00E-17 | positive |
| TRAF5 | AL353150.1 | 0.31950443 | 1.10E-12 | positive |
| TRAF5 | LMO7-AS1 | 0.397183872 | 2.52E-19 | positive |
| TRAF5 | AL157838.1 | 0.442607967 | 4.13E-24 | positive |
| TRAF5 | AL590483.1 | 0.340213957 | 2.79E-14 | positive |
| TRAF5 | AC016831.4 | 0.406103563 | 3.30E-20 | positive |
| TRAF5 | AL355075.2 | 0.531525101 | 7.60E-36 | positive |
| TRAF5 | AC087741.1 | 0.399334707 | 1.55E-19 | positive |
| TRAF5 | AL035071.2 | 0.444294249 | 2.65E-24 | positive |
| TRAF5 | CASC9 | 0.339437076 | 3.22E-14 | positive |
| TRAF5 | LINC01473 | 0.485977519 | 2.09E-29 | positive |
| TRAF5 | AP001429.1 | 0.406885126 | 2.76E-20 | positive |
| TRAF5 | AC007038.2 | 0.549847046 | 1.01E-38 | positive |
| TRAF5 | AP001469.3 | 0.472128892 | 1.24E-27 | positive |
| TRAF5 | AC048344.4 | 0.457785286 | 7.05E-26 | positive |
| TRAF5 | TRIM31-AS1 | 0.308754331 | 6.62E-12 | positive |
| TRAF5 | ZKSCAN2-DT | 0.505632756 | 4.57E-32 | positive |
| TRAF5 | AC108134.3 | 0.306090168 | 1.02E-11 | positive |
| TRAF5 | AC093788.1 | 0.516493985 | 1.30E-33 | positive |
| TRAF5 | AL139089.1 | 0.505449707 | 4.85E-32 | positive |
| TRAF5 | AC004837.2 | 0.329765342 | 1.84E-13 | positive |
| TRAF5 | AP006621.4 | 0.405454695 | 3.84E-20 | positive |
| TRAF5 | AC074117.1 | 0.485419398 | 2.48E-29 | positive |
| TRAF5 | Z83843.1 | 0.444627449 | 2.43E-24 | positive |
| TRAF5 | AC004593.1 | 0.408408212 | 1.93E-20 | positive |
| TRAF5 | AC092171.4 | 0.300210476 | 2.62E-11 | positive |
| TRAF5 | LINC00106 | 0.333411268 | 9.61E-14 | positive |
| TRAF5 | AC026368.1 | 0.452053071 | 3.36E-25 | positive |
| TRAF5 | MIR17HG | 0.475115565 | 5.23E-28 | positive |
| TRAF5 | LINC02595 | 0.454557064 | 1.70E-25 | positive |
| TRAF5 | LINC02563 | 0.300255842 | 2.61E-11 | positive |
| TRAF5 | HIF1A-AS2 | 0.303633756 | 1.52E-11 | positive |
| TRAF5 | AL161729.4 | 0.498151723 | 4.94E-31 | positive |
| TRAF5 | AC068790.5 | 0.427717019 | 1.84E-22 | positive |
| TRAF5 | AC007128.1 | 0.534131805 | 3.03E-36 | positive |
| TRAF5 | STAG3L5P-PVRIG2P-PILRB | 0.387208985 | 2.28E-18 | positive |
| TRAF5 | INE1 | 0.356335799 | 1.32E-15 | positive |
| TRAF5 | AC010719.1 | 0.393166256 | 6.19E-19 | positive |
| TRAF5 | AF117829.1 | 0.511574648 | 6.63E-33 | positive |
| TRAF5 | AL117379.1 | 0.576581301 | 2.94E-43 | positive |
| TRAF5 | AL117381.1 | 0.382532883 | 6.25E-18 | positive |
| TRAF5 | AC092338.1 | 0.452284122 | 3.16E-25 | positive |
| TRAF5 | AL137782.1 | 0.301591804 | 2.11E-11 | positive |
| TRAF5 | AL596223.2 | 0.329030109 | 2.10E-13 | positive |
| TRAF5 | Z82243.1 | 0.42309843 | 5.77E-22 | positive |
| TRAF5 | LINC02418 | 0.302701545 | 1.76E-11 | positive |
| TRAF5 | AC018809.1 | 0.373256358 | 4.39E-17 | positive |
| TRAF5 | DLEU2 | 0.406075998 | 3.32E-20 | positive |
| TRAF5 | AC007938.3 | 0.465271628 | 8.78E-27 | positive |
| TRAF5 | ALG13-AS1 | 0.492166227 | 3.17E-30 | positive |
| TRAF5 | AC141002.1 | 0.438856582 | 1.09E-23 | positive |
| TRAF5 | LINC-PINT | 0.435271948 | 2.75E-23 | positive |
| TRAF5 | AL162724.2 | 0.333266902 | 9.86E-14 | positive |
| TRAF5 | NCBP2-AS1 | 0.52038841 | 3.52E-34 | positive |
| TRAF5 | AC090116.1 | 0.37403447 | 3.74E-17 | positive |
| TRAF5 | AL513327.1 | 0.426595939 | 2.44E-22 | positive |
| TRAF5 | AC024060.1 | 0.599698933 | 1.58E-47 | positive |
| TRAF5 | AC145207.8 | 0.426154639 | 2.72E-22 | positive |
| TRAF5 | SP2-AS1 | 0.444960224 | 2.23E-24 | positive |
| TRAF5 | AC011468.1 | 0.415817852 | 3.36E-21 | positive |
| TRAF5 | AL590723.1 | 0.413542565 | 5.78E-21 | positive |
| TRAF5 | AL133410.1 | 0.44817461 | 9.50E-25 | positive |
| TRAF5 | MACC1-AS1 | 0.447700878 | 1.08E-24 | positive |
| TRAF5 | AC015813.1 | 0.524550387 | 8.53E-35 | positive |
| TRAF5 | AC121761.1 | 0.302354129 | 1.87E-11 | positive |
| TRAF5 | AC091057.1 | 0.319623133 | 1.07E-12 | positive |
| TRAF5 | ZNF433-AS1 | 0.311464868 | 4.24E-12 | positive |
| TRAF5 | AC018695.4 | 0.529533076 | 1.52E-35 | positive |
| TRAF5 | TMEM147-AS1 | 0.341735592 | 2.11E-14 | positive |
| TRAF5 | MALAT1 | 0.302759724 | 1.75E-11 | positive |
| TRAF5 | AL080317.2 | 0.507426925 | 2.56E-32 | positive |
| TRAF5 | AC000061.1 | 0.434587766 | 3.27E-23 | positive |
| TRAF5 | AC004908.2 | 0.478422761 | 1.99E-28 | positive |
| TRAF5 | AP003419.3 | 0.341005996 | 2.41E-14 | positive |
| TRAF5 | AL365277.1 | 0.443997439 | 2.87E-24 | positive |
| TRAF5 | AC010536.2 | 0.317698745 | 1.49E-12 | positive |
| TRAF5 | AC008870.2 | 0.555143122 | 1.37E-39 | positive |
| TRAF5 | SNHG20 | 0.435086216 | 2.88E-23 | positive |
| TRAF5 | AGAP2-AS1 | -0.305614246 | 1.10E-11 | negative |
| TRAF5 | FTX | 0.446564253 | 1.46E-24 | positive |
| TRAF5 | AL031716.1 | 0.354088619 | 2.03E-15 | positive |
| TRAF5 | AC084117.1 | 0.415851904 | 3.33E-21 | positive |
| TRAF5 | AL031673.1 | 0.493232867 | 2.28E-30 | positive |
| TRAF5 | AC087222.1 | 0.325807715 | 3.69E-13 | positive |
| TRAF5 | AC108727.1 | 0.31131179 | 4.34E-12 | positive |
| TRAF5 | ANKRD10-IT1 | 0.527890272 | 2.70E-35 | positive |
| TRAF5 | N4BP2L2-IT2 | 0.482858754 | 5.34E-29 | positive |
| TRAF5 | AC019080.5 | 0.517404227 | 9.60E-34 | positive |
| TRAF5 | AL021578.1 | 0.406381097 | 3.10E-20 | positive |
| TRAF5 | AC005519.1 | 0.506878451 | 3.06E-32 | positive |
| TRAF5 | DLEU1 | 0.485671537 | 2.30E-29 | positive |
| TRAF5 | AL133243.2 | 0.476279862 | 3.73E-28 | positive |
| TRAF5 | SNHG4 | 0.580796941 | 5.20E-44 | positive |
| TRAF5 | AC113143.1 | 0.428621857 | 1.47E-22 | positive |
| TRAF5 | AL163953.1 | 0.386775506 | 2.51E-18 | positive |
| TRAF5 | AC048341.2 | 0.526919869 | 3.78E-35 | positive |
| TRAF5 | AL353796.1 | 0.47789575 | 2.32E-28 | positive |
| TRAF5 | AL031985.3 | 0.303507484 | 1.55E-11 | positive |
| TRAF5 | LINC01138 | 0.466204771 | 6.74E-27 | positive |
| TRAF5 | AC100814.1 | 0.436149462 | 2.19E-23 | positive |
| TRAF5 | AL445222.1 | 0.324522901 | 4.62E-13 | positive |
| TRAF5 | AC091057.4 | 0.394061788 | 5.07E-19 | positive |
| TRAF5 | AC138932.5 | 0.400868026 | 1.10E-19 | positive |
| TRAF5 | AC020978.3 | 0.447264408 | 1.21E-24 | positive |
| TRAF5 | AL121895.2 | 0.375070929 | 3.02E-17 | positive |
| TRAF5 | AL034550.1 | 0.491217083 | 4.25E-30 | positive |
| TRAF5 | AC006042.1 | 0.523242014 | 1.33E-34 | positive |
| TRAF5 | AC243967.2 | 0.422820054 | 6.17E-22 | positive |
| TRAF5 | AP000786.1 | 0.373062319 | 4.57E-17 | positive |
| TRAF5 | AC090739.1 | 0.468627869 | 3.39E-27 | positive |
| TRAF5 | AC016394.1 | 0.450370728 | 5.28E-25 | positive |
| TRAF5 | AL133330.1 | 0.331173937 | 1.43E-13 | positive |
| TRAF5 | AP002907.1 | 0.5035319 | 8.97E-32 | positive |
| TRAF5 | AC010542.5 | 0.476006476 | 4.04E-28 | positive |
| TRAF5 | AC022211.1 | 0.398490538 | 1.88E-19 | positive |
| TRAF5 | AL031670.1 | 0.409364785 | 1.55E-20 | positive |
| TRAF5 | ARHGAP27P1-BPTFP1-KPNA2P3 | 0.417015362 | 2.52E-21 | positive |
| TRAF5 | AP002360.3 | 0.564870935 | 3.20E-41 | positive |
| TRAF5 | LENG8-AS1 | 0.510360767 | 9.87E-33 | positive |
| TRAF5 | AC112496.1 | 0.348029748 | 6.49E-15 | positive |
| TRAF5 | CASC19 | 0.449206679 | 7.21E-25 | positive |
| TRAF5 | AP000866.6 | 0.509379986 | 1.36E-32 | positive |
| TRAF5 | AL354696.1 | 0.414136023 | 5.02E-21 | positive |
| TRAF5 | FMR1-IT1 | 0.447176696 | 1.24E-24 | positive |
| TRAF5 | AC011462.4 | 0.42269632 | 6.36E-22 | positive |
| TRAF5 | AL355488.1 | 0.537086306 | 1.06E-36 | positive |
| TRAF5 | PAN3-AS1 | 0.459761565 | 4.09E-26 | positive |
| TRAF5 | AL356299.2 | 0.507902123 | 2.20E-32 | positive |
| TRAF5 | AL133520.1 | 0.327628516 | 2.69E-13 | positive |
| TRAF5 | AL691482.3 | 0.368777732 | 1.10E-16 | positive |
| TRAF5 | GAS6-AS1 | 0.33570015 | 6.36E-14 | positive |
| TRAF5 | LINC00265 | 0.363225611 | 3.38E-16 | positive |
| TRAF5 | PVT1 | 0.366244446 | 1.84E-16 | positive |
| TRAF5 | LINC01748 | 0.443299028 | 3.45E-24 | positive |
| TRAF5 | AC073957.3 | 0.479733387 | 1.35E-28 | positive |
| TRAF5 | AC010834.3 | 0.552213865 | 4.14E-39 | positive |
| TRAF5 | AP003352.1 | 0.4311161 | 7.87E-23 | positive |
| TRAF5 | RUSC1-AS1 | 0.542350974 | 1.59E-37 | positive |
| TRAF5 | AL138689.1 | 0.386279826 | 2.79E-18 | positive |
| TRAF5 | MCM3AP-AS1 | 0.567954354 | 9.49E-42 | positive |
| TRAF5 | AC123023.1 | 0.517179848 | 1.03E-33 | positive |
| TRAF5 | AL442125.2 | 0.443178588 | 3.56E-24 | positive |
| TRAF5 | GABPB1-AS1 | 0.48724937 | 1.43E-29 | positive |
| TRAF5 | AC079684.1 | 0.361309921 | 4.94E-16 | positive |
| TRAF5 | AP006621.2 | 0.414145874 | 5.01E-21 | positive |
| TRAF5 | FIRRE | 0.311756914 | 4.04E-12 | positive |
| TRAF5 | MIR181A2HG | 0.494696691 | 1.45E-30 | positive |
| TRAF5 | LINC02163 | 0.411934495 | 8.45E-21 | positive |
| TRAF5 | AC092168.2 | 0.421719697 | 8.08E-22 | positive |
| TRAF5 | AL049840.1 | 0.39492498 | 4.18E-19 | positive |
| TRAF5 | ACBD3-AS1 | 0.434968315 | 2.97E-23 | positive |
| TRAF5 | AC007608.2 | 0.370234257 | 8.18E-17 | positive |
| TRAF5 | AL031600.1 | 0.374969991 | 3.08E-17 | positive |
| TRAF5 | AL031275.1 | 0.354272846 | 1.96E-15 | positive |
| TRAF5 | AL139349.1 | 0.360952937 | 5.31E-16 | positive |
| TRAF5 | AL022322.1 | 0.398387744 | 1.93E-19 | positive |
| TRAF5 | AC007637.1 | 0.338973042 | 3.50E-14 | positive |
| TRAF5 | MIR222HG | 0.434619001 | 3.24E-23 | positive |
| TRAF5 | AL117382.1 | 0.368829089 | 1.09E-16 | positive |
| TRAF5 | LINCR-0001 | 0.400358532 | 1.23E-19 | positive |
| TRAF5 | AC062037.2 | 0.355603661 | 1.52E-15 | positive |
| TRAF5 | AL442067.1 | 0.426740482 | 2.35E-22 | positive |
| TRAF5 | AP001628.1 | 0.390727102 | 1.06E-18 | positive |
| TRAF5 | BLACAT1 | 0.350490806 | 4.06E-15 | positive |
| TRAF5 | AC009269.5 | 0.418807551 | 1.64E-21 | positive |
| TNFSF10 | LINC02195 | 0.384314541 | 4.27E-18 | positive |
| TNFSF10 | AC010973.2 | -0.313334791 | 3.10E-12 | negative |
| TNFSF10 | SNHG17 | -0.313368909 | 3.09E-12 | negative |
| TNFSF10 | USP30-AS1 | 0.35225948 | 2.90E-15 | positive |
| TNFSF10 | LINC01871 | 0.349607367 | 4.81E-15 | positive |
| TNFRSF10B | AC107959.3 | 0.348189531 | 6.29E-15 | positive |
| TNFRSF10B | AL365181.3 | 0.342330208 | 1.89E-14 | positive |
| TNFRSF10A | AL022316.1 | 0.302090128 | 1.95E-11 | positive |
| TNFRSF10A | SNHG11 | -0.357246458 | 1.10E-15 | negative |
| TNFRSF10A | PCAT6 | -0.325953354 | 3.60E-13 | negative |
| TLR3 | AC009065.2 | -0.33243261 | 1.15E-13 | negative |
| TLR3 | VPS9D1-AS1 | -0.354507391 | 1.88E-15 | negative |
| TLR3 | FLJ46906 | -0.365023385 | 2.36E-16 | negative |
| TLR3 | SNHG15 | -0.34620967 | 9.14E-15 | negative |
| TLR3 | MNX1-AS1 | -0.31705141 | 1.66E-12 | negative |
| TLR3 | GS1-124K5.4 | -0.302065031 | 1.95E-11 | negative |
| TLR3 | MHENCR | -0.329731477 | 1.85E-13 | negative |
| TLR3 | AC109322.1 | -0.386580671 | 2.62E-18 | negative |
| TLR3 | MIR22HG | 0.328380408 | 2.35E-13 | positive |
| TERT | ASMTL-AS1 | 0.30233434 | 1.87E-11 | positive |
| SMPD1 | SNHG3 | -0.311581268 | 4.15E-12 | negative |
| SMPD1 | SNHG1 | -0.328389778 | 2.35E-13 | negative |
| RBCK1 | AL117332.1 | 0.382586542 | 6.18E-18 | positive |
| RBCK1 | AL121899.1 | 0.358900778 | 7.96E-16 | positive |
| RBCK1 | AC084125.4 | 0.346960902 | 7.94E-15 | positive |
| RBCK1 | MCF2L-AS1 | 0.376223309 | 2.37E-17 | positive |
| PLK1 | MBNL1-AS1 | -0.316540017 | 1.81E-12 | negative |
| PLK1 | AC015922.2 | -0.319202007 | 1.15E-12 | negative |
| PLK1 | MIR100HG | -0.346007243 | 9.50E-15 | negative |
| PLK1 | AC025857.2 | -0.302171424 | 1.92E-11 | negative |
| PLK1 | AC015922.3 | -0.360489312 | 5.82E-16 | negative |
| PLK1 | AC137630.3 | 0.37008824 | 8.43E-17 | positive |
| PGAM5 | FAM222A-AS1 | 0.364382136 | 2.68E-16 | positive |
| PGAM5 | AL135905.1 | -0.300656687 | 2.44E-11 | negative |
| PGAM5 | AC245100.7 | -0.316195131 | 1.92E-12 | negative |
| PGAM5 | AC131009.3 | 0.300898203 | 2.35E-11 | positive |
| MYC | PRRT3-AS1 | 0.339451729 | 3.21E-14 | positive |
| MYC | MAFG-DT | 0.426021249 | 2.81E-22 | positive |
| MYC | AC006329.1 | 0.427607103 | 1.89E-22 | positive |
| MYC | RHPN1-AS1 | 0.383379026 | 5.22E-18 | positive |
| MYC | AL512274.1 | -0.359996884 | 6.41E-16 | negative |
| MYC | LINC01315 | 0.360869909 | 5.40E-16 | positive |
| MYC | SLC12A9-AS1 | 0.317882922 | 1.45E-12 | positive |
| MYC | AC124067.4 | 0.396587018 | 2.89E-19 | positive |
| LEF1 | CRNDE | 0.303765664 | 1.49E-11 | positive |
| LEF1 | AC090709.1 | 0.312733591 | 3.43E-12 | positive |
| LEF1 | AP005271.1 | 0.35414604 | 2.01E-15 | positive |
| KLF9 | LINC02381 | 0.41932558 | 1.45E-21 | positive |
| KLF9 | FENDRR | 0.377430952 | 1.84E-17 | positive |
| KLF9 | AL136115.2 | 0.483016867 | 5.09E-29 | positive |
| KLF9 | AP005899.1 | 0.387009863 | 2.38E-18 | positive |
| KLF9 | AL024508.1 | 0.394696195 | 4.40E-19 | positive |
| KLF9 | AL139289.1 | 0.346113659 | 9.31E-15 | positive |
| KLF9 | AL606834.1 | 0.38294861 | 5.72E-18 | positive |
| KLF9 | AC004241.3 | 0.321820901 | 7.37E-13 | positive |
| KLF9 | GK-IT1 | 0.46376419 | 1.34E-26 | positive |
| KLF9 | LINC01705 | 0.383161511 | 5.46E-18 | positive |
| KLF9 | AC008649.2 | 0.302496171 | 1.82E-11 | positive |
| KLF9 | AC011676.1 | 0.303740676 | 1.49E-11 | positive |
| KLF9 | AC104695.3 | 0.385920702 | 3.02E-18 | positive |
| KLF9 | WNT5A-AS1 | 0.467779821 | 4.32E-27 | positive |
| KLF9 | LINC01614 | 0.461181064 | 2.76E-26 | positive |
| KLF9 | MIR4435-2HG | 0.388014611 | 1.92E-18 | positive |
| KLF9 | SERTAD4-AS1 | 0.384500748 | 4.10E-18 | positive |
| KLF9 | DIO3OS | -0.339924595 | 2.94E-14 | negative |
| KLF9 | LINC01235 | 0.516922565 | 1.13E-33 | positive |
| IL33 | LINC01752 | 0.336069739 | 5.95E-14 | positive |
| IL1B | SNHG22 | 0.30682203 | 9.07E-12 | positive |
| IL1B | AC073611.1 | 0.362092502 | 4.23E-16 | positive |
| IL1B | CYTOR | 0.301228808 | 2.23E-11 | positive |
| IL1B | AC093732.1 | 0.327449483 | 2.77E-13 | positive |
| IL1B | AC025259.3 | 0.338353345 | 3.93E-14 | positive |
| IL1B | LINC02362 | 0.306426606 | 9.68E-12 | positive |
| IL1B | UBE2R2-AS1 | 0.333560001 | 9.36E-14 | positive |
| HSP90AB1 | AC124798.1 | 0.30012425 | 2.66E-11 | positive |
| HSP90AB1 | AC104699.1 | -0.303415981 | 1.57E-11 | negative |
| HSP90AB1 | FOXP4-AS1 | 0.327219564 | 2.89E-13 | positive |
| FAS | AC055717.2 | -0.304768384 | 1.27E-11 | negative |
| FAS | AC092171.3 | -0.336887322 | 5.13E-14 | negative |
| FAS | TUSC8 | -0.335604005 | 6.47E-14 | negative |
| FAS | AC115522.1 | 0.31660542 | 1.79E-12 | positive |
| FAS | AC104958.2 | -0.352438382 | 2.80E-15 | negative |
| FAS | LINC02487 | -0.372611271 | 5.02E-17 | negative |
| FAS | AC073335.2 | -0.315088985 | 2.32E-12 | negative |
| FAS | AL121832.2 | -0.339378007 | 3.25E-14 | negative |
| FAS | LINC00941 | 0.303891552 | 1.46E-11 | positive |
| DNMT1 | AC006333.2 | -0.322176102 | 6.94E-13 | negative |
| DNMT1 | AC125807.2 | 0.322729235 | 6.31E-13 | positive |
| DNMT1 | RBPMS-AS1 | -0.300254192 | 2.61E-11 | negative |
| DNMT1 | AC016888.1 | 0.317756135 | 1.48E-12 | positive |
| DNMT1 | PTOV1-AS2 | 0.309706874 | 5.66E-12 | positive |
| DNMT1 | AC099850.3 | 0.309169572 | 6.18E-12 | positive |
| DNMT1 | B4GALT1-AS1 | -0.409896051 | 1.37E-20 | negative |
| CDKN2A | AC127024.5 | 0.338324217 | 3.95E-14 | positive |
| CDKN2A | AP001453.2 | 0.308387665 | 7.03E-12 | positive |
| CDKN2A | AC127024.4 | 0.337337708 | 4.72E-14 | positive |
| BID | MINCR | 0.325119066 | 4.17E-13 | positive |
| BID | AL359513.1 | 0.376089722 | 2.44E-17 | positive |
| BID | AP002387.1 | 0.453648451 | 2.18E-25 | positive |
| BID | AP001554.1 | 0.31797658 | 1.42E-12 | positive |
| BID | AC112491.1 | 0.317286772 | 1.60E-12 | positive |
| BCL2 | AC010998.3 | 0.343346301 | 1.56E-14 | positive |
